# *APP*, *PSEN1*, and *PSEN2* Variants in Alzheimer’s Disease: Systematic Re-evaluation According to ACMG Guidelines

**DOI:** 10.3389/fnagi.2021.695808

**Published:** 2021-06-18

**Authors:** Xuewen Xiao, Hui Liu, Xixi Liu, Weiwei Zhang, Sizhe Zhang, Bin Jiao

**Affiliations:** ^1^Department of Neurology, Xiangya Hospital, Central South University, Changsha, China; ^2^National Clinical Research Center for Geriatric Disorders, Central South University, Changsha, China; ^3^Engineering Research Center of Hunan Province in Cognitive Impairment Disorders, Central South University, Changsha, China; ^4^Hunan International Scientific and Technological Cooperation Base of Neurodegenerative and Neurogenetic Diseases, Changsha, China; ^5^Key Laboratory of Hunan Province in Neurodegenerative Disorders, Central South University, Changsha, China

**Keywords:** Alzheimer’s disease, ACMG-AMP guidelines, *APP*, *PSEN1*, *PSEN2*, re-evaluation

## Abstract

The strategies of classifying *APP*, *PSEN1*, and *PSEN2* variants varied substantially in the previous studies. We aimed to re-evaluate these variants systematically according to the American college of medical genetics and genomics and the association for molecular pathology (ACMG-AMP) guidelines. In our study, *APP*, *PSEN1*, and *PSEN2* variants were collected by searching Alzforum and PubMed database with keywords “PSEN1,” “PSEN2,” and “APP.” These variants were re-evaluated based on the ACMG-AMP guidelines. We compared the number of pathogenic/likely pathogenic variants of *APP*, *PSEN1*, and *PSEN2*. In total, 66 *APP* variants, 323 *PSEN1* variants, and 63 *PSEN2* variants were re-evaluated in our study. 94.91% of previously reported pathogenic variants were re-classified as pathogenic/likely pathogenic variants, while 5.09% of them were variants of uncertain significance (VUS). *PSEN1* carried the most prevalent pathogenic/likely pathogenic variants, followed by *APP* and *PSEN2*. Significant statistically difference was identified among these three genes when comparing the number of pathogenic/likely pathogenic variants (*P* < 2.2 × 10^–16^). Most of the previously reported pathogenic variants were re-classified as pathogenic/likely pathogenic variants while the others were re-evaluated as VUS, highlighting the importance of interpreting *APP*, *PSEN1*, and *PSEN2* variants with caution according to ACMG-AMP guidelines.

## Introduction

Being the most common neurodegenerative disease, Alzheimer’s disease (AD) is hallmarked by insidious cognitive impairment. It is estimated that 50 million individuals are affected by dementia worldwide and AD accounts for 50–60% of dementia. With the coming of an aging society, the number of AD is increasing rapidly (Saez-Atienzar and Masliah, 2020). To date, only three causative genes have been identified in the pathogenesis of AD, including amyloid precursor protein (*APP*), presenilin1 (*PSEN1*), and presenilin2 (*PSEN2*). *APP* encodes a protein called amyloid-β protein precursor, whose proteolysis generates amyloid-β(Aβ), a key component of amyloid plaque. Additionally, presenilin-1 and presenilin-2 are encoded by *PSEN1* and *PSEN2*, respectively. Both of them are subunits of γ-secretase and associated with either the increase of Aβ or the raised ratio of Aβ42 over Aβ40 (Loy et al., 2014), causing the formation of amyloid plaques and leading to the development of AD (Sun et al., 2017).

The diagnosis criteria of AD are evolving rapidly. Currently, Aβ deposition, pathologic tau, and neurodegeneration [AT(N)] classification system is used to define AD based on biomarker evidence. However, the methods of obtaining biomarkers are expensive or invasive. The identification of pathogenic mutations is still of vital importance in the diagnosis of AD (Jack et al., 2018). The Dominantly Inherited Alzheimer Network (DIAN), funded by the National Institute on Aging (NIA), collected over 450 individuals with 90 different mutations in *PSEN1, PSEN2*, *and APP.* DIAN constitutes a strong impact in AD research because it is remarkably helpful in the understanding of the disease’s natural history (Morris et al., 2012). In the DIAN study, the classification rate between the mutation-carriers group and normal controls is approximately 80% using biomarkers with machine learning (Castillo-Barnes et al., 2020). The DIAN study is not only important for discovering disease trajectories (Luckett et al., 2021), but also for drug trials (Bateman et al., 2017). All the results can be well established only through the correct diagnosis of AD.

Currently, the most frequent cause of AD is variants of *PSEN1*. Variants in *APP* are responsible for the second common cause of AD. By contrast, variants in *PSEN2* leading to AD are relatively rare^1^. Nevertheless, although these three causative genes have been widely investigated, the interpretations of variants remain complex (Hsu et al., 2020). The strategies of classifying variants varied substantially in the previous studies (Denham et al., 2019). Furthermore, the age of onset and clinical manifestations differed among patients with different mutations (Ryan et al., 2016). Subsequently, timely genetic testing and correct classification are fundamental in the diagnosis and treatment of AD patients.

In 2015, the American college of medical genetics and genomics and the association for molecular pathology (ACMG-AMP) issued a guideline for classifying variants based on typical types of variant evidence (e.g., population data, computational data, functional data, segregation data, etc.) (Richards et al., 2015). In our study, to classify these variants systematically and scientifically, we re-evaluate *APP*, *PSEN1*, and *PSEN2* variants according to the ACMG-AMP guidelines. Variants of uncertain significance (VUS) are defined by the criteria for benign and pathogenic are contradictory or the lack of criteria to be classified as pathogenic or benign. Our study re-assessed the *APP*, *PSEN1*, and *PSEN2* variants as well as compared the mutation spectrum of these three genes, which may have important implications in the molecular diagnosis and treatment of AD.

## Materials and Methods

### Systematic Search

We re-analyzed *APP*, *PSEN1*, and *PSEN2* variants from the Alzforum database (see text footnote 1) and searched related literature using PubMed^2^ with the keywords “PSEN1,” “PSEN2,” or “APP.” All the studies included either clinical characteristics or functional data about these variants. All these variants were re-evaluated by two independent investigators according to the ACMG-AMP guidelines (Richards et al., 2015). Permission was obtained from Alzforum to re-analyze the variants in *APP*, *PSEN1*, and *PSEN2* genes.

### Analysis of Variant Frequency

According to the ACMG-AMP guideline, if a variant doesn’t exist in a large general population or a control cohort, it can be considered as pathogenic moderate criterion 2 (PM2). In our study, the variant frequency was searched using Exome Sequencing Project (ESP6500) (Auer et al., 2016), 1,000 Genomes Project (Auton et al., 2015), and the ExAC Browser (Lek et al., 2016). Given that *APP*, *PSEN1* and *PSEN2* are inherited in an autosomal dominant mode in AD, therefore, if a variant was absent from these databases, pathogenic moderate criterion 2 (PM2) can be applied (Li and Wang, 2017). Given that the *APP*, *PSEN1*, and *PSEN2* are not fully penetrant, whereas benign interpretation (BS2) can be established only when the penetrance is 100% at an early age in healthy controls. Subsequently, the BS2 is not applied in the classification of *APP*, *PSEN1*, and *PSEN2* variants (Table 1).

**TABLE 1:** Relevance of ACMG criteria in AD.

ACMG criteria	To be applied	Not to be applied (reason)
Evidence of pathogenicity	PVS1, PS1, PS2, PS3, PS4; PM1, PM2, PM4, PM5, PM6; PP1, PP2, PP3, PP5	PM3 (AD is not a recessive disorder) PP4 (The clinical phenotype of family history of AD is not highly specific)
Evidence of benign impact	BA1, BS1, BS3, BS4; BP3, BP4, BP5, BP6, BP7	BS2 (AD is not fully penetrant at an early age) BP1 (Missense variants primarily cause AD) BP2 (The same reason with BS2)

*PVS, pathogenic very strong; PS, pathogenic strong; PM, pathogenic moderate; PP, pathogenic supporting; BA, benign standalone; BS, benign strong; BP, benign supporting.*

### *In silico* Evidence

The pathogenicity of variants was also predicted using multiple *in silico* genomic tools, including SIFT (Ng and Henikoff, 2003), Polyphen-2 (Adzhubei et al., 2010), LRT (Chun and Fay, 2009), MutationTaster (Schwarz et al., 2010), MutationAssessor (Reva et al., 2011), FATHMM (Shihab et al., 2013), PROVEAN (Choi et al., 2012), CADD (Kircher et al., 2014), REVEL (Ioannidis et al., 2016), and Reve (Li et al., 2018b). For missense variants, we used VarCards^3^, an integrated genetic database, to get the *in silico* prediction results of variants (Li et al., 2018a). For splicing variants, GeneSplicer and Human Splicing Finder were applied to predict the pathogenicity of variants. If all of the genomic tools supported the damaging of variants, then pathogenic supporting criterion 3 (PP3) was established. However, if the prediction results were conflicting, then PP3 would not be used.

### Analysis of Functional Studies

The increased amount of Aβ or raised ratio of Aβ42 over Aβ40 was considered as the key events in the pathogenesis of AD (Selkoe and Hardy, 2016). Consequently, pathogenic strong criterion 2 (PS3) could be applied if a variant leads to elevated total Aβ production or increased Aβ42/Aβ40 ratio in well-established *in vitro* functional studies. The functional data were excluded when there is conflicting evidence among studies. Besides, if functional studies showed that a variant exerts no effect on Aβ production and Aβ42/Aβ40 ratio, then benign strong criterion 3 (BS3) would be taken into consideration.

### Statistical Analyses

We performed a Chi-square test to compare the number of pathogenic/likely pathogenic variants of *APP*, *PSEN1*, and *PSEN2* genes using the SPSS 20.0 test (SPSS, Chicago, IL, United States). Besides, the number of pathogenic/likely pathogenic variants was analyzed between the transmembrane domain and non-transmembrane domain in *APP*, *PSEN1*, and *PSEN2* separately. In the pathogenic/likely pathogenic variants, each variant type of *PSEN1*, *PSEN2*, and *APP* was compared, respectively, by Fisher test. A *p*-value < 0.05 was considered statistically significant.

## Results

### Summary of Variants

**FIGURE 1:**
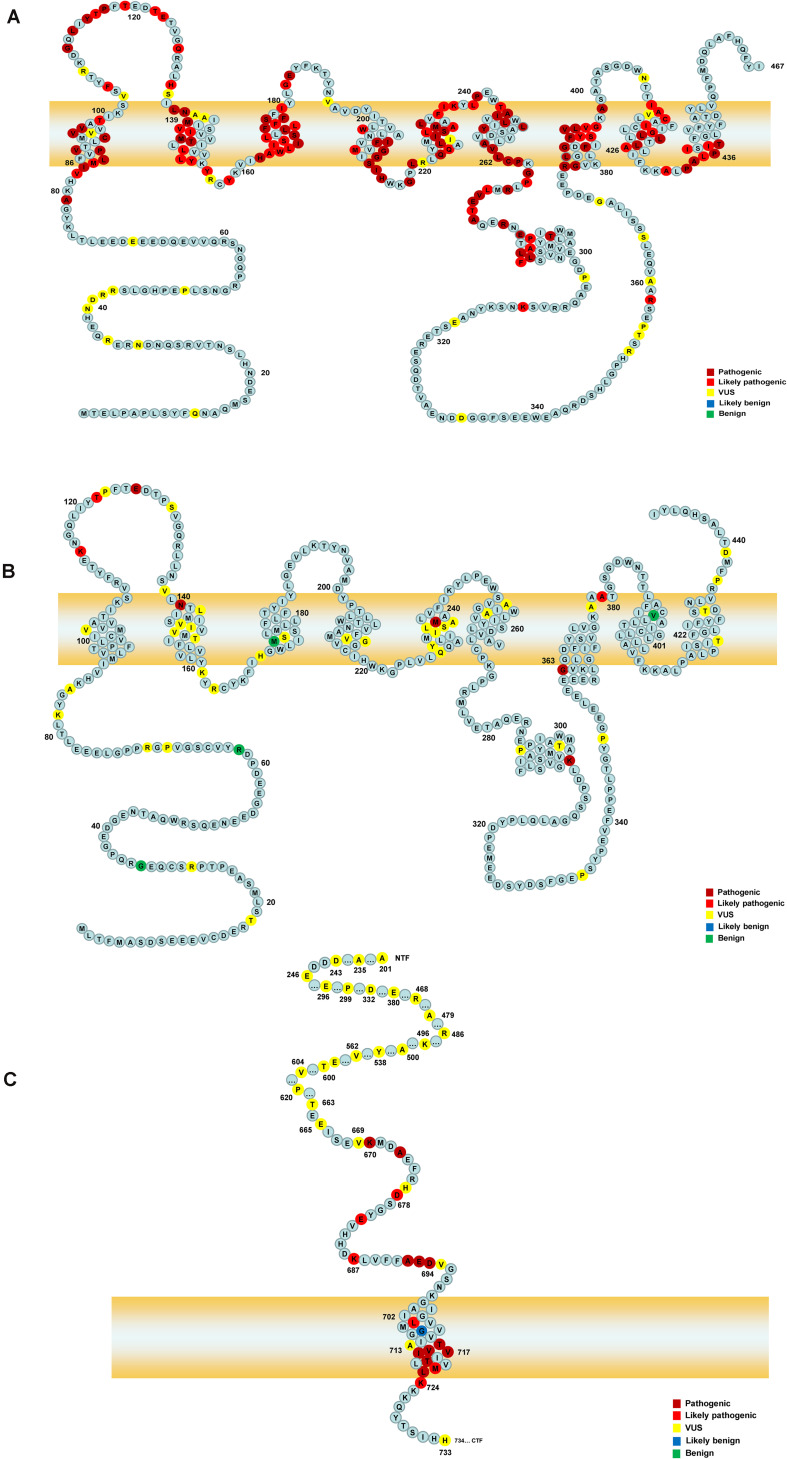
The schematic of PSEN1, PSEN2, and APP proteins. Panels **(A,B)** represent the full-length amino acid sequence of PSEN1 and PSEN2, respectively. Panel **(C)** shows a part of the amino acid sequence of APP. Each circle with colors represents the likelihood of pathogenicity. Crimson circles: Pathogenic variants; Red circles: Likely pathogenic variants; Yellow circles: VUS variants; Blue circles: Likely benign circles; Green circles: Benign variants.

**FIGURE 2:**
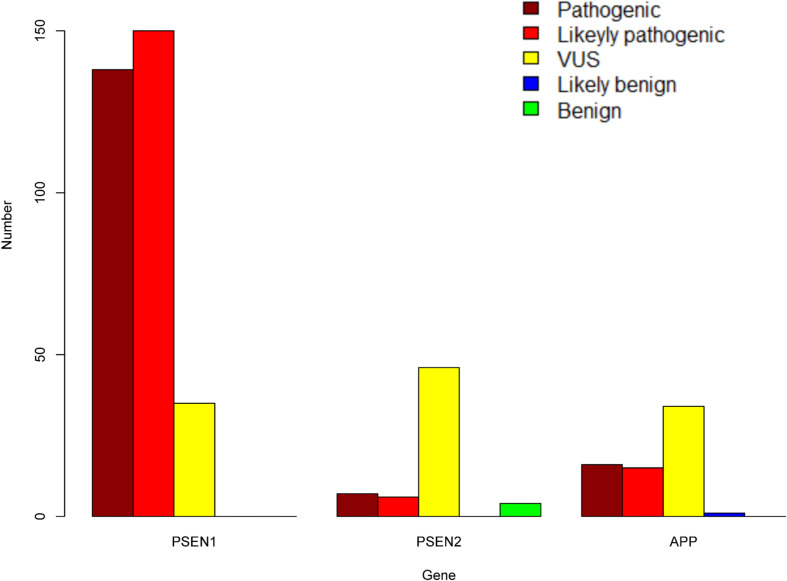
The number of pathogenic, likely pathogenic, VUS, likely benign, and benign variants in *PSEN1*, *PSEN2*, and *APP*.

A total of 452 variants of *APP*, *PSEN1*, and *PSEN2* were collected, in which 66 *APP* variants, 323 *PSEN1* variants, and 63 *PSEN2* variants were re-analyzed in our study (Figure 1 and Supplementary Table 1). *PSEN1* was the most common gene in patients with AD (323/452, 71.24%). According to the ACMG-AMP guidelines, 89.16% *PSEN1* variants (288/323) were re-classified as pathogenic/likely pathogenic variants, followed by *APP* gene, in which 46.97% variants (31/66) were re-considered pathogenic/likely pathogenic variants. The least pathogenic or likely pathogenic variants came from the *PSEN2* gene, among them, only 20.63% of variants (13/63) fulfilled the criteria for pathogenicity/likely pathogenicity (Table 2). Significant differences were observed among these three genes when comparing the number of pathogenic/likely pathogenic variants (*P* < 2.20 × 10^–16^). 317 previously reported pathogenic variants (94.91%) were re-evaluated as pathogenic/likely pathogenic variants, and 17 variants (5.09%) VUS variants. Moreover, the number of pathogenic/likely pathogenic variants in *PSEN1* was more than that of *APP* (*P* = 1.78 × 10^–15^) as well as *PSEN2* (*P* < 2.20 × 10^–16^) (Figure 2). There are more pathogenic/likely pathogenic variants in *APP* than in *PSEN2* (*P* = 3.00 × 10^–3^). Additionally, In the *PSEN1* pathogenic/likely pathogenic variants, 262 missense, 15 indel, six CNV, two splicing, and one frameshift variant were identified. Seven missense, five frameshift, and one splicing variant were found in the *PSEN2* pathogenic/likely pathogenic variants. *APP* pathogenic/likely pathogenic variants consisted of 29 missense and one indel variant. In the pathogenic/likely pathogenic variants, missense variants are more common in *PSEN1* than those in *PSEN2* (*P* = 6.47 × 10^–4^). No difference was observed in the number of missense variants between *PSEN1* and *APP* (*P* = 0.49). More frameshift variants were identified in *PSEN2* than those in *PSEN1* (*P* = 3.92 × 10^–7^). The splicing variant exhibited no difference between *PSEN1* and *PSEN2* (*P* = 0.13). Also, the indel variants in *PSEN1* were similar to those in *APP* (*P* = 1.00).

**TABLE 2:** ACMG classifications of three pathogenic variants in AD.

Gene	Variant types	Transmembrane	ACMG classifications	No.
*APP*	Missense	+	Pathogenic	11
*APP*	Missense	+	Likely pathogenic	6
*APP*	Missense	+	VUS	2
*APP*	Missense	+	Benign	2
*APP*	NA	NA	VUS	2
*APP*	Missense	−	Pathogenic	5
*APP*	Missense	−	Likely pathogenic	7
*APP*	Missense	−	VUS	27
*APP*	Missense	−	Benign	1
*APP*	Indel	−	Likely pathogenic	1
*APP*	Indel	NA	VUS	2
*PSEN1*	Missense	+	Pathogenic	85
*PSEN1*	Missense	+	Likely pathogenic	95
*PSEN1*	Missense	+	VUS	7
*PSEN1*	Missense	−	Pathogenic	37
*PSEN1*	Missense	−	Likely pathogenic	46
*PSEN1*	Missense	−	VUS	25
*PSEN1*	Indel	+	Pathogenic	6
*PSEN1*	Indel	+	Likely pathogenic	5
*PSEN1*	Indel	−	Pathogenic	3
*PSEN1*	Indel	−	Likely pathogenic	2
*PSEN1*	Indel	−	VUS	3
*PSEN1*	Frameshift	−	Pathogenic	1
*PSEN1*	Missense,CNV	NA	Pathogenic	6
*PSEN1*	splicing	NA	Likely pathogenic	2
*PSEN2*	Missense	+	Pathogenic	3
*PSEN2*	Missense	+	Likely pathogenic	3
*PSEN2*	Missense	+	VUS	22
*PSEN2*	Missense	+	Likely benign	1
*PSEN2*	Missense	+	Benign	2
*PSEN2*	Missense	−	Likely pathogenic	1
*PSEN2*	Missense	−	VUS	21
*PSEN2*	Missense	−	Benign	3
*PSEN2*	Splicing	NA	Likely pathogenic	1
*PSEN2*	Frameshift	NA	Pathogenic	2
*PSEN2*	Frameshift	−	Pathogenic	2
*PSEN2*	Frameshift	−	Likely pathogenic	1
*PSEN2*	Frameshift	−	VUS	1

*No., number; CNV, copy number variation; VUS, uncertain significance; NA, not applied.*

### PSEN1

323 *PSEN1* variants were collected in our study, among them, according to the ACMG-AMP guidelines, 138 variants (42.72%) were re-classified as pathogenic variants, 150 variants (46.44%) likely pathogenic variants, and 35 variants (10.84%) VUS variants. 97.21% of previously reported pathogenic variants (279/287) were re-interpreted as pathogenic/likely pathogenic variants. *PSEN1* variants were located in exon 7 (22.29%, *n* = 72), exon 5 (18.89%, *n* = 61), exon 8 (13.00%, *n* = 42),exon 4 (10.84%, *n* = 35), exon 6 (10.53%, *n* = 34), exon 11 (9.29%, *n* = 30), exon 12 (7.12%, *n* = 23), exon 10 (3.10%, *n* = 10), exon 9 (1.55%, *n* = 5), intron 8, exon 9 (1.86%, *n* = 6), intron 8/11 (0.62%, *n* = 2), exon 3 (0.31%, *n* = 1), exons 9–10, introns 8–10 (0.31%, *n* = 1), and intron 4 (0.31%, *n* = 1). Most of *PSEN1* variants (91.95%) were found in exon 7, exon 5, exon 8, exon 6, exon 4, exon 11, and exon 12. No variants were detected in exon 1 and exon 2. There are five types of variants: 295 missense variants (91.33%), 19 indel variants (5.88%), two splicing variants (1.86%), six copy number variation (CNV) (0.62%), and one frameshift variant (0.31%). 198 variants (62.26%) were located in the transmembrane domain and 112 (35.22%) variants in the non-transmembrane domain, while eight variants (2.52%) were not applied. When it comes to the variants in the transmembrane domain, 191 variants were re-classified as pathogenic/likely pathogenic variants and eight variants VUS variants. In contrast, 89 variants in the non-transmembrane domain were re-interpreted as pathogenic/likely pathogenic variants, while 28 variants were VUS variants. The number of pathogenic/likely pathogenic variants differed significantly between transmembrane domain and non-transmembrane domain in *PSEN1* gene (*P* = 2.04 × 10^–7^).

### PSEN2

We re-analyzed 63 *PSEN2* variants in our study. Seven variants (11.11%) were re-classified as pathogenic variants, six variants (9.52%) were likely pathogenic variants, 46 variants (73.02%) were VUS variants, and four variants (6.35%) were benign. Only 50% of previously reported pathogenic variants (8/16) fulfilled the criteria for pathogenicity/likely pathogenicity. With regard to variant type, 56 missense variants (88.89%), six frameshift variants (9.52%), and one splicing variant (1.59%) were identified. These variants belonged to exon 5 (28.57%, *n* = 18), exon 7 (23.81%, *n* = 15), exon 4 (14.29%, *n* = 9), exon 3 (4.76%, *n* = 3), exon 6 (4.76%, *n* = 3), exon 10 (4.76%, *n* = 3), exon 11 (4.76%, *n* = 3), exon 12 (6.35%, *n* = 4), exon 8 (1.59%, *n* = 1), exon 9 (1.59%, *n* = 1), intron 11/12 (3.17%, *n* = 2), and intron 9/12 (1.59%, *n* = 1). No variants were detected in exon 1, exon 2, and exon 3. 31 variants were located in the transmembrane domain and 29 variants in the non-transmembrane domain, while three variants couldn’t be determined. Six variants (20.00%) were re-classified as pathogenic/likely pathogenic variants in the transmembrane domain and four variants (14.29%) were pathogenic/likely pathogenic in the non-transmembrane domain. The number of pathogenic/likely pathogenic variants exhibited no significant difference between the transmembrane domain and non-transmembrane domain (*P* = 0.73).

### APP

A total of 66 *APP* variants were re-evaluated in our study. 16 variants (24.24%) were re-classified as pathogenic variants, 15 likely pathogenic variants (22.73%), 34 VUS variants (51.52%), and one likely benign variant (1.51%) based on the ACMG-AMP guidelines. 96.77% of the previously pathogenic variants (30/31) were re-evaluated as pathogenic/likely pathogenic variants. 61 *APP* variants (92.42%) were missense variants, three variants (4.55%) were indel variants and two variants (3.03%) were located in UTR. These variants were located in exon 17 (45.45%, *n* = 30), exon 16 (18.18%, *n* = 12), exon 14 (7.58%, *n* = 5), exon 6 (4.55%, *n* = 3), exon 7 (4.55%, *n* = 3), exon 11 (4.55%, *n* = 3), exon 12 (3.03%, *n* = 2), exon 13 (3.03%, *n* = 2), exon 5 (1.52%, *n* = 1), exon 9 (1.52%, *n* = 1), 3′UTR (4.55%, *n* = 3), and intron 17 (1.52%, *n* = 1). No variants were found in exon 1, exon 2, exon 3, exon 4, exon 8, exon 10, exon 15, and exon 18. 21 variants were located in the transmembrane domain and 41 variants in the non-transmembrane domain. 17 variants were re-interpreted as pathogenic/likely pathogenic variants in the transmembrane domain while 14 variants were pathogenic/likely pathogenic in the non-transmembrane domain. The number of pathogenic/likely pathogenic variants showed a significant difference between the transmembrane domain and non-transmembrane domain (*P* = 1.01 × 10^–3^).

## Discussion

In our study, for the first time, all *APP*, *PSEN1*, and *PSEN2* variants were re-evaluated systematically on the basis of the ACMG-AMP guidelines. We found that 94.91% of previously reported pathogenic variants were re-evaluated as pathogenic/likely pathogenic variants, and the others were VUS variants. The most prevalent pathogenic/likely pathogenic variants were located in the *PSEN1* gene, followed by the *APP* gene and the *PSEN2* gene. Our study may have important implications in the molecular diagnosis of AD.

Thanks to the rapid development of sequencing technology, an increasing number of studies investigated the *APP*, *PSEN1*, and *PSEN2* genetic spectrum worldwide, demonstrating their significant role in AD pathogenesis (Sassi et al., 2014; Gao et al., 2019). Nevertheless, the classification of variants remains a challenge (Lanoiselée et al., 2017). The heterogeneity of clinical data and the difference in the approaches used for variant interpretation are the major challenges in classifying variants (Denham et al., 2019). The *APP*, *PSEN1*, and *PSEN2* genes have a high rate of rare variants, and the appropriate classification of them is essential in the correct diagnosis and treatment of AD. Furthermore, a high degree of variation in interpreting these variants existed in the previous studies, which may impose negative effects on clinical practice and scientific research. Consequently, re-evaluation of the *APP*, *PSEN1*, and *PSEN2* variants is particularly relevant (Denham et al., 2019). The ACMG-AMP revised standards and guidelines to interpret variants using detailed criteria, such as variant types and population frequency (Richards et al., 2015). The ACMG-AMP guidelines are feasible and standard in classifying variants, which were widely applied in variant classification (Pakhrin et al., 2018; Peng et al., 2018). In some recent AD genetic screening studies, the variants of *APP*, *PSEN1*, and *PSEN2* were also classified based on the ACMG-AMP guidelines (Xu et al., 2018; Jiang et al., 2019). However, to date, no study has evaluated all of the variants in *APP*, *PSEN1* and *PSEN2* reported previously. Consequently, in our study, all of the reported variants in these genes were re-classified according to the ACMG-AMP guidelines.

Our study indicated that most of the previously reported pathogenic variants (317/334) in *APP*, *PSEN1*, and *PSEN2* were still re-classified as pathogenic/likely pathogenic variants, whereas 17 variants were re-evaluated as VUS variants, including one variant in the *APP*, eight variants in the *PSEN1* and eight variants in the *PSEN2*. There are several reasons why the previously reported pathogenic variants were re-classified as VUS variants. Firstly, despite the extensive support evidence of pathogenicity of some variants, they were classified as VUS because some *in silico* prediction tools disagreed on the damaging effects of variants. Secondly, the existence of evidence of benign impact argued against the pathogenicity of variants. A few variants showed no damaging effect on Aβ production, arguing against their pathogenicity. Thirdly, a few variants were interpreted as VUS since they lacked enough evidence of pathogenicity. Take *PSEN2* T122R for example, it fulfilled the criteria of PM2, PM5, and PP3. However, it was classified as VUS since there was no other evidence of pathogenicity. We identified that 25.44% (115/452) of *APP*, *PSEN1*, and *PSEN2* variants were classified as VUS. To assess the VUS variants more accurately, increased collaborations, genetic data sharing, and well-established functional studies may be of great importance in the discovery and classification of VUS.

We demonstrated that the *PSEN1* gene possessed the highest number of pathogenic/likely pathogenic variants, followed by the *APP* gene and the *PSEN2* gene, which was consistent with our previous study. In 404 Chinese AD pedigrees, the most common mutated gene is *PSEN1*, also followed by *APP* and *PSEN2* (Jia et al., 2020). Similarly, DIAN collected autosomal dominant AD globally and re-analyzed the variants’ pathogenicity using available information, showing that two *PSEN1*, one *APP*, and one *PSEN2* are likely pathogenic variants (Hsu et al., 2018). 38 different *PSEN1* mutations and six *APP* mutations were identified in patients with autosomal dominant familial Alzheimer’s disease (Ryan et al., 2016). 76 *PSEN1*, 6 *PSEN2*, and 6 *APP s*ymptomatic mutation carriers were recruited to characterize neuroimaging biomarkers change in DIAN (Gordon et al., 2018). Currently, 265 mutation carriers were included in DIAN, including 202 *PSEN1*, 22 *PSEN2*, and 43 *APP* mutation carriers (Castillo-Barnes et al., 2020; Luckett et al., 2021). In another France AD whole-exome sequencing study, three *PSEN1* and one *PSEN2* likely causative variants were identified (Nicolas et al., 2016). In short, *PSEN1* is the most common pathogenic gene while *APP* or *PSEN2* owns relatively few pathogenic variants in AD.

Our re-analysis found that most *PSEN1* variants are located in exon 7, exon 5, exon 8, exon 6, exon 4, exon 11, and exon 12, most *PSEN2* variants in exon 5, and exon 7, and most *APP* variants in exon 14, exon 16, exon 17, which was consistent with previous studies (Raux et al., 2005; Barber et al., 2016). In the Asain population, variants in exon 4, exon 7, exon 11, exon 14, and exon 17 of the *APP*, exon 4, exon 5, exon 6, exon 7, exon 8, and exon 12 of the *PSEN1*, as well as exon 5, exon 6, and exon 7 of the *PSEN2* gene were discovered between 2009 and 2018 (Giau et al., 2019). In the French population, *PSEN1* variants are located in exon 4, exon 5, exon 6, exon 7, exon 8, exon 9, exon 10, exon 11, and exon 12. Additionally, variants in exon 6 and exon 9 in the *PSEN2* gene as well as variants in exon 17 were identified in AD patients (Lanoiselée et al., 2017). Generally, most of the pathogenic AD mutations are located in exons 16–17 of the *APP*, exons 3–12 of *PSEN1*, and exons 3–12 of *PSEN2* genes (An et al., 2016). These results suggested the above exons are variant hotspots and needed to be given priority when performing DNA sequencing (Zhao and Liu, 2017). Moreover, in the pathogenic/likely pathogenic variants, missense variants are more common in *PSEN1* than those in *PSEN2*. More frameshift variants were identified in *PSEN2* than those in *PSEN1*. These results indicated that the importance of considering these variant type when interpreting variants’ pathogenicity.

Besides, we demonstrated that the number of pathogenic/likely pathogenic variants in the transmembrane domain is significantly higher than that of the non-transmembrane domain in *PSEN1* and *APP*, whereas *PSEN2* exhibited no difference. Wolfe et al. (1999) indicated that transmembrane aspartate of PSEN1 plays an essential role in γ-secretase activity. The APP transmembrane domain contains the γ-secretase site and it can bind with PSEN1, resulting in the production of Aβ (Esselens et al., 2012). *PSEN1* shares a 60% sequence homology with *PSEN2* with highly conserved transmembrane, and both of them are the sub-units of γ-secretase (Escamilla-Ayala et al., 2020). However, PSEN1 lacks the sorting motif found in PSEN2 and expresses broadly at the cell surface and endosomes (Kanatsu et al., 2014; Sannerud et al., 2016). The differences in subcellular localization between PSEN1 and PSEN2 may explain why the variants in the *PSEN2* transmembrane domain are less likely to be pathogenic (Park et al., 2015).

In this study, the increased amount of Aβ or ratio of Aβ42 over Aβ40 in well-established studies was considered a piece of strong evidence of pathogenicity-PS3. It is noteworthy that prominent Aβ42 deposition not only causes senile plaques in the cortex but also may result in amyloid angiopathy in AD patients (Lemere et al., 1996). Despite an increasing number of functional works (Chen et al., 2015; Zhang et al., 2020), the absence of functional data is a major challenge in re-analyzing variants of *APP*, *PSEN1*, and *PSEN2*. Meanwhile, the lack of families’ sequencing results is also an important confounding factor because the *de novo* or co-segregation of a variant cannot be determined (Chen et al., 2012; Dobricic et al., 2012). Of note, only a small proportion of AD patients are caused by *APP*, *PSEN1*, and *PSEN2*. AD is a complex disease that risk genes may interact with environmental factors (Kunkle et al., 2019; Hsu et al., 2020). Thus, variants in AD should be classified carefully as some previously reported pathogenic variants may be risk ones rather than disease-causing in the pathogenesis of AD.

Taken together, in the current study, according to ACMG-AMP guidelines, we systematically re-analyzed the variants in *APP*, *PSEN1*, and *PSEN2*. We found that most of the previously pathogenic variants were re-classified as pathogenic/likely pathogenic variants, whereas a small proportion of previously pathogenic variants were interpreted as VUS variants. *PSEN1* possessed the highest number of pathogenic/likely pathogenic variants, followed by *APP* and *PSEN2*. These findings may underscore the importance of classifying *APP*, *PSEN1*, and *PSEN2* variants with caution.

## Data Availability Statement

The original contributions presented in the study are included in the article/Supplementary Material, further inquiries can be directed to the corresponding author/s.

## Author Contributions

XX and BJ: study design, acquisition of data, analysis, interpretation of data, and drafting/revising the manuscript. HL, XL, WZ, and SZ: acquisition of data. All authors contributed to the article and approved the submitted version.

## Conflict of Interest

The authors declare that the research was conducted in the absence of any commercial or financial relationships that could be construed as a potential conflict of interest.

## Footnotes

1 https://www.alzforum.org/mutations, accessed in March 2021

2 http://ncbi.nlm.nih.gov/pubmed

3 http://varcards.biols.ac.cn/

## Supplementary Material

Supplementary Table 1 The ACMG-AMP classifications of three genes in AD.
