# Supplementary material for: APP, PSEN1, and PSEN2 Variants in Alzheimer’s Disease: Systematic Re-evaluation According to ACMG Guidelines
Source: Front Aging Neurosci. 2021 Jun 18;13:695808. doi: 10.3389/fnagi.2021.695808 (PMC8249733; doi:10.3389/fnagi.2021.695808)
Supplement: Supplementary Table 1 — The ACMG-AMP classifications of three genes in AD. [file Table_1.docx]

**Supplementary Table1. The ACMG-AMP classifications of three genes in AD**

| Gene | Variant | Region | Types | TM | PVS1 | PS1 | PS2 | PS3 | PS4 | PM1 | PM2 | PM3 | PM4 | PM5 | PM6 | PP1 | PP2 | PP3 | PP4 | PP5 | Benign criteria | Previous | ACMG | References |
| --- | --- | --- | --- | --- | --- | --- | --- | --- | --- | --- | --- | --- | --- | --- | --- | --- | --- | --- | --- | --- | --- | --- | --- | --- |
| *APP* | A201V | Exon 5 | Missense | No | - | - | - | - | - | - | - | - | - | - | - | - | - | - | - | - | - | Likely benign | VUS | (Sassi et al., 2014a) |
| *APP* | A235V | Exon 6 | Missense | No | - | - | - | - | - | - | - | - | - | - | - | - | - | - | - | - | - | VUS | VUS | (Nicolas et al., 2016a) |
| *APP* | D243N | Exon 6 | Missense | No | - | - | - | - | - | - | - | - | - | - | - | - | - | - | - | - | - | VUS | VUS | (Nicolas et al., 2016a) |
| *APP* | E246K | Exon 6 | Missense | No | - | - | - | - | - | - | - | - | - | - | - | - | - | - | - | - | - | VUS | VUS | (Sala Frigerio et al., 2015) |
| *APP* | E296K | Exon 7 | Missense | No | - | - | - | - | - | - | - | - | - | - | - | - | - | - | - | - | - | VUS | VUS | (Nicolas et al., 2016a) |
| *APP* | P299L | Exon 7 | Missense | No | - | - | - | - | - | - | - | - | - | - | - | - | - | - | - | - | - | VUS | VUS | (Nicolas et al., 2016a) |
| *APP* | D332G | Exon 7 | Missense | No | - | - | - | - | - | - | - | - | - | - | - | - | - | - | - | - | - | VUS | VUS | (Jiang et al., 2019) |
| *APP* | E380K | Exon 9 | Missense | No | - | - | - | - | - | - | - | - | - | - | - | - | - | - | - | - | - | VUS | VUS | (El Bitar et al., 2019) |
| *APP* | R468H | Exon 11 | Missense | No | - | - | - | - | - | - | - | - | - | - | - | - | - | - | - | - | - | Not Pathogenic | VUS | (Schulte et al., 2015) |
| *APP* | A479S | Exon 11 | Missense | No | - | - | - | - | - | - | - | - | - | - | - | - | - | - | - | - | - | VUS | VUS | (Sala Frigerio et al., 2015) |
| *APP* | R486W | Exon 11 | Missense | No | - | - | - | - | - | - | - | - | - | - | - | - | - | - | - | - | - | VUS | VUS | (Wang et al., 2019) |
| *APP* | K496Q | Exon 12 | Missense | No | - | - | - | - | - | - | - | - | - | - | - | - | - | - | - | - | - | VUS | VUS | (Sassi et al., 2014b) |
| *APP* | A500T | Exon 12 | Missense | No | - | - | - | - | - | - | - | - | - | - | - | - | - | - | - | - | - | Not Pathogenic | VUS | (Schulte et al., 2015) |
| *APP* | Y538H | Exon 13 | Missense | No | - | - | - | - | - | - | - | - | - | - | - | - | - | - | - | - | - | VUS | VUS | (Sassi et al., 2014a) |
| *APP* | V562I | Exon 13 | Missense | No | - | - | - | - | - | - | - | - | - | - | - | - | - | - | - | - | - | Likely benign | VUS | (Sassi et al., 2014a) |
| *APP* | E599K | Exon 14 | Missense | No | - | - | - | - | - | - | - | - | - | - | - | - | - | - | - | - | BS1 | Likely benign | VUS | (Sassi et al., 2014a) |
| *APP* | T600M | Exon 14 | Missense | No | - | - | - | - | - | - | - | - | - | - | - | - | - | - | - | - | - | Not Pathogenic | VUS | (Schulte et al., 2015) |
| *APP* | V604M | Exon 14 | Missense | No | - | - | - | - | - | - | - | - | - | - | - | - | - | + | - | - | - | VUS | VUS | (Van Giau et al., 2018) |
| *APP* | P620A | Exon 14 | Missense | No | - | - | - | - | - | - | + | - | - | - | - | - | - | + | - | - | - | VUS | VUS | (Nicolas et al., 2016a) |
| *APP* | P620L | Exon 14 | Missense | No | - | - | - | - | - | - | - | - | - | - | - | - | - | + | - | - | - | Likely benign | VUS | (Sassi et al., 2014b) |
| *APP* | T663M | Exon 16 | Missense | No | - | - | - | - | - | - | - | - | - | - | - | - | - | - | - | - | - | Not Pathogenic | VUS | (Schulte et al., 2015) |
| *APP* | E665D | Exon 16 | Missense | No | - | - | - | - | - | - | - | - | - | - | - | - | - | - | - | - | BS4 | VUS | VUS | (Peacock et al., 1994) |
| *APP* | V669L | Exon 16 | Missense | No | - | - | - | - | - | + | + | - | - | - | - | - | - | - | - | - | - | VUS | VUS | (Bagyinszky et al., 2019) |
| *APP* | KM670/671NL | Exon 16 | Missense | No | - | - | - | + | - | + | + | - | - | - | - | + | - | + | - | + | - | Pathogenic | Pathogenic | (Mullan et al., 1992; Citron et al., 1994) |
| *APP* | A673T | Exon 16 | Missense | No | - | - | - | - | - | - | - | - | - | + | - | - | - | - | - | - | BS3+BP4 | Benign | VUS | (Peacock et al., 1993; Jonsson et al., 2012) |
| *APP* | A673V | Exon 16 | Missense | No | - | - | - | + | - | + | + | - | - | - | - | + | - | - | - | + | - | Pathogenic | Pathogenic | (Di Fede et al., 2009) |
| *APP* | H677R | Exon 16 | Missense | No | - | - | - | - | - | + | + | - | - | - | - | - | - | - | - | - | BS4 | VUS | VUS | (Janssen et al., 2003) |
| *APP* | D678H | Exon 16 | Missense | No | - | - | - | + | - | + | + | - | - | - | - | - | - | - | - | - | - | Pathogenic | Likely Pathogenic | (Chen et al., 2012) |
| *APP* | D678N | Exon 16 | Missense | No | - | - | - | - | - | + | + | - | - | + | - | + | - | - | - | + | - | Pathogenic | Likely Pathogenic | (Wakutani et al., 2004; Chen et al., 2012) |
| *APP* | E682K | Exon 16 | Missense | No | - | - | - | + | - | + | + | - | - | - | - | - | - | + | - | - | - | Pathogenic | Likely Pathogenic | (Zhou et al., 2011) |
| *APP* | K687Q | Exon 16 | Missense | No | - | - | - | - | - | + | + | - | - | + | - | - | - | - | - | - | - | VUS | Likely Pathogenic | (Jiang et al., 2019) |
| *APP* | K687N | Exon 16 | Missense | No | - | - | - | + | - | + | + | - | - | - | - | - | - | - | - | - | - | Pathogenic | Likely Pathogenic | (Kaden et al., 2012) |
| *APP* | A692G | Exon 17 | Missense | No | - | - | - | + | - | + | + | - | - | - | - | + | - | - | - | + | - | Pathogenic | Pathogenic | (Hendriks et al., 1992; Tang et al., 2014) |
| *APP* | E693del | Exon 17 | Indel | No | - | - | - | - | - | + | + | - | + | - | - | + | - | - | - | + | - | Pathogenic | Likely Pathogenic | (Tomiyama et al., 2008; Kutoku et al., 2015) |
| *APP* | E693G | Exon 17 | Missense | No | - | - | - | + | - | + | + | - | - | + | - | + | - | + | - | - | - | Pathogenic | Pathogenic | (Kamino et al., 1992; Nilsberth et al., 2001) |
| *APP* | E693K | Exon 17 | Missense | No | - | - | - | - | - | + | + | - | - | + | - | + | - | - | - | - | - | Pathogenic | Likely Pathogenic | (Bugiani et al., 2010) |
| *APP* | E693Q | Exon 17 | Missense | No | - | - | - | - | - | + | + | - | - | + | - | - | - | + | - | + | - | Pathogenic | Likely Pathogenic | (Levy et al., 1990; Van Broeckhoven et al., 1990) |
| *APP* | D694N | Exon 17 | Missense | No | - | - | - | + | - | + | + | - | - | - | - | + | - | - | - | + | - | Pathogenic | Pathogenic | (Grabowski et al., 2001; Greenberg et al., 2003) |
| *APP* | V695M | Exon 17 | Missense | No | - | - | - | - | - | + | + | - | - | - | - | - | - | - | - | - | - | VUS | VUS | (Gao et al., 2019) |
| *APP* | L705V | Exon 17 | Missense | Yes | - | - | - | - | - | + | + | - | - | - | - | + | - | + | - | - | - | Pathogenic | Likely Pathogenic | (Obici et al., 2005) |
| *APP* | G708G | Exon 17 | Missense | Yes | - | - | - | - | - | - | - | - | - | - | - | - | - | - | - | - | BS1+BP4+BP7 | Not Pathogenic | Likely benign | (Balbín et al., 1992) |
| *APP* | G709S | Exon 17 | Missense | Yes | - | - | - | - | - | + | - | - | - | - | - | - | - | - | - | - | - | Not Pathogenic | VUS | (Schulte et al., 2015) |
| *APP* | A713T | Exon 17 | Missense | Yes | - | - | - | - | - | + | - | - | - | - | - | - | - | - | - | - | - | VUS | VUS | (Carter et al., 1992; Armstrong et al., 2004) |
| *APP* | A713V | Exon 17 | Missense | Yes | - | - | - | - | - | + | - | - | - | - | - | - | - | + | - | - | - | Not Pathogenic | VUS | (Jones et al., 1992) |
| *APP* | T714A | Exon 17 | Missense | Yes | - | - | - | - | - | + | + | - | - | - | - | + | - | - | - | + | - | Pathogenic | Likely Pathogenic | (Pasalar et al., 2002; Lindquist et al., 2008b) |
| *APP* | T714I | Exon 17 | Missense | Yes | - | - | - | + | - | + | + | - | - | + | - | + | - | - | - | + | - | Pathogenic | Pathogenic | (Kumar-Singh et al., 2000; Edwards-Lee et al., 2005) |
| *APP* | V715A | Exon 17 | Missense | Yes | - | - | - | + | - | + | + | - | - | - | - | + | - | - | - | + | - | Pathogenic | Pathogenic | (De Jonghe et al., 2001; Cruts et al., 2003) |
| *APP* | V715M | Exon 17 | Missense | Yes | - | - | - | + | - | + | + | - | - | + | - | - | - | - | - | + | - | Pathogenic | Pathogenic | (Ancolio et al., 1999; Park et al., 2008) |
| *APP* | I716F | Exon 17 | Missense | Yes | - | - | - | + | - | + | + | - | - | + | - | - | - | + | - | + | - | Pathogenic | Pathogenic | (Guardia-Laguarta et al., 2010; Sieczkowski et al., 2015) |
| *APP* | I716M | Exon 17 | Missense | Yes | - | - | - | - | - | + | + | - | - | + | - | - | - | + | - | - | - | Pathogenic | Likely Pathogenic | (Blauwendraat et al., 2016) |
| *APP* | I716T | Exon 17 | Missense | Yes | - | - | - | - | - | + | + | - | - | + | - | - | - | + | - | - | - | Pathogenic | Likely Pathogenic | (Terreni et al., 2002) |
| *APP* | I716V | Exon 17 | Missense | Yes | - | - | - | + | - | + | + | - | - | + | - | - | - | - | - | - | - | Pathogenic | Pathogenic | (Eckman et al., 1997) |
| *APP* | V717F | Exon 17 | Missense | Yes | - | - | - | + | - | + | + | - | - | + | - | + | - | + | - | - | - | Pathogenic | Pathogenic | (Murrell et al., 1991; Finckh et al., 2005) |
| *APP* | V717G | Exon 17 | Missense | Yes | - | - | - | + | - | + | + | - | - | + | - | + | - | + | - | + | - | Pathogenic | Pathogenic | (Fidani et al., 1992; Murrell et al., 2000) |
| *APP* | V717I | Exon 17 | Missense | Yes | - | - | - | + | - | + | + | - | - | + | - | + | - | - | - | + | - | Pathogenic | Pathogenic | (Goate et al., 1991; Herl et al., 2009) |
| *APP* | V717L | Exon 17 | Missense | Yes | - | - | - | + | - | + | + | - | - | + | - | + | - | + | - | + | - | Pathogenic | Pathogenic | (Murrell et al., 2000; Godbolt et al., 2006) |
| *APP* | T719N | Exon 17 | Missense | Yes | - | - | - | + | - | + | + | - | - | + | - | - | - | + | - | - | - | Pathogenic | Pathogenic | (Scahill et al., 2013; Hsu et al., 2018) |
| *APP* | T719P | Exon 17 | Missense | Yes | - | - | - | - | - | + | + | - | - | + | - | - | - | + | - | - | - | Pathogenic | Likely Pathogenic | (Ghidoni et al., 2009) |
| *APP* | M722K | Exon 17 | Missense | Yes | - | - | - | + | - | + | + | - | - | - | - | - | - | + | - | - | - | Pathogenic | Likely Pathogenic | (Wang et al., 2015) |
| *APP* | L723P | Exon 17 | Missense | Yes | - | - | - | + | - | + | + | - | - | - | - | - | - | + | - | + | - | Pathogenic | Pathogenic | (Kwok et al., 2000; Dobricic et al., 2012) |
| *APP* | K724N | Exon 17 | Missense | No | - | - | - | + | - | + | + | - | - | - | - | - | - | - | - | - | - | Pathogenic | Likely Pathogenic | (Theuns et al., 2006) |
| *APP* | H733P | Exon 17 | Missense | No | - | - | - | - | - | + | + | - | - | - | - | - | - | + | - | - | - | Not Pathogenic | VUS | (Guerreiro et al., 2010) |
| *APP* | IVS17 83-88delAAGTAT | Intron 17 | Indel | NA | - | - | - | - | - | - | - | - | - | - | - | + | - | - | - | - | - | Not Pathogenic | VUS | (Kamino et al., 1992) |
| *APP* | c.*18 C>T | 3' UTR | Missense | NA | - | - | - | - | - | - | + | - | - | - | - | - | - | - | - | - | - | VUS | VUS | (Nicolas et al., 2016b) |
| *APP* | c.*331_*332del | 3' UTR | Indel | NA | - | - | - | - | - | - | + | - | - | - | - | + | - | - | - | - | - | Pathogenic | VUS | (Nicolas et al., 2016b) |
| *APP* | c.*372 A>G | 3' UTR | Missense | NA | - | - | - | - | - | - | - | - | - | - | - | - | - | - | - | - | ~~-~~ | Not Pathogenic | VUS | (Nicolas et al., 2016b) |
| *PSEN1* | Q15H | Exon 3 | Missense | No | - | - | - | - | - | - | + | - | - | - | - | - | + | - | - | - | - | VUS | VUS | (Koriath et al., 2020) |
| *PSEN1* | N32N | Exon 4 | Missense | No | - | - | - | - | - | - | - | - | - | - | - | - | + | - | - | - | BP7 | VUS | VUS | (Scacchi et al., 2007) |
| *PSEN1* | R35Q | Exon 4 | Missense | No | - | - | - | - | - | - | - | - | - | - | - | - | + | - | - | - | - | VUS | VUS | (Rogaeva et al., 2001) |
| *PSEN1* | N39Y | Exon 4 | Missense | No | - | - | - | - | - | - | + | - | - | - | - | - | + | - | - | - | - | VUS | VUS | (Koriath et al., 2020) |
| *PSEN1* | D40del (delACG) | Exon 4 | Indel | No | - | - | - | - | - | - | - | - | + | - | - | - | + | - | - | - | BS1 | VUS | VUS | (Nicolas et al., 2016a) |
| *PSEN1* | D40del (delGAC) | Exon 4 | Indel | No | - | - | - | - | - | - | - | - | + | - | - | - | + | - | - | - | BS1 | VUS | VUS | (Nygaard et al., 2014) |
| *PSEN1* | R41S | Exon 4 | Missense | No | - | - | - | - | - | - | + | - | - | - | - | - | + | - | - | - | - | VUS | VUS | (Gatto et al., 2020) |
| *PSEN1* | R42L | Exon 4 | Missense | No | - | - | - | - | - | - | - | - | - | - | - | - | + | - | - | - | - | VUS | VUS | (Koriath et al., 2020) |
| *PSEN1* | P49L | Exon 4 | Missense | No | - | - | - | - | - | - | + | - | - | - | - | - | + | - | - | - | - | VUS | VUS | (Perrone et al., 2020) |
| *PSEN1* | E69D | Exon 4 | Missense | No | - | - | - | - | - | - | + | - | - | - | - | - | + | - | - | - | - | VUS | VUS | (Nicolas et al., 2016a) |
| *PSEN1* | A79V | Exon 4 | Missense | No | - | - | - | + | - | + | - | - | - | - | - | + | + | + | - | + | - | Pathogenic | Pathogenic | (Cruts et al., 1998; Day et al., 2016) |
| *PSEN1* | V82L | Exon 4 | Missense | No | - | - | - | - | - | + | + | - | - | - | - | + | + | + | - | - | - | Pathogenic | Likely Pathogenic | (Campion et al., 1995b; Sun et al., 2017) |
| *PSEN1* | I83_M84del | Exon 4 | Indel | Yes | - | - | - | + | - | + | + | - | + | - | - | - | - | + | - | - | - | Pathogenic | Pathogenic | (Houlden et al., 2000; Steiner et al., 2001) |
| *PSEN1* | I83T | Exon 4 | Missense | Yes | - | - | - | - | - | + | + | - | - | - | - | - | + | + | - | + | - | Pathogenic | Likely Pathogenic | (Achouri-Rassas et al., 2015; Fray et al., 2020) |
| *PSEN1* | M84T | Exon 4 | Missense | Yes | - | - | - | - | - | + | + | - | - | + | - | - | + | - | - | - | - | Pathogenic | Likely Pathogenic | (Lanoiselée et al., 2017) |
| *PSEN1* | M84V | Exon 4 | Missense | Yes | - | - | - | + | - | + | + | - | - | + | - | + | + | - | - | + | - | Pathogenic | Pathogenic | (Hooli et al., 2014; Gallo et al., 2017) |
| *PSEN1* | L85P | Exon 4 | Missense | Yes | - | - | - | + | - | + | + | - | - | - | + | - | + | + | - | + | - | Pathogenic | Pathogenic | (Ataka et al., 2004; López-García et al., 2019) |
| *PSEN1* | P88H | Exon 4 | Missense | Yes | - | - | - | - | - | + | + | - | - | + | - | - | + | + | - | - | - | Pathogenic | Likely Pathogenic | (Lanoiselée et al., 2017) |
| *PSEN1* | P88L | Exon 4 | Missense | Yes | - | - | - | + | - | + | + | - | - | + | - | + | + | + | - | - | - | Pathogenic | Pathogenic | (Liu et al., 2017) |
| *PSEN1* | V89L (G>C) | Exon 4 | Missense | Yes | - | - | - | + | - | + | + | - | - | + | - | - | + | + | - | - | - | Pathogenic | Pathogenic | (Liu et al., 2017) |
| *PSEN1* | V89L (G>T) | Exon 4 | Missense | Yes | - | - | - | + | - | + | + | - | - | + | - | + | + | + | - | - | - | Pathogenic | Pathogenic | (Lleó et al., 2002a; Queralt et al., 2002) |
| *PSEN1* | C92S | Exon 4 | Missense | Yes | - | - | - | + | - | + | + | - | - | - | - | - | + | + | - | - | - | Pathogenic | Pathogenic | (Lewis et al., 2000; Tedde et al., 2003) |
| *PSEN1* | V94M | Exon 4 | Missense | Yes | - | - | - | - | - | + | - | - | - | - | - | - | + | - | - | - | - | VUS | VUS | (Arango et al., 2001) |
| *PSEN1* | V96F | Exon 4 | Missense | Yes | - | - | - | + | - | + | + | - | - | - | - | + | + | + | - | + | - | Pathogenic | Pathogenic | (Kamino et al., 1996) |
| *PSEN1* | V97L | Exon 4 | Missense | Yes | - | - | - | + | - | + | + | - | - | - | - | + | + | + | - | - | - | Pathogenic | Pathogenic | (Jia et al., 2005; Fang et al., 2006) |
| *PSEN1* | T99A | Exon 4 | Missense | Yes | - | - | - | + | - | + | + | - | - | - | - | - | + | - | - | - | - | VUS | Likely Pathogenic | (Ikeda et al., 2013) |
| *PSEN1* | V103G | Exon 4 | Missense | No | - | - | - | - | - | + | + | - | - | - | - | - | + | - | - | - | - | VUS | VUS | (Gao et al., 2019) |
| *PSEN1* | F105C | Exon 4 | Missense | No | - | - | - | - | - | + | + | - | - | + | - | + | + | + | - | + | - | Pathogenic | Likely Pathogenic | (Deng et al., 2014) |
| *PSEN1* | F105I | Exon 4 | Missense | No | - | - | - | + | - | + | + | - | - | + | - | - | + | - | - | - | - | Pathogenic | Pathogenic | (Raux et al., 2005) |
| *PSEN1* | F105L | Exon 4 | Missense | No | - | - | - | - | - | + | + | - | - | + | - | - | + | - | - | - | - | Pathogenic | Likely Pathogenic | (Finckh et al., 2000b) |
| *PSEN1* | F105V | Exon 4 | Missense | No | - | - | - | - | - | + | + | - | - | + | - | - | + | - | - | - | - | Pathogenic | Likely Pathogenic | (Gómez-Tortosa et al., 2010) |
| *PSEN1* | R108Q | Exon 4 | Missense | No | - | - | - | - | - | - | - | - | - | - | - | - | + | - | - | - | - | VUS | VUS | (Dobricic et al., 2012) |
| *PSEN1* | G111V | Exon 4 | Missense | No | - | - | - | + | - | + | + | - | - | + | - | - | + | - | - | - | - | Pathogenic | Pathogenic | (Qiu et al., 2020) |
| *PSEN1* | G111W | Exon 4 | Missense | No | - | - | + | - | - | + | + | - | - | + | - | - | + | + | - | - | - | Pathogenic | Pathogenic | (Lanoiselée et al., 2017) |
| *PSEN1* | L113_I114insT | Intron 4 | Indel | No | - | - | - | + | - | + | + | - | - | - | - | + | - | - | - | + | - | Pathogenic | Pathogenic | (Tysoe et al., 1998; Szaruga et al., 2015) |
| *PSEN1* | L113P | Exon 4 | Missense | No | - | - | - | - | - | + | + | - | - | + | - | + | + | + | - | + | - | Pathogenic | Likely Pathogenic | (Raux et al., 2000b) |
| *PSEN1* | L113Q | Exon 4 | Missense | No | - | - | - | + | - | + | + | - | - | + | - | - | + | + | - | - | - | Pathogenic | Pathogenic | (Finckh et al., 2005; Sun et al., 2017) |
| *PSEN1* | Y115C | Exon 5 | Missense | No | - | - | - | + | - | + | + | - | - | + | - | + | + | + | - | + | - | Pathogenic | Pathogenic | (De Jonghe et al., 1999) |
| *PSEN1* | Y115D | Exon 5 | Missense | No | - | - | - | - | - | + | + | - | - | + | - | - | + | - | - | - | - | Pathogenic | Likely Pathogenic | - |
| *PSEN1* | Y115H | Exon 5 | Missense | No | - | - | + | + | - | + | + | - | - | + | - | + | + | + | - | + | - | Pathogenic | Pathogenic | (Campion et al., 1999) |
| *PSEN1* | T116_P117delinsST | Exon 5 | Indel | No | - | - | - | - | - | - | + | - | + | - | - | - | - | - | - | - | - | Pathogenic | Pathogenic | (Blanco et al., 2019) |
| *PSEN1* | T116S; P117T | Exon 5 | Missense | No | - | - | - | - | - | + | + | - | - | + | - | - | + | + | - | - | - | Pathogenic | Likely Pathogenic | (Blanco et al., 2019) |
| *PSEN1* | T116I | Exon 5 | Missense | No | - | - | - | - | - | + | + | - | - | + | - | + | + | + | - | + | - | Pathogenic | Likely Pathogenic | (An et al., 2016b) |
| *PSEN1* | T116N | Exon 5 | Missense | No | - | - | - | + | - | + | + | - | - | + | - | + | + | + | - | + | - | Pathogenic | Pathogenic | (Romero et al., 1999) |
| *PSEN1* | T116R | Exon 5 | Missense | No | - | - | - | - | - | + | + | - | - | + | - | - | + | - | - | - | - | Pathogenic | Likely Pathogenic | (Mann et al., 2001) |
| *PSEN1* | P117A | Exon 5 | Missense | No | - | - | - | + | - | + | + | - | - | + | - | + | + | + | - | + | - | Pathogenic | Pathogenic | (Anheim et al., 2007) |
| *PSEN1* | P117L | Exon 5 | Missense | No | - | - | - | + | - | + | + | - | - | + | - | + | + | + | - | + | - | Pathogenic | Pathogenic | (Alberici et al., 2007) |
| *PSEN1* | P117Q | Exon 5 | Missense | No | - | - | + | - | - | + | + | - | - | + | - | - | + | + | - | - | - | Pathogenic | Pathogenic | (Lanoiselée et al., 2017) |
| *PSEN1* | P117R | Exon 5 | Missense | No | - | - | - | + | - | + | + | - | - | + | - | - | + | + | - | + | - | Pathogenic | Pathogenic | (Bialopiotrowicz et al., 2012) |
| *PSEN1* | P117S | Exon 5 | Missense | No | - | - | - | + | - | + | + | - | - | + | - | + | + | + | - | - | - | Pathogenic | Pathogenic | (Dowjat et al., 2004) |
| *PSEN1* | T119I | Exon 5 | Missense | No | - | - | - | - | - | + | + | - | - | - | - | - | + | - | - | + | - | Pathogenic | Likely Pathogenic | (Itzcovich et al., 2020) |
| *PSEN1* | E120D (A>C) | Exon 5 | Missense | No | - | - | - | + | - | + | + | - | - | + | - | - | + | - | - | + | - | Pathogenic | Pathogenic | (Poorkaj et al., 1998) |
| *PSEN1* | E120D (A>T) | Exon 5 | Missense | No | - | - | - | + | - | + | + | - | - | + | - | - | + | - | - | - | - | Pathogenic | Pathogenic | (Reznik-Wolf et al., 1996) |
| *PSEN1* | E120G | Exon 5 | Missense | No | - | - | - | + | - | + | + | - | - | + | - | + | + | + | - | - | - | Pathogenic | Pathogenic | (Sarroca et al., 2016) |
| *PSEN1* | E120K | Exon 5 | Missense | No | - | - | - | + | - | + | + | - | - | + | - | - | + | - | - | + | - | Pathogenic | Pathogenic | (Hutton et al., 1996) |
| *PSEN1* | T122A | Exon 5 | Missense | No | - | - | - | - | - | + | + | - | - | - | - | - | + | + | - | - | - | Pathogenic | Likely Pathogenic | (Koriath et al., 2020) |
| *PSEN1* | E123K | Exon 5 | Missense | No | - | - | - | + | - | + | + | - | - | - | - | - | + | - | - | - | - | Pathogenic | Likely Pathogenic | (Yasuda et al., 1999) |
| *PSEN1* | Q127_R128del(CAGA);InsG(G) | Exon 5 | Indel | No | - | - | - | - | - | + | + | - | + | - | - | - | - | - | - | - | - | Pathogenic | Likely Pathogenic | (Hsu et al., 2020) |
| *PSEN1* | H131R | Exon 5 | Missense | No | - | - | - | + | - | + | + | - | - | - | - | - | + | - | - | - | - | VUS | Likely Pathogenic | (Hsu et al., 2020) |
| *PSEN1* | S132A | Exon 5 | Missense | No | - | - | - | - | - | + | - | - | - | - | - | - | + | - | - | + | - | Pathogenic | VUS | (Ryan et al., 2016) |
| *PSEN1* | L134R | Exon 5 | Missense | Yes | - | - | - | + | - | + | + | - | - | - | - | - | + | + | - | - | - | Pathogenic | Pathogenic | (Lohmann et al., 2012) |
| *PSEN1* | N135D | Exon 5 | Missense | Yes | - | - | - | + | - | + | + | - | - | + | - | + | + | + | - | - | - | Pathogenic | Pathogenic | (Crook et al., 1997) |
| *PSEN1* | N135S | Exon 5 | Missense | Yes | - | - | - | - | - | + | + | - | - | + | - | - | + | + | - | + | - | Pathogenic | Likely Pathogenic | (Rudzinski et al., 2008) |
| *PSEN1* | N135Y | Exon 5 | Missense | Yes | - | - | - | + | - | + | + | - | - | + | - | - | + | + | - | - | - | Pathogenic | Pathogenic | (Natelson Love et al., 2017) |
| *PSEN1* | A136G | Exon 5 | Missense | Yes | - | - | - | - | - | + | + | - | - | - | - | - | + | - | - | - | - | Pathogenic | VUS | (Fang and Jia, 2008) |
| *PSEN1* | A137T | Exon 5 | Missense | Yes | - | - | - | - | - | + | + | - | - | - | - | - | + | - | - | - | - | Pathogenic | VUS | (Koriath et al., 2020) |
| *PSEN1* | M139I (G>A) | Exon 5 | Missense | Yes | - | - | - | + | - | + | + | - | - | + | - | + | + | - | - | + | - | Pathogenic | Pathogenic | (Jiang et al., 2019) |
| *PSEN1* | M139I (G>C) | Exon 5 | Missense | Yes | - | - | - | + | - | + | + | - | - | + | - | + | + | - | - | - | - | Pathogenic | Pathogenic | (Kim et al., 2010) |
| *PSEN1* | M139K | Exon 5 | Missense | Yes | - | - | - | - | - | + | + | - | - | + | + | - | + | + | - | + | - | Pathogenic | Likely Pathogenic | (Dumanchin et al., 1998) |
| *PSEN1* | M139L | Exon 5 | Missense | Yes | - | - | - | + | - | + | + | - | - | + | - | + | + | - | - | - | - | Pathogenic | Pathogenic | (Qiu et al., 2019) |
| *PSEN1* | M139T | Exon 5 | Missense | Yes | - | - | - | + | - | + | + | - | - | + | - | - | + | + | - | + | - | Pathogenic | Pathogenic | (Murayama et al., 1999) |
| *PSEN1* | M139V | Exon 5 | Missense | Yes | - | - | - | + | - | + | + | - | - | + | - | - | + | - | - | + | - | Pathogenic | Pathogenic | (1995) |
| *PSEN1* | V142F | Exon 5 | Missense | Yes | - | - | - | - | - | + | + | - | - | + | - | + | + | + | - | - | - | Pathogenic | Likely Pathogenic | (Wang et al., 2018) |
| *PSEN1* | V142I | Exon 5 | Missense | Yes | - | - | - | - | - | + | + | - | - | + | - | - | + | - | - | - | - | Pathogenic | Likely Pathogenic | (Koriath et al., 2020) |
| *PSEN1* | I143F | Exon 5 | Missense | Yes | - | - | - | - | - | + | + | - | - | + | - | - | + | + | - | + | - | Pathogenic | Likely Pathogenic | (Rossor et al., 1996) |
| *PSEN1* | I143M | Exon 5 | Missense | Yes | - | - | - | - | - | + | + | - | - | + | - | - | + | - | - | - | - | Pathogenic | Likely Pathogenic | (Heckmann et al., 2004) |
| *PSEN1* | I143N | Exon 5 | Missense | Yes | - | - | - | - | - | + | + | - | - | + | - | - | + | + | - | - | - | Pathogenic | Likely Pathogenic | (Finckh et al., 2000b) |
| *PSEN1* | I143T | Exon 5 | Missense | Yes | - | - | - | + | - | + | + | - | - | + | - | + | + | + | - | + | - | Pathogenic | Pathogenic | (Cruts et al., 1995) |
| *PSEN1* | I143V | Exon 5 | Missense | Yes | - | - | - | + | - | + | + | - | - | + | - | - | + | - | - | - | - | Pathogenic | Pathogenic | (Gallo et al., 2011; Sun et al., 2017) |
| *PSEN1* | M146I (G>T) | Exon 5 | Missense | Yes | - | - | - | + | - | + | + | - | - | + | - | - | + | + | - | - | - | Pathogenic | Pathogenic | (Arber et al., 2020) |
| *PSEN1* | M146I (G>C) | Exon 5 | Missense | Yes | - | - | - | + | - | + | + | - | - | + | - | - | + | + | - | - | - | Pathogenic | Pathogenic | (Arber et al., 2020) |
| *PSEN1* | M146I (G>A) | Exon 5 | Missense | Yes | - | - | - | + | - | + | + | - | - | + | - | + | + | + | - | + | - | Pathogenic | Pathogenic | (Jørgensen et al., 1996) |
| *PSEN1* | M146L (A>C) | Exon 5 | Missense | Yes | - | - | - | + | - | + | + | - | - | + | - | + | + | + | - | + | - | Pathogenic | Pathogenic | (Sherrington et al., 1995) |
| *PSEN1* | M146L (A>T) | Exon 5 | Missense | Yes | - | - | - | + | - | + | + | - | - | + | - | + | + | + | - | - | - | Pathogenic | Pathogenic | (Mangone et al., 1995) |
| *PSEN1* | M146V | Exon 5 | Missense | Yes | - | - | - | + | - | + | + | - | - | + | - | + | + | + | - | + | - | Pathogenic | Pathogenic | (Hsu et al., 2020) |
| *PSEN1* | T147I | Exon 5 | Missense | Yes | - | - | - | + | - | + | + | - | - | + | - | + | + | + | - | + | - | Pathogenic | Pathogenic | (Campion et al., 1999) |
| *PSEN1* | T147P | Exon 5 | Missense | Yes | - | - | - | - | - | + | + | - | - | + | - | - | + | + | - | - | - | Pathogenic | Likely Pathogenic | (Testi et al., 2014) |
| *PSEN1* | L150P | Exon 5 | Missense | Yes | - | - | - | - | - | + | + | - | - | - | - | - | + | + | - | - | - | Pathogenic | Likely Pathogenic | (Guerreiro et al., 2010) |
| *PSEN1* | L153V | Exon 5 | Missense | Yes | - | - | - | - | - | + | + | - | - | - | - | + | + | + | - | + | - | Pathogenic | Likely Pathogenic | (Raux et al., 2000a) |
| *PSEN1* | Y154C | Exon 5 | Missense | Yes | - | - | - | - | - | + | + | - | - | - | - | - | + | + | - | - | - | Pathogenic | Likely Pathogenic | (Janssen et al., 2003) |
| *PSEN1* | Y154N | Exon 5 | Missense | Yes | - | - | - | - | - | + | + | - | - | + | - | + | + | + | - | - | - | Pathogenic | Likely Pathogenic | (Hattori et al., 2004) |
| *PSEN1* | Y156F; Y156_R157insIY | Exon 5 | Indel | No | - | - | - | - | - | + | + | - | + | - | - | - | - | - | - | + | - | Pathogenic | Likely Pathogenic | (Moretti et al., 2004) |
| *PSEN1* | R157S | Exon 5 | Missense | No | - | - | - | - | - | + | - | - | - | - | - | - | + | + | - | - | - | VUS | VUS | (Jiang et al., 2019) |
| *PSEN1* | Y159C | Exon 5 | Missense | No | - | - | - | - | - | + | - | - | - | + | - | - | + | + | - | - | - | Pathogenic | Likely Pathogenic | (Kim et al., 2020) |
| *PSEN1* | Y159F | Exon 5 | Missense | No | - | - | - | - | - | + | - | - | - | + | - | + | + | + | - | - | - | Pathogenic | Likely Pathogenic | (Kerchner and Holbrook, 2012) |
| *PSEN1* | H163P | Exon 5 | Missense | Yes | - | - | - | + | - | + | + | - | - | + | - | - | + | + | - | - | - | VUS | Pathogenic | (Kim et al., 2012) |
| *PSEN1* | H163R | Exon 5 | Missense | Yes | - | - | - | - | - | + | + | - | - | + | - | + | + | - | - | + | - | Pathogenic | Likely Pathogenic | (Murayama et al., 1999) |
| *PSEN1* | H163Y | Exon 5 | Missense | Yes | - | - | - | + | - | + | + | - | - | + | - | - | + | - | - | + | - | Pathogenic | Pathogenic | (Thordardottir et al., 2017; Thordardottir et al., 2018) |
| *PSEN1* | A164V | Exon 6 | Missense | Yes | - | - | - | - | - | + | + | - | - | - | - | - | + | + | - | - | - | Pathogenic | Likely Pathogenic | (Roeber et al., 2015) |
| *PSEN1* | W165C (G>C) | Exon 6 | Missense | Yes | - | - | - | - | - | + | + | - | - | + | - | - | + | + | - | - | - | Pathogenic | Likely Pathogenic | (Campion et al., 1999) |
| *PSEN1* | W165C (G>T) | Exon 6 | Missense | Yes | - | - | - | - | - | + | + | - | - | + | - | - | + | + | - | + | - | Pathogenic | Likely Pathogenic | (Van Giau et al., 2019) |
| *PSEN1* | W165G | Exon 6 | Missense | Yes | - | - | - | + | - | + | + | - | - | + | - | - | + | + | - | - | - | Pathogenic | Pathogenic | (Sun et al., 2017) |
| *PSEN1* | L166H | Exon 6 | Missense | Yes | - | - | - | - | - | + | + | - | - | + | - | - | + | + | - | - | - | Pathogenic | Likely Pathogenic | (Pantieri et al., 2005) |
| *PSEN1* | L166P | Exon 6 | Missense | Yes | - | - | - | + | - | + | + | - | - | + | - | - | + | + | - | + | - | Pathogenic | Pathogenic | (Moehlmann et al., 2002) |
| *PSEN1* | L166R | Exon 6 | Missense | Yes | - | - | - | - | - | + | + | - | - | + | - | + | + | + | - | - | - | Pathogenic | Likely Pathogenic | (Ezquerra et al., 2000) |
| *PSEN1* | L166V | Exon 6 | Missense | Yes | - | - | - | - | - | + | + | - | - | + | + | - | + | + | - | - | - | Pathogenic | Likely Pathogenic | (Sassi et al., 2014b) |
| *PSEN1* | L166del | Exon 6 | Indel | Yes | - | - | - | - | - | + | + | - | + | - | - | - | + | + | - | - | - | Pathogenic | Likely Pathogenic | (Knight et al., 2007) |
| *PSEN1* | I167del (TTAdel) | Exon 6 | Indel | Yes | - | - | - | + | - | + | + | - | + | - | - | + | + | + | - | - | - | Pathogenic | Pathogenic | (Jiao et al., 2014) |
| *PSEN1* | I167del (TATdel) | Exon 6 | Missense | Yes | - | - | - | + | - | + | + | - | + | - | - | - | + | + | - | - | - | Pathogenic | Pathogenic | (Janssen et al., 2003) |
| *PSEN1* | I168T | Exon 6 | Missense | Yes | - | - | - | + | - | + | + | - | - | - | - | - | + | - | - | - | - | Pathogenic | Likely Pathogenic | (Sassi et al., 2014a) |
| *PSEN1* | S169del | Exon 6 | Indel | Yes | - | - | - | + | - | + | + | - | + | - | - | - | + | - | - | - | - | Pathogenic | Pathogenic | (Guo et al., 2010) |
| *PSEN1* | S169L | Exon 6 | Missense | Yes | - | - | - | - | - | + | + | - | - | + | - | - | + | + | - | + | - | Pathogenic | Likely Pathogenic | (Taddei et al., 1998) |
| *PSEN1* | S169P | Exon 6 | Missense | Yes | - | - | - | + | - | + | + | - | - | + | - | - | + | + | - | - | - | Pathogenic | Pathogenic | (Ezquerra et al., 1999) |
| *PSEN1* | S170F | Exon 6 | Missense | Yes | - | - | - | + | - | + | + | - | - | + | + | + | + | + | - | - | - | Pathogenic | Pathogenic | (Snider et al., 2005; Li et al., 2020) |
| *PSEN1* | S170P | Exon 6 | Missense | Yes | - | - | - | - | - | + | + | - | - | + | + | - | + | + | - | + | - | Pathogenic | Likely Pathogenic | (Carecchio et al., 2017) |
| *PSEN1* | L171P | Exon 6 | Missense | Yes | - | - | - | - | - | + | + | - | - | - | - | - | + | + | - | + | - | Pathogenic | Likely Pathogenic | (Janssen et al., 2003) |
| *PSEN1* | L173F (G>T) | Exon 6 | Missense | Yes | - | + | - | - | - | + | + | - | - | - | - | - | + | + | - | - | - | Pathogenic | Pathogenic | (Jin et al., 2012) |
| *PSEN1* | L173F (G>C) | Exon 6 | Missense | Yes | - | + | - | + | - | + | + | - | - | - | - | + | + | + | - | - | - | Pathogenic | Pathogenic | (Kasuga et al., 2009) |
| *PSEN1* | L173S | Exon 6 | Missense | Yes | - | - | - | - | - | + | + | - | - | + | - | - | + | + | - | - | - | Pathogenic | Likely Pathogenic | (Wang et al., 2019) |
| *PSEN1* | L173W | Exon 6 | Missense | Yes | - | - | - | + | - | + | + | - | - | + | - | - | + | + | - | + | - | Pathogenic | Pathogenic | (Campion et al., 1999) |
| *PSEN1* | L174del | Exon 6 | Indel | Yes | - | - | - | - | - | + | + | - | + | - | - | - | + | + | - | - | - | Pathogenic | Likely Pathogenic | (Tiedt et al., 2013) |
| *PSEN1* | L174M | Exon 6 | Missense | Yes | - | - | - | + | - | + | + | - | - | + | - | + | + | + | - | + | - | Pathogenic | Pathogenic | (Bertoli Avella et al., 2002) |
| *PSEN1* | L174R | Exon 6 | Missense | Yes | - | - | - | - | - | + | + | - | - | + | - | - | + | + | - | - | - | Pathogenic | Likely Pathogenic | (Klünemann et al., 2004) |
| *PSEN1* | F175del | Exon 6 | Indel | Yes | - | - | - | + | - | + | + | - | + | - | - | - | + | + | - | - | - | Pathogenic | Pathogenic | (Vöglein et al., 2019) |
| *PSEN1* | F175S | Exon 6 | Missense | Yes | - | - | - | - | - | + | + | - | - | - | - | - | + | - | - | - | - | VUS | VUS | (Colacicco et al., 2002) |
| *PSEN1* | F176L | Exon 6 | Missense | Yes | - | - | - | + | - | + | + | - | - | - | - | - | + | - | - | - | - | VUS | Likely Pathogenic | (Müller et al., 2013) |
| *PSEN1* | F177L | Exon 6 | Missense | Yes | - | - | - | + | - | + | + | - | - | + | - | - | + | - | - | + | - | Pathogenic | Pathogenic | (Rogaeva et al., 2001) |
| *PSEN1* | F177S | Exon 6 | Missense | Yes | - | - | - | - | - | + | + | - | - | + | - | - | + | - | - | + | - | Pathogenic | Likely Pathogenic | (Hausner et al., 2014) |
| *PSEN1* | S178P | Exon 6 | Missense | Yes | - | - | - | + | - | + | + | - | - | - | - | + | + | + | - | - | - | Pathogenic | Pathogenic | (Rogaeva et al., 2001) |
| *PSEN1* | I180N | Exon 6 | Missense | Yes | - | - | - | - | - | + | + | - | - | - | + | - | + | + | - | - | - | Pathogenic | Likely Pathogenic | (Lanoiselée et al., 2017) |
| *PSEN1* | G183V | Exon 6 | Missense | No | - | - | - | + | - | + | + | - | - | - | - | - | + | - | - | - | - | Pathogenic | Likely Pathogenic | (Dermaut et al., 2004) |
| *PSEN1* | E184D | Exon 7 | Missense | No | - | - | - | + | - | + | + | - | - | + | - | + | + | + | - | + | - | Pathogenic | Pathogenic | (Yasuda et al., 1997) |
| *PSEN1* | E184G | Exon 7 | Missense | No | - | - | - | + | - | + | + | - | - | + | - | + | + | + | - | + | - | Pathogenic | Pathogenic | (Zarea et al., 2016) |
| *PSEN1* | V191A | Exon 7 | Missense | No | - | - | - | - | - | + | + | - | - | - | - | - | + | - | - | - | - | Not Pathogenic | VUS | (Hsu et al., 2020) |
| *PSEN1* | I202F | Exon 7 | Missense | Yes | - | - | - | + | - | + | - | - | - | - | - | + | + | + | - | + | - | Pathogenic | Pathogenic | (Church et al., 2011) |
| *PSEN1* | W203C | Exon 7 | Missense | Yes | - | - | - | - | - | + | + | - | - | - | - | - | + | + | - | - | - | VUS | Likely Pathogenic | (Couthouis et al., 2014) |
| *PSEN1* | F205_G206del;insC | Exon 7 | Indel | Yes | - | - | + | - | - | + | + | - | + | - | - | - | + | + | - | - | - | Pathogenic | Pathogenic | (Lanoiselée et al., 2017) |
| *PSEN1* | G206A | Exon 7 | Missense | Yes | - | - | - | + | - | + | - | - | - | + | - | - | + | + | - | + | ~~-~~ | Pathogenic | Pathogenic | (Rogaeva et al., 2001) |
| *PSEN1* | G206D | Exon 7 | Missense | Yes | - | - | - | + | - | + | + | - | - | + | - | + | + | + | - | + | - | Pathogenic | Pathogenic | (Chen et al., 2015) |
| *PSEN1* | G206S | Exon 7 | Missense | Yes | - | - | + | + | - | + | + | - | - | + | - | - | + | - | - | + | - | Pathogenic | Pathogenic | (Liu et al., 2019) |
| *PSEN1* | G206V | Exon 7 | Missense | Yes | - | - | - | - | - | + | + | - | - | + | + | - | + | + | - | + | - | Pathogenic | Likely Pathogenic | (Goldman et al., 2002) |
| *PSEN1* | G209A | Exon 7 | Missense | Yes | - | - | - | - | - | + | + | - | - | + | - | - | + | + | - | - | - | Pathogenic | Likely Pathogenic | (An et al., 2016a) |
| *PSEN1* | G209E | Exon 7 | Missense | Yes | - | - | - | - | - | + | + | - | - | + | - | - | + | + | - | + | - | Pathogenic | Likely Pathogenic | (Rogaeva et al., 2001) |
| *PSEN1* | G209R | Exon 7 | Missense | Yes | - | - | - | + | - | + | + | - | - | + | - | + | + | + | - | - | - | Pathogenic | Pathogenic | (Sugiyama et al., 1999) |
| *PSEN1* | G209V | Exon 7 | Missense | Yes | - | - | - | + | - | + | + | - | - | + | - | + | + | + | - | - | - | Pathogenic | Pathogenic | (Poorkaj et al., 1998) |
| *PSEN1* | M210R | Exon 7 | Missense | Yes | - | - | - | - | - | + | + | - | - | - | - | - | + | + | - | - | - | Pathogenic | Likely Pathogenic | (Lanoiselée et al., 2017) |
| *PSEN1* | S212Y | Exon 7 | Missense | Yes | - | - | - | + | - | + | + | - | - | - | - | + | + | + | - | - | - | Pathogenic | Pathogenic | (Ringman et al., 2011) |
| *PSEN1* | I213F | Exon 7 | Missense | Yes | - | - | - | + | - | + | + | - | - | + | - | - | + | + | - | - | - | Pathogenic | Pathogenic | (Bialopiotrowicz et al., 2012) |
| *PSEN1* | I213L | Exon 7 | Missense | Yes | - | - | - | + | - | + | + | - | - | + | - | + | + | + | - | + | - | Pathogenic | Pathogenic | (Rogaeva et al., 2001) |
| *PSEN1* | I213T | Exon 7 | Missense | Yes | - | - | - | + | - | + | + | - | - | + | - | - | + | + | - | + | - | Pathogenic | Pathogenic | (Shimojo et al., 2008) |
| *PSEN1* | H214D | Exon 7 | Missense | No | - | - | - | + | - | + | + | - | - | + | - | - | + | + | - | - | - | Pathogenic | Pathogenic | (Sun et al., 2017) |
| *PSEN1* | H214N | Exon 7 | Missense | No | - | - | - | - | - | + | + | - | - | + | - | - | + | + | - | - | - | Pathogenic | Likely Pathogenic | (Piccoli et al., 2016) |
| *PSEN1* | H214R | Exon 7 | Missense | No | - | - | - | - | - | + | + | - | - | + | - | - | + | + | - | - | - | Pathogenic | Likely Pathogenic | (Li et al., 2019) |
| *PSEN1* | H214Y | Exon 7 | Missense | No | - | - | - | - | - | + | + | - | - | + | - | - | + | + | - | + | - | Pathogenic | Likely Pathogenic | (Finckh et al., 2000b) |
| *PSEN1* | G217D | Exon 7 | Missense | No | - | - | - | - | - | + | + | - | - | + | - | - | + | + | - | - | - | Pathogenic | Likely Pathogenic | (Takao et al., 2002) |
| *PSEN1* | G217R | Exon 7 | Missense | No | - | - | - | + | - | + | + | - | - | + | - | - | + | + | - | - | - | Pathogenic | Pathogenic | (Norton et al., 2009) |
| PSEN1 | L219F | Exon 7 | Missense | Yes | - | - | - | + | - | + | + | - | - | + | - | - | + | + | - | - | - | Pathogenic | Pathogenic | (Terreni et al., 2000) |
| *PSEN1* | L219P | Exon 7 | Missense | Yes | - | - | - | - | - | + | + | - | - | + | - | - | + | + | - | - | - | Pathogenic | Likely Pathogenic | (Smith et al., 1999) |
| *PSEN1* | L219R | Exon 7 | Missense | Yes | - | - | - | - | - | + | + | - | - | + | - | - | + | + | - | - | - | VUS | Likely Pathogenic | (Ikeda et al., 2013) |
| *PSEN1* | R220P | Exon 7 | Missense | Yes | - | - | - | - | - | + | + | - | - | - | - | - | + | - | - | - | - | Pathogenic | VUS | (Piccoli et al., 2016) |
| *PSEN1* | Q222H | Exon 7 | Missense | Yes | - | - | - | - | - | + | + | - | - | + | - | - | + | + | - | - | - | Pathogenic | Likely Pathogenic | (Miklossy et al., 2003) |
| *PSEN1* | Q222P | Exon 7 | Missense | Yes | - | - | - | - | - | + | + | - | - | + | - | - | + | + | - | + | - | Pathogenic | Likely Pathogenic | (Scahill et al., 2013) |
| *PSEN1* | Q222R | Exon 7 | Missense | Yes | - | - | - | + | - | + | + | - | - | + | - | + | + | + | - | - | - | Pathogenic | Pathogenic | (Rogaeva et al., 2001) |
| *PSEN1* | Q223R | Exon 7 | Missense | Yes | - | - | - | - | - | + | + | - | - | - | - | - | + | + | - | + | - | Pathogenic | Likely Pathogenic | (Uttner et al., 2010) |
| *PSEN1* | L226F | Exon 7 | Missense | Yes | - | - | - | + | - | + | + | - | - | + | + | - | + | + | - | + | - | Pathogenic | Pathogenic | (Bagyinszky et al., 2016) |
| *PSEN1* | L226R | Exon 7 | Missense | Yes | - | - | - | - | - | + | + | - | - | + | - | - | + | + | - | - | - | Pathogenic | Likely Pathogenic | (Coleman et al., 2004) |
| *PSEN1* | I227V | Exon 7 | Missense | Yes | - | - | - | - | - | + | - | - | - | - | - | - | + | - | - | - | ~~-~~ | Pathogenic | VUS | (Koriath et al., 2020) |
| *PSEN1* | I229F | Exon 7 | Missense | Yes | - | - | - | + | - | + | + | - | - | - | - | - | + | + | - | - | - | Pathogenic | Pathogenic | (Sassi et al., 2014b) |
| *PSEN1* | S230I | Exon 7 | Missense | Yes | - | - | - | + | - | + | + | - | - | + | - | - | + | + | - | - | - | Pathogenic | Pathogenic | (Wallon et al., 2012) |
| *PSEN1* | S230N | Exon 7 | Missense | Yes | - | - | - | - | - | + | + | - | - | + | - | + | + | + | - | - | - | Pathogenic | Likely Pathogenic | (Ringman et al., 2017) |
| *PSEN1* | S230R | Exon 7 | Missense | Yes | - | - | - | - | - | + | + | - | - | + | - | - | + | + | - | - | - | Pathogenic | Likely Pathogenic | (Sassi et al., 2014b) |
| *PSEN1* | A231P | Exon 7 | Missense | Yes | - | - | - | - | - | + | + | - | - | + | - | - | + | + | - | - | - | Pathogenic | Likely Pathogenic | (Nicolas et al., 2016a) |
| *PSEN1* | A231T | Exon 7 | Missense | Yes | - | - | - | - | - | + | + | - | - | + | - | - | + | + | - | - | - | Pathogenic | Likely Pathogenic | (Campion et al., 1995b) |
| *PSEN1* | A231V | Exon 7 | Missense | Yes | - | - | - | - | - | + | + | - | - | + | - | - | + | + | - | - | - | Pathogenic | Likely Pathogenic | (Cruts et al., 1998) |
| *PSEN1* | L232P | Exon 7 | Missense | Yes | - | - | - | - | - | + | + | - | - | - | - | - | + | + | - | - | - | Pathogenic | Likely Pathogenic | (Park et al., 2017) |
| *PSEN1* | M233I (G>A) | Exon 7 | Missense | Yes | - | - | - | - | - | + | + | - | - | + | - | + | + | - | - | - | - | Pathogenic | Likely Pathogenic | (Wallon et al., 2012) |
| *PSEN1* | M233I (G>C) | Exon 7 | Missense | Yes | - | - | + | - | - | + | + | - | - | + | - | + | + | - | - | - | - | Pathogenic | Pathogenic | (Portet et al., 2003) |
| *PSEN1* | M233L (A>C) | Exon 7 | Missense | Yes | - | - | - | + | - | + | + | - | - | + | - | + | + | + | - | + | - | Pathogenic | Pathogenic | (Jiang et al., 2015) |
| *PSEN1* | M233L (A>T) | Exon 7 | Missense | Yes | - | - | - | + | - | + | + | - | - | + | - | - | + | + | - | + | - | Pathogenic | Pathogenic | (Mendez and McMurtray, 2006) |
| *PSEN1* | M233T | Exon 7 | Missense | Yes | - | - | - | + | - | + | + | - | - | + | - | + | + | + | - | + | - | Pathogenic | Pathogenic | (Kwok et al., 1997) |
| *PSEN1* | M233V | Exon 7 | Missense | Yes | - | - | - | + | - | + | + | - | - | + | + | - | + | + | - | + | - | Pathogenic | Pathogenic | (Houlden et al., 2001) |
| *PSEN1* | L235P | Exon 7 | Missense | Yes | - | - | - | + | - | + | + | - | - | + | - | + | + | + | - | + | - | Pathogenic | Pathogenic | (Campion et al., 1996) |
| *PSEN1* | L235R | Exon 7 | Missense | Yes | - | - | - | + | - | + | + | - | - | + | - | - | + | + | - | + | - | Pathogenic | Pathogenic | (Antonell et al., 2011) |
| *PSEN1* | L235V | Exon 7 | Missense | Yes | - | - | - | - | - | + | + | - | - | + | - | + | + | + | - | + | - | Pathogenic | Likely Pathogenic | (Sassi et al., 2014b) |
| *PSEN1* | F237C | Exon 7 | Missense | Yes | - | - | - | - | - | + | + | - | - | + | - | - | + | + | - | - | - | Pathogenic | Likely Pathogenic | (Lanoiselée et al., 2017) |
| *PSEN1* | F237I | Exon 7 | Missense | Yes | - | - | - | - | - | + | + | - | - | + | - | - | + | + | - | - | - | Pathogenic | Likely Pathogenic | (Sodeyama et al., 2001) |
| *PSEN1* | F237L | Exon 7 | Missense | Yes | - | - | - | - | - | + | + | - | - | + | - | - | + | + | - | - | - | Pathogenic | Likely Pathogenic | (Janssen et al., 2003) |
| *PSEN1* | I238M | Exon 7 | Missense | Yes | - | - | - | - | - | + | + | - | - | - | - | - | + | + | - | - | - | Pathogenic | Likely Pathogenic | (Ting et al., 2014) |
| *PSEN1* | K239N | Exon 7 | Missense | Yes | - | - | - | - | - | + | + | - | - | - | - | + | + | + | - | - | - | Pathogenic | Likely Pathogenic | (Lladó et al., 2010) |
| *PSEN1* | L241R | Exon 7 | Missense | Yes | - | - | - | - | - | + | + | - | - | - | - | - | + | + | - | - | - | Pathogenic | Likely Pathogenic | (Lanoiselée et al., 2017) |
| *PSEN1* | P242Lfs (P242LfsX11) | Exon 7 | Frameshift | No | + | - | - | - | - | + | + | - | - | - | - | + | + | + | - | - | - | Pathogenic | Pathogenic | (Wang et al., 2010) |
| *PSEN1* | T245P | Exon 7 | Missense | Yes | - | - | - | - | - | + | + | - | - | - | - | - | + | + | - | - | - | Pathogenic | Likely Pathogenic | (Edwards-Lee et al., 2006) |
| *PSEN1* | A246E | Exon 7 | Missense | Yes | - | - | - | + | - | + | + | - | - | + | - | + | + | + | - | + | - | Pathogenic | Pathogenic | (Sherrington et al., 1995) |
| *PSEN1* | A246P | Exon 7 | Missense | Yes | - | - | - | - | - | + | + | - | - | + | - | - | + | + | - | - | - | Pathogenic | Likely Pathogenic | (Roeber et al., 2015) |
| *PSEN1* | L248P | Exon 7 | Missense | Yes | - | - | - | - | - | + | + | - | - | + | - | + | + | + | - | - | - | Pathogenic | Likely Pathogenic | (Jiao et al., 2014) |
| *PSEN1* | L248R | Exon 7 | Missense | Yes | - | - | - | + | - | + | + | - | - | + | - | - | + | + | - | - | - | Pathogenic | Pathogenic | (Clarimon et al., 2008) |
| *PSEN1* | I249L | Exon 7 | Missense | Yes | - | - | - | + | - | + | + | - | - | - | - | - | + | - | - | + | - | Pathogenic | Pathogenic | (Shen et al., 2019) |
| *PSEN1* | L250F | Exon 7 | Missense | Yes | - | - | - | - | - | + | + | - | - | + | - | + | + | + | - | - | - | Pathogenic | Likely Pathogenic | (Butler et al., 2010) |
| *PSEN1* | L250S | Exon 7 | Missense | Yes | - | - | - | + | - | + | + | - | - | + | - | - | + | + | - | - | - | Pathogenic | Pathogenic | (Hutton et al., 1996) |
| *PSEN1* | L250V | Exon 7 | Missense | Yes | - | - | - | - | - | + | + | - | - | + | - | + | + | + | - | - | - | Pathogenic | Pathogenic | (Furuya et al., 2003) |
| *PSEN1* | Y256N | Exon 7 | Missense | Yes | - | - | - | - | - | + | + | - | - | + | - | + | + | + | - | - | - | Pathogenic | Likely Pathogenic | (Li et al., 2019) |
| *PSEN1* | Y256S | Exon 7 | Missense | Yes | - | - | - | + | - | + | + | - | - | + | - | - | + | + | - | - | - | Pathogenic | Pathogenic | (Miklossy et al., 2003) |
| *PSEN1* | A260G | Exon 8 | Missense | Yes | - | - | - | - | - | + | + | - | - | + | - | - | + | + | - | - | - | Pathogenic | Likely Pathogenic | (Ryman et al., 2014) |
| *PSEN1* | A260V | Exon 8 | Missense | Yes | - | - | - | + | - | + | + | - | - | - | - | - | + | + | - | + | - | Pathogenic | Pathogenic | (Ikeda et al., 1996) |
| *PSEN1* | V261F | Exon 8 | Missense | Yes | - | - | - | + | - | + | + | - | - | + | - | - | + | + | - | + | - | Pathogenic | Pathogenic | (Farlow et al., 2002) |
| *PSEN1* | V261I | Exon 8 | Missense | Yes | - | - | - | - | - | + | + | - | - | + | - | - | + | + | - | - | - | Pathogenic | Likely Pathogenic | (Miravalle et al., 2005) |
| *PSEN1* | V261L | Exon 8 | Missense | Yes | - | - | - | - | - | + | + | - | - | + | - | - | + | + | - | - | - | Pathogenic | Likely Pathogenic | (Jiménez Caballero et al., 2008) |
| *PSEN1* | L262F | Exon 8 | Missense | Yes | - | - | - | + | - | + | - | - | - | + | - | - | + | + | - | - | - | Pathogenic | Pathogenic | (Forsell et al., 1997) |
| *PSEN1* | L262S | Exon 8 | Missense | Yes | - | - | - | - | - | + | + | - | - | + | - | - | + | + | - | - | - | Pathogenic | Likely Pathogenic | (Wang et al., 2019) |
| *PSEN1* | L262V | Exon 8 | Missense | Yes | - | - | - | - | - | + | + | - | - | + | - | + | + | + | - | + | - | Pathogenic | Likely Pathogenic | (Wallon et al., 2012) |
| *PSEN1* | C263F | Exon 8 | Missense | Yes | - | - | - | - | - | + | + | - | - | + | - | - | + | + | - | - | - | Pathogenic | Likely Pathogenic | (Janssen et al., 2003) |
| *PSEN1* | C263R | Exon 8 | Missense | Yes | - | - | - | + | - | + | + | - | - | + | - | - | + | + | - | - | - | Pathogenic | Pathogenic | (Wasco et al., 1995) |
| *PSEN1* | P264L | Exon 8 | Missense | Yes | - | - | - | + | - | + | + | - | - | - | - | + | + | + | - | + | - | Pathogenic | Pathogenic | (Campion et al., 1995b) |
| *PSEN1* | G266S | Exon 8 | Missense | No | - | - | - | + | - | + | + | - | - | - | - | + | + | + | - | - | - | Pathogenic | Pathogenic | (Matsubara-Tsutsui et al., 2002) |
| *PSEN1* | P267A | Exon 8 | Missense | No | - | - | - | - | - | + | + | - | - | + | - | - | + | + | - | - | - | Pathogenic | Likely Pathogenic | (Ringman et al., 2016) |
| *PSEN1* | P267L | Exon 8 | Missense | No | - | - | - | - | - | + | + | - | - | + | - | - | + | + | - | - | - | Pathogenic | Likely Pathogenic | (Kowalska et al., 2003) |
| *PSEN1* | P267S | Exon 8 | Missense | No | - | - | - | - | - | + | + | - | - | + | - | + | + | + | - | - | - | Pathogenic | Likely Pathogenic | (1995) |
| *PSEN1* | R269G | Exon 8 | Missense | No | - | - | - | + | - | + | + | - | - | + | - | - | + | + | - | + | - | Pathogenic | Pathogenic | (Perez-Tur et al., 1996) |
| *PSEN1* | R269H | Exon 8 | Missense | No | - | - | - | - | - | + | - | - | - | + | - | - | + | + | - | + | - | Pathogenic | Likely Pathogenic | (Gómez-Isla et al., 1997) |
| *PSEN1* | L271V | Exon 8 | Missense | No | - | - | - | - | - | + | + | - | - | - | - | + | + | + | - | - | - | Pathogenic | Likely Pathogenic | (Kwok et al., 2003) |
| *PSEN1* | V272A | Exon 8 | Missense | No | - | - | - | + | - | + | + | - | - | + | - | - | + | + | - | + | - | Pathogenic | Pathogenic | (Jimenez-Escrig et al., 2004) |
| *PSEN1* | V272D | Exon 8 | Missense | No | - | - | - | + | - | + | + | - | - | + | - | - | + | + | - | - | - | Pathogenic | Pathogenic | (Mengel et al., 2020) |
| *PSEN1* | E273A | Exon 8 | Missense | No | - | - | - | + | - | + | + | - | - | + | - | - | + | + | - | - | - | Pathogenic | Pathogenic | (Kamimura et al., 1998) |
| *PSEN1* | E273G | Exon 8 | Missense | No | - | - | - | - | - | + | + | - | - | + | - | - | + | + | - | + | - | Pathogenic | Likely Pathogenic | (Wallon et al., 2012) |
| *PSEN1* | T274R | Exon 8 | Missense | No | - | - | - | - | - | + | + | - | - | - | - | - | + | + | - | - | - | Pathogenic | Likely Pathogenic | (Rogaeva et al., 2001) |
| *PSEN1* | A275V | Exon 8 | Missense | No | - | - | - | + | - | + | + | - | - | - | - | + | + | + | - | - | - | Pathogenic | Pathogenic | (Luedecke et al., 2014) |
| *PSEN1* | R278I | Exon 8 | Missense | No | - | - | - | + | - | + | + | - | - | + | - | - | + | + | - | - | - | Pathogenic | Pathogenic | (Godbolt et al., 2004) |
| *PSEN1* | R278K | Exon 8 | Missense | No | - | - | - | + | - | + | + | - | - | + | - | - | + | + | - | - | - | Pathogenic | Pathogenic | (Assini et al., 2003) |
| *PSEN1* | R278S | Exon 8 | Missense | No | - | - | - | - | - | + | + | - | - | + | - | - | + | + | - | - | - | Pathogenic | Likely Pathogenic | (Raman et al., 2007) |
| *PSEN1* | R278T | Exon 8 | Missense | No | - | - | - | - | - | + | + | - | - | + | - | - | + | + | - | - | - | Pathogenic | Likely Pathogenic | (Kwok et al., 1997) |
| *PSEN1* | E280A | Exon 8 | Missense | No | - | - | - | + | - | + | + | - | - | + | - | + | + | + | - | - | - | Pathogenic | Pathogenic | (Lalli et al., 2014) |
| *PSEN1* | E280G | Exon 8 | Missense | No | - | - | - | + | - | + | + | - | - | + | - | - | + | + | - | + | - | Pathogenic | Pathogenic | (1995) |
| *PSEN1* | E280K | Exon 8 | Missense | No | - | - | - | - | - | + | + | - | - | + | - | + | + | + | - | - | - | Pathogenic | Pathogenic | (Ch'ng et al., 2015) |
| *PSEN1* | L282F | Exon 8 | Missense | No | - | - | - | - | - | + | + | - | - | + | - | - | + | + | - | - | - | Pathogenic | Likely Pathogenic | (Hamaguchi et al., 2009) |
| *PSEN1* | L282P | Exon 8 | Missense | No | - | - | - | - | - | + | + | - | - | + | - | - | + | + | - | - | - | Pathogenic | Likely Pathogenic | (Kim et al., 2020) |
| *PSEN1* | L282R | Exon 8 | Missense | No | - | - | - | + | - | + | + | - | - | + | - | - | + | + | - | + | - | Pathogenic | Pathogenic | (Aldudo et al., 1998) |
| *PSEN1* | L282V | Exon 8 | Missense | No | - | - | - | - | - | + | + | - | - | + | - | - | + | + | - | - | - | Pathogenic | Likely Pathogenic | (Dermaut et al., 2001) |
| *PSEN1* | F283L | Exon 8 | Missense | No | - | - | - | - | - | + | + | - | - | - | - | + | + | + | - | + | - | Pathogenic | Likely Pathogenic | (Scahill et al., 2013) |
| *PSEN1* | P284L | Exon 8 | Missense | No | - | - | - | - | - | + | + | - | - | - | - | - | + | + | - | - | - | Pathogenic | Likely Pathogenic | (Tabira et al., 2002) |
| *PSEN1* | P284S | Exon 8 | Missense | No | - | - | - | - | - | + | + | - | - | + | - | - | + | + | - | - | BS3 | Pathogenic | VUS | (Marrosu et al., 2006) |
| *PSEN1* | A285S | Exon 8 | Missense | No | - | - | - | - | - | + | + | - | - | + | - | - | + | + | - | - | - | Pathogenic | Likely Pathogenic | (Kim et al., 2020) |
| *PSEN1* | A285V | Exon 8 | Missense | No | - | - | - | - | - | + | + | - | - | + | - | - | + | + | - | + | - | Pathogenic | Likely Pathogenic | (Ikeda et al., 1996) |
| *PSEN1* | L286P | Exon 8 | Missense | No | - | - | - | - | - | + | + | - | - | + | - | + | + | + | - | - | - | Pathogenic | Likely Pathogenic | (Sánchez-Valle et al., 2007) |
| *PSEN1* | L286V | Exon 8 | Missense | No | - | - | - | + | - | + | + | - | - | + | - | + | + | + | - | + | - | Pathogenic | Pathogenic | (Sherrington et al., 1995) |
| *PSEN1* | T291A | Exon 9 | Missense | No | - | - | - | - | - | + | + | - | - | + | - | - | + | - | - | - | - | Pathogenic | Likely Pathogenic | (Ryan et al., 2016) |
| *PSEN1* | T291P | Exon 9 | Missense | No | - | - | - | + | - | + | + | - | - | + | - | - | + | + | - | - | - | Pathogenic | Pathogenic | (Dumanchin et al., 2006) |
| *PSEN1* | P303L | Exon 9 | Missense | No | - | - | - | - | - | + | - | - | - | - | - | - | + | - | - | - | - | VUS | VUS | (Koriath et al., 2020) |
| *PSEN1* | K311R | Exon 9 | Missense | No | - | - | - | + | - | + | - | - | - | - | - | + | + | - | - | - | - | Pathogenic | Likely pathogenic | (Dong et al., 2017) |
| *PSEN1* | E318G | Exon 9 | Missense | No | - | - | - | - | - | + | - | - | - | - | - | - | + | - | - | - | ~~-~~ | VUS | VUS | (Sandbrink et al., 1996) |
| *PSEN1* | D333G | Exon 10 | Missense | No | - | - | - | - | - | + | - | - | - | - | - | - | + | - | - | - | BS3 | VUS | VUS | (Li et al., 2006) |
| *PSEN1* | R352C | Exon 10 | Missense | No | - | - | - | - | - | + | - | - | - | - | - | - | + | - | - | - | BS3 | VUS | VUS | (Jiang et al., 2015) |
| *PSEN1* | R352_S353insR | Exon 10 | Indel | No | - | - | - | - | - | + | - | - | + | - | - | - | + | - | - | - | ~~-~~ | VUS | VUS | (Rogaeva et al., 2001) |
| *PSEN1* | T354I | Exon 10 | Missense | No | - | - | - | - | - | + | + | - | - | - | - | - | + | - | - | - | - | VUS | VUS | (Rogaeva et al., 2001) |
| *PSEN1* | P355S | Exon 10 | Missense | No | - | - | - | - | - | - | - | - | - | - | - | - | + | - | - | - | - | VUS | VUS | (Monacelli et al., 2019) |
| *PSEN1* | R358Q | Exon 10 | Missense | No | - | - | - | + | - | + | - | - | - | - | - | - | + | - | - | - | - | VUS | Likely pathogenic | (Rogaeva et al., 2001) |
| *PSEN1* | A360T | Exon 10 | Missense | No | - | - | - | - | - | + | - | - | - | - | - | - | + | - | - | - | - | Pathogenic | VUS | (Lanoiselée et al., 2017) |
| *PSEN1* | S365A | Exon 10 | Missense | No | - | - | - | - | - | + | - | - | - | - | - | + | + | - | - | - | - | VUS | VUS | (Maesako et al., 2017) |
| *PSEN1* | S365Y | Exon 10 | Missense | No | - | - | - | - | - | + | + | - | - | - | - | - | + | - | - | - | - | VUS | VUS | (Rogaeva et al., 2001) |
| *PSEN1* | G371C | Exon 10 | Missense | No | - | - | - | - | - | + | + | - | - | - | - | - | + | - | - | - | - | VUS | VUS | (Perrone et al., 2020) |
| *PSEN1* | R377M | Exon 11 | Missense | No | - | - | - | - | - | + | + | - | - | + | - | - | + | + | - | - | - | Pathogenic | Likely Pathogenic | (Janssen et al., 2003) |
| *PSEN1* | R377W | Exon 11 | Missense | No | - | - | - | + | - | + | + | - | - | + | - | - | + | + | - | + | - | Pathogenic | Pathogenic | (Wallon et al., 2012) |
| *PSEN1* | G378R | Exon 11 | Missense | No | - | - | - | - | - | + | + | - | - | + | - | - | + | + | - | - | - | Pathogenic | Likely Pathogenic | (Ramos-Campoy et al., 2020) |
| *PSEN1* | G378E | Exon 11 | Missense | No | - | - | - | + | - | + | + | - | - | + | - | + | + | + | - | + | - | Pathogenic | Pathogenic | (Besançon et al., 1998) |
| *PSEN1* | G378V | Exon 11 | Missense | No | - | - | - | - | - | + | + | - | - | + | - | + | + | + | - | + | - | Pathogenic | Likely Pathogenic | (Janssen et al., 2003) |
| *PSEN1* | G378fs | Exon 11 | Indel | No | + | - | - | - | - | + | + | - | - | - | - | - | - | + | - | - | - | VUS | Pathogenic | (El Kadmiri et al., 2014) |
| *PSEN1* | L381F | Exon 11 | Missense | Yes | - | - | - | - | - | + | + | - | - | + | - | + | + | + | - | - | - | Pathogenic | Likely Pathogenic | (Dolzhanskaya et al., 2014) |
| *PSEN1* | L381V | Exon 11 | Missense | Yes | - | - | - | + | - | + | + | - | - | + | - | - | + | + | - | + | - | Pathogenic | Pathogenic | (Dintchov Traykov et al., 2009) |
| *PSEN1* | G384A | Exon 11 | Missense | Yes | - | - | - | + | - | + | + | - | - | - | - | - | + | + | - | + | - | Pathogenic | Pathogenic | (Cruts et al., 1995) |
| *PSEN1* | F386I | Exon 11 | Missense | Yes | - | - | - | - | - | + | + | - | - | + | - | + | + | + | - | - | - | Pathogenic | Likely Pathogenic | (Shea et al., 2017) |
| *PSEN1* | F386L | Exon 11 | Missense | Yes | - | - | - | - | - | + | + | - | - | + | - | + | + | + | - | - | - | Pathogenic | Likely Pathogenic | (Yagi et al., 2014) |
| *PSEN1* | F386S | Exon 11 | Missense | Yes | - | - | - | + | - | + | + | - | - | + | - | - | + | + | - | + | - | Pathogenic | Pathogenic | (Finckh et al., 2000b) |
| *PSEN1* | F388L | Exon 11 | Missense | Yes | - | - | - | + | - | + | + | - | - | - | - | + | + | + | - | - | - | Pathogenic | Pathogenic | (Zhan et al., 2017) |
| *PSEN1* | Y389H | Exon 11 | Missense | Yes | - | - | - | - | - | + | + | - | - | + | - | - | + | + | - | + | - | Pathogenic | Likely Pathogenic | (Park et al., 2020) |
| *PSEN1* | Y389S | Exon 11 | Missense | Yes | - | - | - | - | - | + | + | - | - | + | - | - | + | + | - | - | - | Pathogenic | Likely Pathogenic | (Kim et al., 2020) |
| *PSEN1* | S390I | Exon 11 | Missense | Yes | - | - | - | - | - | + | + | - | - | + | - | - | + | + | - | - | - | Pathogenic | Likely Pathogenic | (Campion et al., 1999) |
| *PSEN1* | S390N | Exon 11 | Missense | Yes | - | - | - | - | - | + | + | - | - | + | - | - | + | + | - | - | - | Pathogenic | Likely Pathogenic | (Nicolas et al., 2016a) |
| *PSEN1* | V391F | Exon 11 | Missense | Yes | - | - | - | + | - | + | + | - | - | + | - | + | + | + | - | + | - | Pathogenic | Pathogenic | (Finckh et al., 2000b) |
| *PSEN1* | V391G | Exon 11 | Missense | Yes | - | - | + | - | - | + | + | - | - | + | - | - | + | + | - | - | - | Pathogenic | Pathogenic | (Lou et al., 2017) |
| *PSEN1* | L392P | Exon 11 | Missense | Yes | - | - | - | - | - | + | + | - | - | + | - | - | + | + | - | - | - | Pathogenic | Likely Pathogenic | (Tedde et al., 2000) |
| *PSEN1* | L392V | Exon 11 | Missense | Yes | - | - | - | + | - | + | + | - | - | + | - | + | + | + | - | + | - | Pathogenic | Pathogenic | (Campion et al., 1995a) |
| *PSEN1* | V393F | Exon 11 | Missense | Yes | - | - | - | - | - | + | + | - | - | - | - | - | + | + | - | - | - | Pathogenic | Likely Pathogenic | (Koriath et al., 2020) |
| *PSEN1* | G394V | Exon 11 | Missense | Yes | - | - | - | - | - | + | + | - | - | - | - | - | + | + | - | + | - | Pathogenic | Likely Pathogenic | (Rogaeva et al., 2001) |
| *PSEN1* | A396T | Exon 11 | Missense | Yes | - | - | - | + | - | + | + | - | - | - | - | - | + | + | - | + | - | Pathogenic | Pathogenic | (Lohmann et al., 2012) |
| *PSEN1* | N405S | Exon 11 | Missense | No | - | - | - | - | - | + | + | - | - | - | - | - | + | - | - | - | - | Pathogenic | VUS | (Yasuda et al., 2000) |
| *PSEN1* | I408T | Exon 11 | Missense | Yes | - | - | - | - | - | + | + | - | - | - | - | - | + | + | - | - | - | Pathogenic | Likely Pathogenic | (Tedde et al., 2016) |
| *PSEN1* | A409T | Exon 11 | Missense | Yes | - | - | - | - | - | + | + | - | - | - | - | - | + | + | - | - | - | Pathogenic | Likely Pathogenic | (Aldudo et al., 1999) |
| *PSEN1* | C410Y | Exon 11 | Missense | Yes | - | - | - | - | - | + | + | - | - | - | - | + | + | + | - | + | - | Pathogenic | Likely Pathogenic | (Sherrington et al., 1995) |
| *PSEN1* | V412I | Exon 11 | Missense | Yes | - | - | - | - | - | + | - | - | - | - | - | - | + | - | - | - | - | VUS | VUS | (Bernardi et al., 2009) |
| *PSEN1* | I416T | Exon 12 | Missense | Yes | - | - | - | - | - | + | + | - | - | - | - | + | + | - | - | - | - | Pathogenic | Likely Pathogenic | (Ramirez Aguilar et al., 2019) |
| *PSEN1* | G417A | Exon 12 | Missense | Yes | - | - | - | - | - | + | + | - | - | + | - | - | + | + | - | - | - | Pathogenic | Likely Pathogenic | (Giau et al., 2018) |
| *PSEN1* | G417S | Exon 12 | Missense | Yes | - | - | - | - | - | + | + | - | - | + | - | - | + | + | - | - | - | Pathogenic | Likely Pathogenic | (Miki et al., 2019) |
| *PSEN1* | L418F | Exon 12 | Missense | Yes | - | - | - | + | - | + | + | - | - | - | - | - | + | + | - | - | - | Pathogenic | Pathogenic | (Rogaeva et al., 2001) |
| *PSEN1* | L420R | Exon 12 | Missense | Yes | - | - | - | + | - | + | + | - | - | - | - | + | + | + | - | + | - | Pathogenic | Pathogenic | (Shrimpton et al., 2007) |
| *PSEN1* | L424F | Exon 12 | Missense | Yes | - | - | - | - | - | + | + | - | - | + | - | - | + | - | - | - | - | Pathogenic | Likely Pathogenic | (Mehrabian et al., 2006) |
| *PSEN1* | L424H | Exon 12 | Missense | Yes | - | - | - | - | - | + | + | - | - | + | - | - | + | + | - | + | - | Pathogenic | Likely Pathogenic | (Finckh et al., 2000b) |
| *PSEN1* | L424P | Exon 12 | Missense | Yes | - | - | - | - | - | + | + | - | - | + | - | - | + | + | - | - | - | Pathogenic | Likely Pathogenic | (Guven et al., 2019) |
| *PSEN1* | L424R | Exon 12 | Missense | Yes | - | - | - | - | - | + | + | - | - | + | - | - | + | + | - | + | - | Pathogenic | Likely Pathogenic | (Kowalska et al., 1999) |
| *PSEN1* | L424V | Exon 12 | Missense | Yes | - | - | - | + | - | + | + | - | - | + | - | - | + | - | - | + | - | Pathogenic | Pathogenic | (Robles et al., 2009) |
| *PSEN1* | A426P | Exon 12 | Missense | Yes | - | - | - | - | - | + | + | - | - | - | - | - | + | + | - | + | - | Pathogenic | Likely Pathogenic | (Poorkaj et al., 1998) |
| *PSEN1* | A431E | Exon 12 | Missense | No | - | - | - | - | - | + | + | - | - | + | - | + | + | + | - | + | - | Pathogenic | Likely Pathogenic | (Yescas et al., 2006) |
| *PSEN1* | A431V | Exon 12 | Missense | No | - | - | - | - | - | + | + | - | - | + | - | + | + | + | - | - | - | Pathogenic | Likely Pathogenic | (Matsushita et al., 2002) |
| *PSEN1* | P433S | Exon 12 | Missense | Yes | - | - | - | + | - | + | + | - | - | - | - | - | + | + | - | + | - | Pathogenic | Pathogenic | (Shen et al., 2019) |
| *PSEN1* | A434C | Exon 12 | Missense | Yes | - | - | - | + | - | + | + | - | - | + | - | - | + | + | - | - | - | Pathogenic | Pathogenic | (Devi et al., 2000) |
| *PSEN1* | A434T | Exon 12 | Missense | Yes | - | - | - | - | - | + | + | - | - | + | - | + | + | + | - | + | - | Pathogenic | Likely Pathogenic | (Jiao et al., 2014) |
| *PSEN1* | L435F | Exon 12 | Missense | Yes | - | - | - | - | - | + | + | - | - | - | - | + | + | + | - | + | - | Pathogenic | Likely Pathogenic | (Rogaeva et al., 2001) |
| *PSEN1* | P436Q | Exon 12 | Missense | Yes | - | - | - | - | - | + | + | - | - | + | + | - | + | + | - | + | - | Pathogenic | Likely Pathogenic | (Taddei et al., 1998) |
| *PSEN1* | P436S | Exon 12 | Missense | Yes | - | - | - | + | - | + | + | - | - | + | - | - | + | + | - | - | - | Pathogenic | Pathogenic | (Palmer et al., 1999) |
| *PSEN1* | I437V | Exon 12 | Missense | Yes | - | - | - | + | - | + | - | - | - | - | - | - | + | - | - | - | - | Pathogenic | Likely pathogenic | (Nicolas et al., 2016a) |
| *PSEN1* | I439S | Exon 12 | Missense | Yes | - | - | - | - | - | + | + | - | - | + | - | - | + | + | - | - | - | Pathogenic | Likely Pathogenic | (Gómez-Tortosa et al., 2010) |
| *PSEN1* | I439V | Exon 12 | Missense | Yes | - | - | - | - | - | + | + | - | - | + | - | - | + | - | - | - | - | VUS | Likely Pathogenic | (Rogaeva et al., 2001) |
| *PSEN1* | T440del | Exon 12 | Indel | Yes | - | - | - | + | - | + | + | - | + | - | - | - | + | + | - | - | - | Pathogenic | Pathogenic | (Ishikawa et al., 2005) |
| *PSEN1* | c.869-1G>A | Intron 8/11 | splicing | NA | - | - | - | - | - | + | + | - | - | - | - | - | + | + | - | - | - | Pathogenic | Likely Pathogenic | (Koriath et al., 2020) |
| *PSEN1* | c.869-2A>G | Intron 8/11 | splicing | NA | - | - | - | - | - | + | + | - | - | - | - | - | + | + | - | - | - | Pathogenic | Likely Pathogenic | (Blauwendraat et al., 2018) |
| *PSEN1* | c.869-22_869-23ins18 | Intron 8, Exon 9 | Indel | Yes | - | - | - | - | - | + | + | - | - | - | - | - | + | + | - | - | - | Pathogenic | Likely Pathogenic | (Dumanchin et al., 2006) |
| *PSEN1* | I238_K239insI | Exon 7 | Indel | Yes | - | - | - | - | - | + | + | - | + | - | - | - | + | + | - | - | - | Pathogenic | Likely Pathogenic | (Roeber et al., 2015) |
| *PSEN1* | L171_ L172insY | Exon 6 | Indel | Yes | - | - | - | - | - | + | + | - | + | - | - | - | + | + | - | - | - | Pathogenic | Likely Pathogenic | (Koriath et al., 2020) |
| *PSEN1* | S290C;T291_S319del (ΔE9) | Intron 8, Exon 9 | Missense,CNV | NA | + | - | - | + | - | + | + | - | - | - | - | + | + | + | - | + | - | Pathogenic | Pathogenic | (Crook et al., 1998) |
| *PSEN1* | S290C;T291_S319del (ΔE9Finn) | Intron 8, Exon 9 | Missense,CNV | NA | + | - | - | + | - | + | + | - | - | - | - | + | + | + | - | + | - | Pathogenic | Pathogenic | (Crook et al., 1998) |
| *PSEN1* | S290W;S291_R377del | Exons 9-10, Introns 8-10 | Missense,CNV | NA | + | - | - | - | - | + | + | - | - | - | - | - | + | + | - | - | - | Pathogenic | Pathogenic | (Le Guennec et al., 2017) |
| *PSEN1* | S290C;T291_S319del A>G | Intron 8, Exon 9 | Missense,CNV | NA | + | - | - | - | - | + | + | - | - | - | + | - | + | + | - | + | - | Pathogenic | Pathogenic | (Rovelet-Lecrux et al., 2015) |
| *PSEN1* | S290C;T291_S319del G>A | Intron 8, Exon 9 | Missense,CNV | NA | + | - | - | - | - | + | + | - | - | - | - | - | + | + | - | + | - | Pathogenic | Pathogenic | (Sato et al., 1998) |
| *PSEN1* | S290C;T291_S319del G>T | Intron 8, Exon 9 | Missense,CNV | NA | + | - | - | - | - | + | + | - | - | - | - | + | + | + | - | + | - | Pathogenic | Pathogenic | (Hutton et al., 1996) |
| *PSEN2* | T18M | Exon 3 | Missense | No | - | - | - | - | - | - | - | - | - | - | - | - | - | - | - | - | - | VUS | VUS | (Blauwendraat et al., 2016) |
| *PSEN2* | R29H | Exon 3 | Missense | No | - | - | - | - | - | - | - | - | - | - | - | - | - | - | - | - | - | Not Pathogenic | VUS | (Guerreiro et al., 2010) |
| *PSEN2* | G34S | Exon 3 | Missense | No | - | - | - | - | - | - | - | - | - | - | - | - | - | - | - | - | BS3+BS4 | VUS | Benign | (Sleegers et al., 2004) |
| *PSEN2* | R62C | Exon 4 | Missense | No | - | - | - | - | - | - | - | - | - | - | - | - | - | - | - | - | - | VUS | VUS | (Brouwers et al., 2008) |
| *PSEN2* | R62H | Exon 4 | Missense | No | - | - | - | - | - | - | - | - | - | - | - | - | - | - | - | - | BS1+BS4 | Not Pathogenic | Benign | (Cruts et al., 1998) |
| *PSEN2* | P69A | Exon 4 | Missense | No | - | - | - | - | - | - | - | - | - | - | - | - | - | - | - | - | - | VUS | VUS | (Dobricic et al., 2012) |
| *PSEN2* | R71W | Exon 4 | Missense | No | - | - | - | - | - | - | - | - | - | - | - | - | - | - | - | - | BS3 | Not Pathogenic | VUS | (Sleegers et al., 2004) |
| *PSEN2* | K82R | Exon 4 | Missense | No | - | - | - | - | - | - | + | - | - | - | - | - | - | - | - | - | - | VUS | VUS | (Shi et al., 2015) |
| *PSEN2* | A85V | Exon 4 | Missense | No | - | - | - | - | - | - | - | - | - | - | + | - | - | + | - | - | - | Pathogenic | VUS | (Piscopo et al., 2008) |
| *PSEN2* | V101M | Exon 4 | Missense | Yes | - | - | - | - | - | - | - | - | - | - | - | - | - | + | - | - | - | VUS | VUS | (Sala Frigerio et al., 2015) |
| *PSEN2* | K115Efs* | Exon 4 | frameshift | No | + | - | - | - | - | - | - | - | - | - | - | - | - | + | - | + | - | Pathogenic | Likely Pathogenic | (Jayadev et al., 2010) |
| *PSEN2* | T122P | Exon 5 | Missense | No | - | - | - | + | - | - | + | - | - | - | - | - | - | + | - | + | - | Pathogenic | Likely Pathogenic | (Finckh et al., 2000b) |
| *PSEN2* | T122R | Exon 5 | Missense | No | - | - | - | - | - | - | + | - | - | + | - | - | - | + | - | - | - | Pathogenic | VUS | (Binetti et al., 2003) |
| *PSEN2* | P123L | Exon 5 | Missense | No | - | - | - | - | - | - | + | - | - | - | - | - | - | + | - | - | BS4 | Pathogenic | VUS | (Xia et al., 2015) |
| *PSEN2* | E126fs | Exon 5 | frameshift | No | + | - | - | - | - | - | + | - | - | - | - | - | - | + | - | - | - | VUS | Pathogenic | (El Kadmiri et al., 2014) |
| *PSEN2* | E126K | Exon 5 | Missense | No | - | - | - | - | - | - | + | - | - | - | - | + | - | - | - | - | - | Pathogenic | VUS | (Müller et al., 2014) |
| *PSEN2* | S130L | Exon 5 | Missense | No | - | - | - | - | - | - | - | - | - | - | - | - | - | + | - | - | BS3 | VUS | VUS | (Walker et al., 2005) |
| *PSEN2* | V139M | Exon 5 | Missense | No | - | - | - | - | - | - | - | - | - | - | - | - | - | - | - | - | - | VUS | VUS | (Bernardi et al., 2008) |
| *PSEN2* | N141D | Exon 5 | Missense | Yes | - | - | - | - | - | - | + | - | - | + | + | - | - | + | - | - | - | Pathogenic | Likely Pathogenic | (Wang et al., 2019) |
| *PSEN2* | N141I | Exon 5 | Missense | Yes | - | - | - | + | - | - | + | - | - | + | - | + | - | + | - | + | - | Pathogenic | Pathogenic | (Levy-Lahad et al., 1995) |
| *PSEN2* | N141Y | Exon 5 | Missense | Yes | - | - | - | - | - | - | + | - | - | + | - | + | - | + | - | - | - | Pathogenic | Likely Pathogenic | (Niu et al., 2014) |
| *PSEN2* | L143H | Exon 5 | Missense | Yes | - | - | - | - | - | - | + | - | - | - | - | - | - | + | - | - | - | VUS | VUS | (Guerreiro et al., 2010) |
| *PSEN2* | V148I | Exon 5 | Missense | Yes | - | - | - | - | - | - | + | - | - | - | - | - | - | - | - | - | BS3 | VUS | VUS | (Lao et al., 1998) |
| *PSEN2* | I149T | Exon 5 | Missense | Yes | - | - | - | - | - | - | + | - | - | - | - | - | - | + | - | - | - | VUS | VUS | (Perrone et al., 2020) |
| *PSEN2* | V150M | Exon 5 | Missense | Yes | - | - | - | - | - | - | + | - | - | - | - | - | - | - | - | - | - | VUS | VUS | (Gao et al., 2019) |
| *PSEN2* | T153S | Exon 5 | Missense | Yes | - | - | - | - | - | - | + | - | - | - | - | - | - | + | - | - | - | VUS | VUS | (Perrone et al., 2020) |
| *PSEN2* | K161R | Exon 5 | Missense | No | - | - | - | - | - | - | - | - | - | - | - | - | - | + | - | - | - | Pathogenic | VUS | (Wallon et al., 2012) |
| *PSEN2* | R163H | Exon 5 | Missense | No | - | - | - | - | - | - | - | - | - | - | - | - | - | + | - | - | BS4 | Not Pathogenic | VUS | (Puschmann et al., 2009) |
| *PSEN2* | R163C | Exon 5 | Missense | No | - | - | - | - | - | - | + | - | - | - | - | - | - | + | - | - | - | VUS | VUS | (Gao et al., 2019) |
| *PSEN2* | H169N | Exon 6 | Missense | Yes | - | - | - | - | - | - | - | - | - | - | - | - | - | - | - | - | ~~-~~ | VUS | VUS | (Shi et al., 2015) |
| *PSEN2* | M174V | Exon 6 | Missense | Yes | - | - | - | - | - | - | - | - | - | - | - | - | - | - | - | - | BS2+BS4 | Not Pathogenic | Benign | (Clarimon et al., 2008) |
| *PSEN2* | S175C | Exon 6 | Missense | Yes | - | - | - | - | - | - | - | - | - | - | - | - | - | + | - | - | - | Pathogenic | VUS | (Piscopo et al., 2010) |
| *PSEN2* | G212V | Exon 7 | Missense | Yes | - | - | - | - | - | - | + | - | - | - | - | + | - | + | - | - | - | Pathogenic | VUS | (Marín-Muñoz et al., 2016) |
| *PSEN2* | V214L | Exon 7 | Missense | Yes | - | - | - | - | - | - | - | - | - | - | - | - | - | - | - | + | ~~-~~ | VUS | VUS | (Youn et al., 2014) |
| *PSEN2* | Q228L | Exon 7 | Missense | Yes | - | - | - | - | - | - | - | - | - | - | - | - | - | + | - | - | - | VUS | VUS | (Zekanowski et al., 2003) |
| *PSEN2* | Y231C | Exon 7 | Missense | Yes | - | - | - | - | - | - | - | - | - | - | - | - | - | + | - | - | - | VUS | VUS | (Marcon et al., 2009) |
| *PSEN2* | I235F | Exon 7 | Missense | Yes | - | - | - | - | - | - | + | - | - | - | - | - | - | + | - | - | BS4 | Not Pathogenic | VUS | (Lee et al., 2014) |
| *PSEN2* | S236S | Exon 7 | Missense | Yes | - | - | - | - | - | - | - | - | - | - | - | - | - | - | - | - | BP7 | VUS | VUS | (Coppola et al., 2021) |
| *PSEN2* | A237V | Exon 7 | Missense | Yes | - | - | - | - | - | - | - | - | - | - | - | - | - | - | - | - | - | VUS | VUS | (Sassi et al., 2014a) |
| *PSEN2* | L238F | Exon 7 | Missense | Yes | - | - | - | - | - | - | - | - | - | - | - | - | - | - | - | - | - | VUS | VUS | (Sala Frigerio et al., 2015) |
| *PSEN2* | L238P | Exon 7 | Missense | Yes | - | - | - | - | - | - | + | - | - | - | - | - | - | + | - | - | - | VUS | VUS | (Blauwendraat et al., 2016) |
| *PSEN2* | M239I | Exon 7 | Missense | Yes | - | - | - | + | - | - | + | - | - | + | + | - | - | - | - | + | - | Pathogenic | Pathogenic | (Finckh et al., 2000a) |
| *PSEN2* | M239V | Exon 7 | Missense | Yes | - | - | - | + | - | - | + | - | - | + | - | - | + | - | - | + | - | Pathogenic | Pathogenic | (Rogaev et al., 1995) |
| *PSEN2* | A252T | Exon 7 | Missense | Yes | - | - | - | - | - | - | - | - | - | - | - | - | - | - | - | - | ~~-~~ | Not Pathogenic | VUS | (Guerreiro et al., 2010) |
| *PSEN2* | A258T | Exon 7 | Missense | Yes | - | - | - | - | - | - | - | - | - | - | - | - | - | - | - | - | - | Not Pathogenic | VUS | (Sala Frigerio et al., 2015) |
| *PSEN2* | A258V | Exon 7 | Missense | Yes | - | - | - | - | - | - | + | - | - | - | - | - | - | - | - | - | - | VUS | VUS | (Yagi et al., 2014) |
| *PSEN2* | P287P | Exon 8 | Missense | No | - | - | - | - | - | - | - | - | - | - | - | - | - | - | - | - | ~~-~~ | VUS | VUS | (Seo et al., 2020) |
| *PSEN2* | T301M | Exon 7 | Missense | No | - | - | - | - | - | - | - | - | - | - | - | - | - | - | - | - | BS3 | VUS | VUS | (Croes et al., 2004) |
| *PSEN2* | K306fs | Exon 9 | frameshift | No | + | - | - | - | - | - | + | - | - | - | - | - | - | + | - | - | - | VUS | Pathogenic | (El Kadmiri et al., 2014) |
| *PSEN2* | P334A | Exon 10 | Missense | No | - | - | - | - | - | - | - | - | - | - | - | - | - | - | - | - | BS4 | Not Pathogenic | VUS | (Lee et al., 2014) |
| *PSEN2* | P334R | Exon 10 | Missense | No | - | - | - | - | - | - | - | - | - | - | - | - | - | - | - | - | BS4 | Not Pathogenic | VUS | (Lleó et al., 2002b) |
| *PSEN2* | P348L | Exon 10 | Missense | No | - | - | - | - | - | - | + | - | - | - | - | - | - | - | - | - | - | VUS | VUS | (Blauwendraat et al., 2016) |
| *PSEN2* | A377V | Exon 11 | Missense | Yes | - | - | - | - | - | - | + | - | - | - | - | - | - | + | - | - | BS4 | VUS | VUS | (Lee et al., 2014) |
| *PSEN2* | A379D | Exon 11 | Missense | Yes | - | - | - | - | - | - | + | - | - | + | + | - | - | + | - | - | - | Pathogenic | Likely Pathogenic | (Wang et al., 2019) |
| *PSEN2* | V393M | Exon 11 | Missense | Yes | - | - | - | - | - | - | - | - | - | - | - | - | - | - | - | - | BS3 | VUS | Benign | (Lindquist et al., 2008a) |
| *PSEN2* | T421M | Exon 12 | Missense | Yes | - | - | - | - | - | - | - | - | - | - | + | - | - | + | - | - | - | Pathogenic | VUS | (Yagi et al., 2014) |
| *PSEN2* | T430M | Exon 12 | Missense | Yes | - | - | - | - | - | - | - | - | - | - | - | - | - | + | - | - | - | VUS | VUS | (Ezquerra et al., 2003) |
| *PSEN2* | P436L | Exon 12 | Missense | No | - | - | - | - | - | - | - | - | - | - | - | - | - | + | - | - | - | VUS | VUS | (Han et al., 2020) |
| *PSEN2* | D439A | Exon 12 | Missense | No | - | - | - | - | - | - | - | - | - | - | - | - | - | + | - | - | BS3 | VUS | VUS | (Lleó et al., 2001) |
| *PSEN2* | G359fs (Intron 11/12 delA) | Intron 11/12 | frameshift | NA | + | - | - | + | - | - | + | - | - | - | - | - | - | + | - | - | - | VUS | Pathogenic | (Perrone et al., 2018) |
| *PSEN2* | G359fs (Intron 11/12 delAG) | Intron 11/12 | frameshift | NA | + | - | - | - | - | - | + | - | - | - | - | - | - | + | - | + | - | VUS | Pathogenic | (Perrone et al., 2018) |
| *PSEN2* | Intron 9/12 C>T | Intron 9/12 | splicing | NA | + | - | - | - | - | - | + | - | - | - | - | - | - | - | - | - | - | VUS | Likely pathogenic | (Wang et al., 2019) |
| *PSEN2* | K82fs | Exon 4 | frameshift | No | + | - | - | - | - | - | - | - | - | - | - | - | - | - | - | - | - | VUS | VUS | (Perrone et al., 2018) |

VUS: variant of uncertain significance; TM: transmembrane; Benign criteria include 12 benign criteria.

**References**

Alzheimer's Disease Collaborative Group (1995). The structure of the presenilin 1 (S182) gene and identification of six novel mutations in early onset AD families. *Nat Genet* 11(2)**,** 219-222. doi: 10.1038/ng1095-219.

Achouri-Rassas, A., Ben Ali, N., Fray, S., Hadj Fredj, S., Kechaou, M., Zakraoui, N.O., et al. (2015). Novel presenilin 1 mutation (p.I83T) in Tunisian family with early-onset Alzheimer's disease. *Neurobiol Aging* 36(10)**,** 2904.e2909-2911. doi: 10.1016/j.neurobiolaging.2015.06.007.

Alberici, A., Bonato, C., Borroni, B., Cotelli, M., Mattioli, F., Binetti, G., et al. (2007). Dementia, delusions and seizures: storage disease or genetic AD? *Eur J Neurol* 14(9)**,** 1057-1059. doi: 10.1111/j.1468-1331.2007.01664.x.

Aldudo, J., Bullido, M.J., Arbizu, T., Oliva, R., and Valdivieso, F. (1998). Identification of a novel mutation (Leu282Arg) of the human presenilin 1 gene in Alzheimer's disease. *Neurosci Lett* 240(3)**,** 174-176. doi: 10.1016/s0304-3940(97)00950-6.

Aldudo, J., Bullido, M.J., and Valdivieso, F. (1999). DGGE method for the mutational analysis of the coding and proximal promoter regions of the Alzheimer's disease presenilin-1 gene: two novel mutations. *Hum Mutat* 14(5)**,** 433-439. doi: 10.1002/(sici)1098-1004(199911)14:5<433::Aid-humu10>3.0.Co;2-k.

An, S.S., Bagyinszky, E., Kim, H.R., Seok, J.W., Shin, H.W., Bae, S., et al. (2016a). Novel PSEN1 G209A mutation in early-onset Alzheimer dementia supported by structural prediction. *BMC Neurol* 16**,** 71. doi: 10.1186/s12883-016-0591-6.

An, S.S., Park, S.A., Bagyinszky, E., Bae, S.O., Kim, Y.J., Im, J.Y., et al. (2016b). A genetic screen of the mutations in the Korean patients with early-onset Alzheimer's disease. *Clin Interv Aging* 11**,** 1817-1822. doi: 10.2147/cia.S116724.

Ancolio, K., Dumanchin, C., Barelli, H., Warter, J.M., Brice, A., Campion, D., et al. (1999). Unusual phenotypic alteration of beta amyloid precursor protein (betaAPP) maturation by a new Val-715 --> Met betaAPP-770 mutation responsible for probable early-onset Alzheimer's disease. *Proc Natl Acad Sci U S A* 96(7)**,** 4119-4124. doi: 10.1073/pnas.96.7.4119.

Anheim, M., Hannequin, D., Boulay, C., Martin, C., Campion, D., and Tranchant, C. (2007). Ataxic variant of Alzheimer's disease caused by Pro117Ala PSEN1 mutation. *J Neurol Neurosurg Psychiatry* 78(12)**,** 1414-1415. doi: 10.1136/jnnp.2007.123026.

Antonell, A., Balasa, M., Oliva, R., Lladó, A., Bosch, B., Fabregat, N., et al. (2011). A novel PSEN1 gene mutation (L235R) associated with familial early-onset Alzheimer's disease. *Neurosci Lett* 496(1)**,** 40-42. doi: 10.1016/j.neulet.2011.03.084.

Arango, D., Cruts, M., Torres, O., Backhovens, H., Serrano, M.L., Villareal, E., et al. (2001). Systematic genetic study of Alzheimer disease in Latin America: mutation frequencies of the amyloid beta precursor protein and presenilin genes in Colombia. *Am J Med Genet* 103(2)**,** 138-143. doi: 10.1002/1096-8628(20011001)103:2<138::aid-ajmg1529>3.0.co;2-8.

Arber, C., Toombs, J., Lovejoy, C., Ryan, N.S., Paterson, R.W., Willumsen, N., et al. (2020). Familial Alzheimer's disease patient-derived neurons reveal distinct mutation-specific effects on amyloid beta. *Mol Psychiatry* 25(11)**,** 2919-2931. doi: 10.1038/s41380-019-0410-8.

Armstrong, J., Boada, M., Rey, M.J., Vidal, N., and Ferrer, I. (2004). Familial Alzheimer disease associated with A713T mutation in APP. *Neurosci Lett* 370(2-3)**,** 241-243. doi: 10.1016/j.neulet.2004.08.026.

Assini, A., Terreni, L., Borghi, R., Giliberto, L., Piccini, A., Loqui, D., et al. (2003). Pure spastic paraparesis associated with a novel presenilin 1 R278K mutation. *Neurology* 60(1)**,** 150. doi: 10.1212/01.wnl.0000040252.43269.83.

Ataka, S., Tomiyama, T., Takuma, H., Yamashita, T., Shimada, H., Tsutada, T., et al. (2004). A novel presenilin-1 mutation (Leu85Pro) in early-onset Alzheimer disease with spastic paraparesis. *Arch Neurol* 61(11)**,** 1773-1776. doi: 10.1001/archneur.61.11.1773.

Bagyinszky, E., Kang, M.J., Van Giau, V., Shim, K., Pyun, J.M., Suh, J., et al. (2019). Novel amyloid precursor protein mutation, Val669Leu ("Seoul APP"), in a Korean patient with early-onset Alzheimer's disease. *Neurobiol Aging* 84**,** 236.e231-236.e237. doi: 10.1016/j.neurobiolaging.2019.08.026.

Bagyinszky, E., Park, S.A., Kim, H.J., Choi, S.H., An, S.S., and Kim, S.Y. (2016). PSEN1 L226F mutation in a patient with early-onset Alzheimer's disease in Korea. *Clin Interv Aging* 11**,** 1433-1440. doi: 10.2147/cia.S111821.

Balbín, M., Abrahamson, M., Gustafson, L., Nilsson, K., Brun, A., and Grubb, A. (1992). A novel mutation in the beta-protein coding region of the amyloid beta-protein precursor (APP) gene. *Hum Genet* 89(5)**,** 580-582. doi: 10.1007/bf00219191.

Bernardi, L., Tomaino, C., Anfossi, M., Gallo, M., Geracitano, S., Costanzo, A., et al. (2009). Novel PSEN1 and PGRN mutations in early-onset familial frontotemporal dementia. *Neurobiol Aging* 30(11)**,** 1825-1833. doi: 10.1016/j.neurobiolaging.2008.01.005.

Bernardi, L., Tomaino, C., Anfossi, M., Gallo, M., Geracitano, S., Puccio, G., et al. (2008). Late onset familial Alzheimer's disease: novel presenilin 2 mutation and PS1 E318G polymorphism. *J Neurol* 255(4)**,** 604-606. doi: 10.1007/s00415-008-0764-3.

Bertoli Avella, A.M., Marcheco Teruel, B., Llibre Rodriguez, J.J., Gomez Viera, N., Borrajero Martinez, I., Severijnen, E.A., et al. (2002). A novel presenilin 1 mutation (L174 M) in a large Cuban family with early onset Alzheimer disease. *Neurogenetics* 4(2)**,** 97-104. doi: 10.1007/s10048-002-0136-6.

Besançon, R., Lorenzi, A., Cruts, M., Radawiec, S., Sturtz, F., Broussolle, E., et al. (1998). Missense mutation in exon 11 (Codon 378) of the presenilin-1 gene in a French family with early-onset Alzheimer's disease and transmission study by mismatch enhanced allele specific amplification. Mutations in brief no. 141. Online. besancon@rockefeller1.univ.lyon1.fr. *Hum Mutat* 11(6)**,** 481. doi: 10.1002/(sici)1098-1004(1998)11:6<481::Aid-humu12>3.0.Co;2-q.

Bialopiotrowicz, E., Szybinska, A., Kuzniewska, B., Buizza, L., Uberti, D., Kuznicki, J., et al. (2012). Highly pathogenic Alzheimer's disease presenilin 1 P117R mutation causes a specific increase in p53 and p21 protein levels and cell cycle dysregulation in human lymphocytes. *J Alzheimers Dis* 32(2)**,** 397-415. doi: 10.3233/jad-2012-121129.

Binetti, G., Signorini, S., Squitti, R., Alberici, A., Benussi, L., Cassetta, E., et al. (2003). Atypical dementia associated with a novel presenilin-2 mutation. *Ann Neurol* 54(6)**,** 832-836. doi: 10.1002/ana.10760.

Blanco, J.A., Alonso, A., Blanco, J., Rojo, E., Tellería, J.J., Torres, M.A., et al. (2019). Novel presenilin 1 mutation (p.Thr-Pro116-117Ser-Thr) in a Spanish family with early-onset Alzheimer's disease. *Neurobiol Aging* 84**,** 238.e219-238.e224. doi: 10.1016/j.neurobiolaging.2019.05.012.

Blauwendraat, C., Wilke, C., Jansen, I.E., Schulte, C., Simón-Sánchez, J., Metzger, F.G., et al. (2016). Pilot whole-exome sequencing of a German early-onset Alzheimer's disease cohort reveals a substantial frequency of PSEN2 variants. *Neurobiol Aging* 37**,** 208.e211-208.e217. doi: 10.1016/j.neurobiolaging.2015.09.016.

Blauwendraat, C., Wilke, C., Simón-Sánchez, J., Jansen, I.E., Reifschneider, A., Capell, A., et al. (2018). The wide genetic landscape of clinical frontotemporal dementia: systematic combined sequencing of 121 consecutive subjects. *Genet Med* 20(2)**,** 240-249. doi: 10.1038/gim.2017.102.

Brouwers, N., Sleegers, K., and Van Broeckhoven, C. (2008). Molecular genetics of Alzheimer's disease: an update. *Ann Med* 40(8)**,** 562-583. doi: 10.1080/07853890802186905.

Bugiani, O., Giaccone, G., Rossi, G., Mangieri, M., Capobianco, R., Morbin, M., et al. (2010). Hereditary cerebral hemorrhage with amyloidosis associated with the E693K mutation of APP. *Arch Neurol* 67(8)**,** 987-995. doi: 10.1001/archneurol.2010.178.

Butler, R., Beattie, B.L., Thong, U.P., Dwosh, E., Guimond, C., Feldman, H.H., et al. (2010). A novel PS1 gene mutation in a large Aboriginal kindred. *Can J Neurol Sci* 37(3)**,** 359-364. doi: 10.1017/s0317167100010258.

Campion, D., Brice, A., Dumanchin, C., Puel, M., Baulac, M., De La Sayette, V., et al. (1996). A novel presenilin 1 mutation resulting in familial Alzheimer's disease with an onset age of 29 years. *Neuroreport* 7(10)**,** 1582-1584. doi: 10.1097/00001756-199607080-00009.

Campion, D., Brice, A., Hannequin, D., Tardieu, S., Dubois, B., Calenda, A., et al. (1995a). A large pedigree with early-onset Alzheimer's disease: clinical, neuropathologic, and genetic characterization. *Neurology* 45(1)**,** 80-85. doi: 10.1212/wnl.45.1.80.

Campion, D., Dumanchin, C., Hannequin, D., Dubois, B., Belliard, S., Puel, M., et al. (1999). Early-onset autosomal dominant Alzheimer disease: prevalence, genetic heterogeneity, and mutation spectrum. *Am J Hum Genet* 65(3)**,** 664-670. doi: 10.1086/302553.

Campion, D., Flaman, J.M., Brice, A., Hannequin, D., Dubois, B., Martin, C., et al. (1995b). Mutations of the presenilin I gene in families with early-onset Alzheimer's disease. *Hum Mol Genet* 4(12)**,** 2373-2377. doi: 10.1093/hmg/4.12.2373.

Carecchio, M., Picillo, M., Valletta, L., Elia, A.E., Haack, T.B., Cozzolino, A., et al. (2017). Rare causes of early-onset dystonia-parkinsonism with cognitive impairment: a de novo PSEN-1 mutation. *Neurogenetics* 18(3)**,** 175-178. doi: 10.1007/s10048-017-0518-4.

Carter, D.A., Desmarais, E., Bellis, M., Campion, D., Clerget-Darpoux, F., Brice, A., et al. (1992). More missense in amyloid gene. *Nat Genet* 2(4)**,** 255-256. doi: 10.1038/ng1292-255.

Ch'ng, G.S., An, S.S., Bae, S.O., Bagyinszky, E., and Kim, S. (2015). Identification of two novel mutations, PSEN1 E280K and PRNP G127S, in a Malaysian family. *Neuropsychiatr Dis Treat* 11**,** 2315-2322. doi: 10.2147/ndt.S86334.

Chen, W.T., Hong, C.J., Lin, Y.T., Chang, W.H., Huang, H.T., Liao, J.Y., et al. (2012). Amyloid-beta (Aβ) D7H mutation increases oligomeric Aβ42 and alters properties of Aβ-zinc/copper assemblies. *PLoS One* 7(4)**,** e35807. doi: 10.1371/journal.pone.0035807.

Chen, W.T., Hsieh, Y.F., Huang, Y.J., Lin, C.C., Lin, Y.T., Liu, Y.C., et al. (2015). G206D Mutation of Presenilin-1 Reduces Pen2 Interaction, Increases Aβ42/Aβ40 Ratio and Elevates ER Ca(2+) Accumulation. *Mol Neurobiol* 52(3)**,** 1835-1849. doi: 10.1007/s12035-014-8969-1.

Church, A., Prescott, J., Lillis, S., Rees, J., Chance, P., Williamson, K., et al. (2011). A novel presenilin 1 mutation, I202F occurring at a previously predicted pathogenic site causing autosomal dominant Alzheimer's disease. *Neurobiol Aging* 32(3)**,** 556.e551-552. doi: 10.1016/j.neurobiolaging.2010.09.030.

Citron, M., Vigo-Pelfrey, C., Teplow, D.B., Miller, C., Schenk, D., Johnston, J., et al. (1994). Excessive production of amyloid beta-protein by peripheral cells of symptomatic and presymptomatic patients carrying the Swedish familial Alzheimer disease mutation. *Proc Natl Acad Sci U S A* 91(25)**,** 11993-11997. doi: 10.1073/pnas.91.25.11993.

Clarimon J, Guerreiro R, Lleó A, Guardia-Laguarta C, Blesa R, Gómez-Isla T, et al. (2008).P3-213: Genetic screening in a large cohort of early-onset Alzheimer's disease patients from Spain: Novel mutations in the amyloid precursor protein and presenilines. Alzheimer's & Dementia;4:T583.

Colacicco, A.M., Panza, F., Basile, A.M., Solfrizzi, V., Capurso, C., D'Introno, A., et al. (2002). F175S change and a novel polymorphism in presenilin-1 gene in late-onset familial Alzheimer's disease. *Eur Neurol* 47(4)**,** 209-213. doi: 10.1159/000057901.

Coleman, P., Kurlan, R., Crook, R., Werner, J., and Hardy, J. (2004). A new presenilin Alzheimer's disease case confirms the helical alignment of pathogenic mutations in transmembrane domain 5. *Neurosci Lett* 364(3)**,** 139-140. doi: 10.1016/j.neulet.2004.04.030.

Coppola, C., Saracino, D., Oliva, M., Cipriano, L., Puoti, G., Pappatà, S., et al. (2021). Singular cases of Alzheimer's disease disclose new and old genetic "acquaintances". *Neurol Sci* 42(5)**,** 2021-2029. doi: 10.1007/s10072-020-04774-y.

Couthouis, J., Raphael, A.R., Daneshjou, R., and Gitler, A.D. (2014). Targeted exon capture and sequencing in sporadic amyotrophic lateral sclerosis. *PLoS Genet* 10(10)**,** e1004704. doi: 10.1371/journal.pgen.1004704.

Croes, E.A., Theuns, J., Houwing-Duistermaat, J.J., Dermaut, B., Sleegers, K., Roks, G., et al. (2004). Octapeptide repeat insertions in the prion protein gene and early onset dementia. *J Neurol Neurosurg Psychiatry* 75(8)**,** 1166-1170. doi: 10.1136/jnnp.2003.020198.

Crook, R., Ellis, R., Shanks, M., Thal, L.J., Perez-Tur, J., Baker, M., et al. (1997). Early-onset Alzheimer's disease with a presenilin-1 mutation at the site corresponding to the Volga German presenilin-2 mutation. *Ann Neurol* 42(1)**,** 124-128. doi: 10.1002/ana.410420121.

Crook, R., Verkkoniemi, A., Perez-Tur, J., Mehta, N., Baker, M., Houlden, H., et al. (1998). A variant of Alzheimer's disease with spastic paraparesis and unusual plaques due to deletion of exon 9 of presenilin 1. *Nat Med* 4(4)**,** 452-455. doi: 10.1038/nm0498-452.

Cruts, M., Backhovens, H., Wang, S.Y., Van Gassen, G., Theuns, J., De Jonghe, C.D., et al. (1995). Molecular genetic analysis of familial early-onset Alzheimer's disease linked to chromosome 14q24.3. *Hum Mol Genet* 4(12)**,** 2363-2371. doi: 10.1093/hmg/4.12.2363.

Cruts, M., Dermaut, B., Rademakers, R., Van den Broeck, M., Stögbauer, F., and Van Broeckhoven, C. (2003). Novel APP mutation V715A associated with presenile Alzheimer's disease in a German family. *J Neurol* 250(11)**,** 1374-1375. doi: 10.1007/s00415-003-0182-5.

Cruts, M., van Duijn, C.M., Backhovens, H., Van den Broeck, M., Wehnert, A., Serneels, S., et al. (1998). Estimation of the genetic contribution of presenilin-1 and -2 mutations in a population-based study of presenile Alzheimer disease. *Hum Mol Genet* 7(1)**,** 43-51. doi: 10.1093/hmg/7.1.43.

Day, G.S., Musiek, E.S., Roe, C.M., Norton, J., Goate, A.M., Cruchaga, C., et al. (2016). Phenotypic Similarities Between Late-Onset Autosomal Dominant and Sporadic Alzheimer Disease: A Single-Family Case-Control Study. *JAMA Neurol* 73(9)**,** 1125-1132. doi: 10.1001/jamaneurol.2016.1236.

De Jonghe, C., Cruts, M., Rogaeva, E.A., Tysoe, C., Singleton, A., Vanderstichele, H., et al. (1999). Aberrant splicing in the presenilin-1 intron 4 mutation causes presenile Alzheimer's disease by increased Abeta42 secretion. *Hum Mol Genet* 8(8)**,** 1529-1540. doi: 10.1093/hmg/8.8.1529.

De Jonghe, C., Esselens, C., Kumar-Singh, S., Craessaerts, K., Serneels, S., Checler, F., et al. (2001). Pathogenic APP mutations near the gamma-secretase cleavage site differentially affect Abeta secretion and APP C-terminal fragment stability. *Hum Mol Genet* 10(16)**,** 1665-1671. doi: 10.1093/hmg/10.16.1665.

Deng, B., Lian, Y., Wang, X., Zeng, F., Jiao, B., Wang, Y.R., et al. (2014). Identification of a novel mutation in the presenilin 1 gene in a Chinese Alzheimer's disease family. *Neurotox Res* 26(3)**,** 211-215. doi: 10.1007/s12640-014-9462-3.

Dermaut, B., Kumar-Singh, S., De Jonghe, C., Cruts, M., Löfgren, A., Lübke, U., et al. (2001). Cerebral amyloid angiopathy is a pathogenic lesion in Alzheimer's disease due to a novel presenilin 1 mutation. *Brain* 124(Pt 12)**,** 2383-2392. doi: 10.1093/brain/124.12.2383.

Dermaut, B., Kumar-Singh, S., Engelborghs, S., Theuns, J., Rademakers, R., Saerens, J., et al. (2004). A novel presenilin 1 mutation associated with Pick's disease but not beta-amyloid plaques. *Ann Neurol* 55(5)**,** 617-626. doi: 10.1002/ana.20083.

Devi, G., Fotiou, A., Jyrinji, D., Tycko, B., DeArmand, S., Rogaeva, E., et al. (2000). Novel presenilin 1 mutations associated with early onset of dementia in a family with both early-onset and late-onset Alzheimer disease. *Arch Neurol* 57(10)**,** 1454-1457. doi: 10.1001/archneur.57.10.1454.

Di Fede, G., Catania, M., Morbin, M., Rossi, G., Suardi, S., Mazzoleni, G., et al. (2009). A recessive mutation in the APP gene with dominant-negative effect on amyloidogenesis. *Science* 323(5920)**,** 1473-1477. doi: 10.1126/science.1168979.

Dintchov Traykov, L., Mehrabian, S., Van den Broeck, M., Radoslavova Raycheva, M., Cruts, M., Kirilova Jordanova, A., et al. (2009). Novel PSEN1 mutation in a Bulgarian patient with very early-onset Alzheimer's disease, spastic paraparesis, and extrapyramidal signs. *Am J Alzheimers Dis Other Demen* 24(5)**,** 404-407. doi: 10.1177/1533317509341464.

Dobricic, V., Stefanova, E., Jankovic, M., Gurunlian, N., Novakovic, I., Hardy, J., et al. (2012). Genetic testing in familial and young-onset Alzheimer's disease: mutation spectrum in a Serbian cohort. *Neurobiol Aging* 33(7)**,** 1481.e1487-1412. doi: 10.1016/j.neurobiolaging.2011.12.007.

Dolzhanskaya, N., Gonzalez, M.A., Sperziani, F., Stefl, S., Messing, J., Wen, G.Y., et al. (2014). A novel p.Leu(381)Phe mutation in presenilin 1 is associated with very early onset and unusually fast progressing dementia as well as lysosomal inclusions typically seen in Kufs disease. *J Alzheimers Dis* 39(1)**,** 23-27. doi: 10.3233/jad-131340.

Dong, J., Qin, W., Wei, C., Tang, Y., Wang, Q., and Jia, J. (2017). A Novel PSEN1 K311R Mutation Discovered in Chinese Families with Late-Onset Alzheimer's Disease Affects Amyloid-β Production and Tau Phosphorylation. *J Alzheimers Dis* 57(2)**,** 613-623. doi: 10.3233/jad-161188.

Dowjat, W.K., Kuchna, I., Wisniewski, T., and Wegiel, J. (2004). A novel highly pathogenic Alzheimer presenilin-1 mutation in codon 117 (Pro117Ser): Comparison of clinical, neuropathological and cell culture phenotypes of Pro117Leu and Pro117Ser mutations. *J Alzheimers Dis* 6(1)**,** 31-43. doi: 10.3233/jad-2004-6105.

Dumanchin, C., Brice, A., Campion, D., Hannequin, D., Martin, C., Moreau, V., et al. (1998). De novo presenilin 1 mutations are rare in clinically sporadic, early onset Alzheimer's disease cases. French Alzheimer's Disease Study Group. *J Med Genet* 35(8)**,** 672-673. doi: 10.1136/jmg.35.8.672.

Dumanchin, C., Tournier, I., Martin, C., Didic, M., Belliard, S., Carlander, B., et al. (2006). Biological effects of four PSEN1 gene mutations causing Alzheimer disease with spastic paraparesis and cotton wool plaques. *Hum Mutat* 27(10)**,** 1063. doi: 10.1002/humu.9458.

Eckman, C.B., Mehta, N.D., Crook, R., Perez-tur, J., Prihar, G., Pfeiffer, E., et al. (1997). A new pathogenic mutation in the APP gene (I716V) increases the relative proportion of A beta 42(43). *Hum Mol Genet* 6(12)**,** 2087-2089. doi: 10.1093/hmg/6.12.2087.

Edwards-Lee, T., Ringman, J.M., Chung, J., Werner, J., Morgan, A., St George Hyslop, P., et al. (2005). An African American family with early-onset Alzheimer disease and an APP (T714I) mutation. *Neurology* 64(2)**,** 377-379. doi: 10.1212/01.Wnl.0000149761.70566.3e.

Edwards-Lee, T., Wen, J., Bell, J., Hardy, J., Chung, J., and Momeni, P. (2006). A presenilin-1 mutation (T245P) in transmembrane domain 6 causes early onset Alzheimer's disease. *Neurosci Lett* 398(3)**,** 251-252. doi: 10.1016/j.neulet.2006.01.006.

El Bitar, F., Qadi, N., Al Rajeh, S., Majrashi, A., Abdulaziz, S., Majrashi, N., et al. (2019). Genetic Study of Alzheimer's Disease in Saudi Population. *J Alzheimers Dis* 67(1)**,** 231-242. doi: 10.3233/jad-180415.

El Kadmiri, N., Zaid, N., Zaid, Y., Tadevosyan, A., Hachem, A., Dubé, M.P., et al. (2014). Novel presenilin mutations within Moroccan patients with Early-Onset Alzheimer's Disease. *Neuroscience* 269**,** 215-222. doi: 10.1016/j.neuroscience.2014.03.052.

Ezquerra, M., Carnero, C., Blesa, R., Gelpí, J.L., Ballesta, F., and Oliva, R. (1999). A presenilin 1 mutation (Ser169Pro) associated with early-onset AD and myoclonic seizures. *Neurology* 52(3)**,** 566-570. doi: 10.1212/wnl.52.3.566.

Ezquerra, M., Carnero, C., Blesa, R., and Oliva, R. (2000). A novel presenilin 1 mutation (Leu166Arg) associated with early-onset Alzheimer disease. *Arch Neurol* 57(4)**,** 485-488. doi: 10.1001/archneur.57.4.485.

Ezquerra, M., Lleó, A., Castellví, M., Queralt, R., Santacruz, P., Pastor, P., et al. (2003). A novel mutation in the PSEN2 gene (T430M) associated with variable expression in a family with early-onset Alzheimer disease. *Arch Neurol* 60(8)**,** 1149-1151. doi: 10.1001/archneur.60.8.1149.

Fang, B., Jia, L., and Jia, J. (2006). Chinese Presenilin-1 V97L mutation enhanced Abeta42 levels in SH-SY5Y neuroblastoma cells. *Neurosci Lett* 406(1-2)**,** 33-37. doi: 10.1016/j.neulet.2006.06.072.

Fang, B.Y., and Jia, J.P. (2008). Human neuroblastoma cells transfected with two Chinese presenilin 1 mutations are sensitized to trophic factor withdrawal and protected by insulin-like growth factor-1. *Chin Med J (Engl)* 121(10)**,** 910-915.

Farlow M, Murrell J, Unverzagt F, Phillips M, Takao M, Hulette C, et al. (2002). Familial Alzheimer's Disease with Spastic Paraparesis Associated with a Mutation at Codon 261 of the Presenilin 1 Gene. p. 53-60.

Fidani, L., Rooke, K., Chartier-Harlin, M.C., Hughes, D., Tanzi, R., Mullan, M., et al. (1992). Screening for mutations in the open reading frame and promoter of the beta-amyloid precursor protein gene in familial Alzheimer's disease: identification of a further family with APP717 Val-->Ile. *Hum Mol Genet* 1(3)**,** 165-168. doi: 10.1093/hmg/1.3.165.

Finckh, U., Alberici, A., Antoniazzi, M., Benussi, L., Fedi, V., Giannini, C., et al. (2000a). Variable expression of familial Alzheimer disease associated with presenilin 2 mutation M239I. *Neurology* 54(10)**,** 2006-2008. doi: 10.1212/wnl.54.10.2006.

Finckh, U., Kuschel, C., Anagnostouli, M., Patsouris, E., Pantes, G.V., Gatzonis, S., et al. (2005). Novel mutations and repeated findings of mutations in familial Alzheimer disease. *Neurogenetics* 6(2)**,** 85-89. doi: 10.1007/s10048-005-0211-x.

Finckh, U., Müller-Thomsen, T., Mann, U., Eggers, C., Marksteiner, J., Meins, W., et al. (2000b). High prevalence of pathogenic mutations in patients with early-onset dementia detected by sequence analyses of four different genes. *Am J Hum Genet* 66(1)**,** 110-117. doi: 10.1086/302702.

Forsell, C., Froelich, S., Axelman, K., Vestling, M., Cowburn, R.F., Lilius, L., et al. (1997). A novel pathogenic mutation (Leu262Phe) found in the presenilin 1 gene in early-onset Alzheimer's disease. *Neurosci Lett* 234(1)**,** 3-6. doi: 10.1016/s0304-3940(97)00603-4.

Fray, S., Rassas, A., Messaoud, T., and Belal, S. (2020). Refractory epilepsy in PSEN 1 mutation (I83T). *Neurocase* 26(3)**,** 167-170. doi: 10.1080/13554794.2020.1747632.

Furuya, H., Yasuda, M., Terasawa, K.J., Tanaka, K., Murai, H., Kira, J., et al. (2003). A novel mutation (L250V) in the presenilin 1 gene in a Japanese familial Alzheimer's disease with myoclonus and generalized convulsion. *J Neurol Sci* 209(1-2)**,** 75-77. doi: 10.1016/s0022-510x(02)00466-5.

Gallo, M., Frangipane, F., Cupidi, C., De Bartolo, M., Turone, S., Ferrari, C., et al. (2017). The novel PSEN1 M84V mutation associated to frontal dysexecutive syndrome, spastic paraparesis, and cerebellar atrophy in a dominant Alzheimer's disease family. *Neurobiol Aging* 56**,** 213.e217-213.e212. doi: 10.1016/j.neurobiolaging.2017.04.017.

Gallo, M., Marcello, N., Curcio, S.A., Colao, R., Geracitano, S., Bernardi, L., et al. (2011). A novel pathogenic PSEN1 mutation in a family with Alzheimer's disease: phenotypical and neuropathological features. *J Alzheimers Dis* 25(3)**,** 425-431. doi: 10.3233/jad-2011-110185.

Gao, Y., Ren, R.J., Zhong, Z.L., Dammer, E., Zhao, Q.H., Shan, S., et al. (2019). Mutation profile of APP, PSEN1, and PSEN2 in Chinese familial Alzheimer's disease. *Neurobiol Aging* 77**,** 154-157. doi: 10.1016/j.neurobiolaging.2019.01.018.

Gatto, E.M., Rojas, G.J., Nemirovsky, S.I., Da Prat, G., Persi, G., Cesarini, M., et al. (2020). A novel mutation in PSEN1 (p.Arg41Ser) in an Argentinian woman with early onset Parkinsonism. *Parkinsonism Relat Disord* 77**,** 21-25. doi: 10.1016/j.parkreldis.2020.06.005.

Ghidoni, R., Albertini, V., Squitti, R., Paterlini, A., Bruno, A., Bernardini, S., et al. (2009). Novel T719P AbetaPP mutation unbalances the relative proportion of amyloid-beta peptides. *J Alzheimers Dis* 18(2)**,** 295-303. doi: 10.3233/jad-2009-1142.

Giau, V.V., Wang, M.J., Bagyinszky, E., Youn, Y.C., An, S.S.A., and Kim, S. (2018). Novel PSEN1 p.Gly417Ala mutation in a Korean patient with early-onset Alzheimer's disease with parkinsonism. *Neurobiol Aging* 72**,** 188.e113-188.e117. doi: 10.1016/j.neurobiolaging.2018.08.003.

Goate, A., Chartier-Harlin, M.C., Mullan, M., Brown, J., Crawford, F., Fidani, L., et al. (1991). Segregation of a missense mutation in the amyloid precursor protein gene with familial Alzheimer's disease. *Nature* 349(6311)**,** 704-706. doi: 10.1038/349704a0.

Godbolt, A.K., Beck, J.A., Collinge, J., Garrard, P., Warren, J.D., Fox, N.C., et al. (2004). A presenilin 1 R278I mutation presenting with language impairment. *Neurology* 63(9)**,** 1702-1704. doi: 10.1212/01.wnl.0000143060.98164.1a.

Godbolt, A.K., Beck, J.A., Collinge, J.C., Cipolotti, L., Fox, N.C., and Rossor, M.N. (2006). A second family with familial AD and the V717L APP mutation has a later age at onset. *Neurology* 66(4)**,** 611-612. doi: 10.1212/01.Wnl.0000197791.53828.2c.

Goldman, J.S., Reed, B., Gearhart, R., Kramer, J.H., and Miller, B.L. (2002). Very early-onset familial Alzheimer's disease: a novel presenilin 1 mutation. *Int J Geriatr Psychiatry* 17(7)**,** 649-651. doi: 10.1002/gps.657.

Gómez-Isla, T., Wasco, W., Pettingell, W.P., Gurubhagavatula, S., Schmidt, S.D., Jondro, P.D., et al. (1997). A novel presenilin-1 mutation: increased beta-amyloid and neurofibrillary changes. *Ann Neurol* 41(6)**,** 809-813. doi: 10.1002/ana.410410618.

Gómez-Tortosa, E., Barquero, S., Barón, M., Gil-Neciga, E., Castellanos, F., Zurdo, M., et al. (2010). Clinical-genetic correlations in familial Alzheimer's disease caused by presenilin 1 mutations. *J Alzheimers Dis* 19(3)**,** 873-884. doi: 10.3233/jad-2010-1292.

Grabowski, T.J., Cho, H.S., Vonsattel, J.P., Rebeck, G.W., and Greenberg, S.M. (2001). Novel amyloid precursor protein mutation in an Iowa family with dementia and severe cerebral amyloid angiopathy. *Ann Neurol* 49(6)**,** 697-705. doi: 10.1002/ana.1009.

Greenberg, S.M., Shin, Y., Grabowski, T.J., Cooper, G.E., Rebeck, G.W., Iglesias, S., et al. (2003). Hemorrhagic stroke associated with the Iowa amyloid precursor protein mutation. *Neurology* 60(6)**,** 1020-1022. doi: 10.1212/01.wnl.0000050140.10044.a8.

Guardia-Laguarta, C., Pera, M., Clarimón, J., Molinuevo, J.L., Sánchez-Valle, R., Lladó, A., et al. (2010). Clinical, neuropathologic, and biochemical profile of the amyloid precursor protein I716F mutation. *J Neuropathol Exp Neurol* 69(1)**,** 53-59. doi: 10.1097/NEN.0b013e3181c6b84d.

Guerreiro, R.J., Baquero, M., Blesa, R., Boada, M., Brás, J.M., Bullido, M.J., et al. (2010). Genetic screening of Alzheimer's disease genes in Iberian and African samples yields novel mutations in presenilins and APP. *Neurobiol Aging* 31(5)**,** 725-731. doi: 10.1016/j.neurobiolaging.2008.06.012.

Guo, J., Wei, J., Liao, S., Wang, L., Jiang, H., and Tang, B. (2010). A novel presenilin 1 mutation (Ser169del) in a Chinese family with early-onset Alzheimer's disease. *Neurosci Lett* 468(1)**,** 34-37. doi: 10.1016/j.neulet.2009.10.055.

Guven, G., Erginel-Unaltuna, N., Samanci, B., Gulec, C., Hanagasi, H., and Bilgic, B. (2019). A patient with early-onset Alzheimer's disease with a novel PSEN1 p.Leu424Pro mutation. *Neurobiol Aging* 84**,** 238.e231-238.e234. doi: 10.1016/j.neurobiolaging.2019.05.014.

Hamaguchi, T., Morinaga, A., Tsukie, T., Kuwano, R., and Yamada, M. (2009). A novel presenilin 1 mutation (L282F) in familial Alzheimer's disease. *J Neurol* 256(9)**,** 1575-1577. doi: 10.1007/s00415-009-5154-y.

Han, L.H., Xue, Y.Y., Zheng, Y.C., Li, X.Y., Lin, R.R., Wu, Z.Y., et al. (2020). Genetic Analysis of Chinese Patients with Early-Onset Dementia Using Next-Generation Sequencing. *Clin Interv Aging* 15**,** 1831-1839. doi: 10.2147/cia.S271222.

Hattori, S., Sakuma, K., Wakutani, Y., Wada, K., Shimoda, M., Urakami, K., et al. (2004). A novel presenilin 1 mutation (Y154N) in a patient with early onset Alzheimer's disease with spastic paraparesis. *Neurosci Lett* 368(3)**,** 319-322. doi: 10.1016/j.neulet.2004.07.057.

Hausner, L., Tschäpe, J.A., Schmitt, H.P., Hentschel, F., Hartmann, T., and Frölich, L. (2014). Clinical characterization of a presenilin 1 mutation (F177S) in a family with very early-onset Alzheimer's disease in the third decade of life. *Alzheimers Dement* 10(2)**,** e27-39. doi: 10.1016/j.jalz.2013.02.006.

Heckmann, J.M., Low, W.C., de Villiers, C., Rutherfoord, S., Vorster, A., Rao, H., et al. (2004). Novel presenilin 1 mutation with profound neurofibrillary pathology in an indigenous Southern African family with early-onset Alzheimer's disease. *Brain* 127(Pt 1)**,** 133-142. doi: 10.1093/brain/awh009.

Hendriks, L., van Duijn, C.M., Cras, P., Cruts, M., Van Hul, W., van Harskamp, F., et al. (1992). Presenile dementia and cerebral haemorrhage linked to a mutation at codon 692 of the beta-amyloid precursor protein gene. *Nat Genet* 1(3)**,** 218-221. doi: 10.1038/ng0692-218.

Herl, L., Thomas, A.V., Lill, C.M., Banks, M., Deng, A., Jones, P.B., et al. (2009). Mutations in amyloid precursor protein affect its interactions with presenilin/gamma-secretase. *Mol Cell Neurosci* 41(2)**,** 166-174. doi: 10.1016/j.mcn.2009.02.008.

Hooli, B.V., Kovacs-Vajna, Z.M., Mullin, K., Blumenthal, M.A., Mattheisen, M., Zhang, C., et al. (2014). Rare autosomal copy number variations in early-onset familial Alzheimer's disease. *Mol Psychiatry* 19(6)**,** 676-681. doi: 10.1038/mp.2013.77.

Houlden, H., Baker, M., McGowan, E., Lewis, P., Hutton, M., Crook, R., et al. (2000). Variant Alzheimer's disease with spastic paraparesis and cotton wool plaques is caused by PS-1 mutations that lead to exceptionally high amyloid-beta concentrations. *Ann Neurol* 48(5)**,** 806-808.

Houlden, H., Crook, R., Dolan, R.J., McLaughlin, J., Revesz, T., and Hardy, J. (2001). A novel presenilin mutation (M233V) causing very early onset Alzheimer's disease with Lewy bodies. *Neurosci Lett* 313(1-2)**,** 93-95. doi: 10.1016/s0304-3940(01)02254-6.

Hsu, S., Gordon, B.A., Hornbeck, R., Norton, J.B., Levitch, D., Louden, A., et al. (2018). Discovery and validation of autosomal dominant Alzheimer's disease mutations. *Alzheimers Res Ther* 10(1)**,** 67. doi: 10.1186/s13195-018-0392-9.

Hsu, S., Pimenova, A.A., Hayes, K., Villa, J.A., Rosene, M.J., Jere, M., et al. (2020). Systematic validation of variants of unknown significance in APP, PSEN1 and PSEN2. *Neurobiol Dis* 139**,** 104817. doi: 10.1016/j.nbd.2020.104817.

Hutton, M., Busfield, F., Wragg, M., Crook, R., Perez-Tur, J., Clark, R.F., et al. (1996). Complete analysis of the presenilin 1 gene in early onset Alzheimer's disease. *Neuroreport* 7(3)**,** 801-805. doi: 10.1097/00001756-199602290-00029.

Ikeda, M., Sharma, V., Sumi, S.M., Rogaeva, E.A., Poorkaj, P., Sherrington, R., et al. (1996). The clinical phenotype of two missense mutations in the presenilin I gene in Japanese patients. *Ann Neurol* 40(6)**,** 912-917. doi: 10.1002/ana.410400614.

Ikeda, M., Yonemura, K., Kakuda, S., Tashiro, Y., Fujita, Y., Takai, E., et al. (2013). Cerebrospinal fluid levels of phosphorylated tau and Aβ1-38/Aβ1-40/Aβ1-42 in Alzheimer's disease with PS1 mutations. *Amyloid* 20(2)**,** 107-112. doi: 10.3109/13506129.2013.790810.

Ishikawa, A., Piao, Y.S., Miyashita, A., Kuwano, R., Onodera, O., Ohtake, H., et al. (2005). A mutant PSEN1 causes dementia with Lewy bodies and variant Alzheimer's disease. *Ann Neurol* 57(3)**,** 429-434. doi: 10.1002/ana.20393.

Itzcovich, T., Chrem-Méndez, P., Vázquez, S., Barbieri-Kennedy, M., Niikado, M., Martinetto, H., et al. (2020). A novel mutation in PSEN1 (p.T119I) in an Argentine family with early- and late-onset Alzheimer's disease. *Neurobiol Aging* 85**,** 155.e159-155.e112. doi: 10.1016/j.neurobiolaging.2019.05.001.

Janssen, J.C., Beck, J.A., Campbell, T.A., Dickinson, A., Fox, N.C., Harvey, R.J., et al. (2003). Early onset familial Alzheimer's disease: Mutation frequency in 31 families. *Neurology* 60(2)**,** 235-239. doi: 10.1212/01.wnl.0000042088.22694.e3.

Jayadev, S., Leverenz, J.B., Steinbart, E., Stahl, J., Klunk, W., Yu, C.E., et al. (2010). Alzheimer's disease phenotypes and genotypes associated with mutations in presenilin 2. *Brain* 133(Pt 4)**,** 1143-1154. doi: 10.1093/brain/awq033.

Jia, J., Xu, E., Shao, Y., Jia, J., Sun, Y., and Li, D. (2005). One novel presenilin-1 gene mutation in a Chinese pedigree of familial Alzheimer's disease. *J Alzheimers Dis* 7(2)**,** 119-124; discussion 173-180. doi: 10.3233/jad-2005-7204.

Jiang, B., Zhou, J., Li, H.L., Chen, Y.G., Cheng, H.R., Ye, L.Q., et al. (2019). Mutation screening in Chinese patients with familial Alzheimer's disease by whole-exome sequencing. *Neurobiol Aging* 76**,** 215.e215-215.e221. doi: 10.1016/j.neurobiolaging.2018.11.024.

Jiang, H.Y., Li, G.D., Dai, S.X., Bi, R., Zhang, D.F., Li, Z.F., et al. (2015). Identification of PSEN1 mutations p.M233L and p.R352C in Han Chinese families with early-onset familial Alzheimer's disease. *Neurobiol Aging* 36(3)**,** 1602.e1603-1606. doi: 10.1016/j.neurobiolaging.2014.11.009.

Jiao, B., Tang, B., Liu, X., Xu, J., Wang, Y., Zhou, L., et al. (2014). Mutational analysis in early-onset familial Alzheimer's disease in Mainland China. *Neurobiol Aging* 35(8)**,** 1957.e1951-1956. doi: 10.1016/j.neurobiolaging.2014.02.014.

Jimenez-Escrig, A., Rabano, A., Guerrero, C., Simon, J., Barquero, M.S., Güell, I., et al. (2004). New V272A presenilin 1 mutation with very early onset subcortical dementia and parkinsonism. *Eur J Neurol* 11(10)**,** 663-669. doi: 10.1111/j.1468-1331.2004.00865.x.

Jiménez Caballero, P.E., Lladó, A., de Diego Boguna, C., Martin Correa, E., Serviá Candela, M., and Marsal Alonso, C. (2008). A novel presenilin 1 mutation (V261L) associated with presenile Alzheimer's disease and spastic paraparesis. *Eur J Neurol* 15(9)**,** 991-994. doi: 10.1111/j.1468-1331.2008.02230.x.

Jin, S.C., Pastor, P., Cooper, B., Cervantes, S., Benitez, B.A., Razquin, C., et al. (2012). Pooled-DNA sequencing identifies novel causative variants in PSEN1, GRN and MAPT in a clinical early-onset and familial Alzheimer's disease Ibero-American cohort. *Alzheimers Res Ther* 4(4)**,** 34. doi: 10.1186/alzrt137.

Jones, C.T., Morris, S., Yates, C.M., Moffoot, A., Sharpe, C., Brock, D.J., et al. (1992). Mutation in codon 713 of the beta amyloid precursor protein gene presenting with schizophrenia. *Nat Genet* 1(4)**,** 306-309. doi: 10.1038/ng0792-306.

Jonsson, T., Atwal, J.K., Steinberg, S., Snaedal, J., Jonsson, P.V., Bjornsson, S., et al. (2012). A mutation in APP protects against Alzheimer's disease and age-related cognitive decline. *Nature* 488(7409)**,** 96-99. doi: 10.1038/nature11283.

Jørgensen, P., Bus, C., Pallisgaard, N., Bryder, M., and Jørgensen, A.L. (1996). Familial Alzheimer's disease co-segregates with a Met146I1e substitution in presenilin-1. *Clin Genet* 50(5)**,** 281-286.

Kaden, D., Harmeier, A., Weise, C., Munter, L.M., Althoff, V., Rost, B.R., et al. (2012). Novel APP/Aβ mutation K16N produces highly toxic heteromeric Aβ oligomers. *EMBO Mol Med* 4(7)**,** 647-659. doi: 10.1002/emmm.201200239.

Kamimura, K., Tanahashi, H., Yamanaka, H., Takahashi, K., Asada, T., and Tabira, T. (1998). Familial Alzheimer's disease genes in Japanese. *J Neurol Sci* 160(1)**,** 76-81. doi: 10.1016/s0022-510x(98)00219-6.

Kamino, K., Orr, H.T., Payami, H., Wijsman, E.M., Alonso, M.E., Pulst, S.M., et al. (1992). Linkage and mutational analysis of familial Alzheimer disease kindreds for the APP gene region. *Am J Hum Genet* 51(5)**,** 998-1014.

Kamino, K., Sato, S., Sakaki, Y., Yoshiiwa, A., Nishiwaki, Y., Takeda, M., et al. (1996). Three different mutations of presenilin 1 gene in early-onset Alzheimer's disease families. *Neurosci Lett* 208(3)**,** 195-198. doi: 10.1016/0304-3940(96)12587-8.

Kasuga, K., Ohno, T., Ishihara, T., Miyashita, A., Kuwano, R., Onodera, O., et al. (2009). Depression and psychiatric symptoms preceding onset of dementia in a family with early-onset Alzheimer disease with a novel PSEN1 mutation. *J Neurol* 256(8)**,** 1351-1353. doi: 10.1007/s00415-009-5096-4.

Kerchner, G.A., and Holbrook, K. (2012). Novel presenilin-1 Y159F sequence variant associated with early-onset Alzheimer's disease. *Neurosci Lett* 531(2)**,** 142-144. doi: 10.1016/j.neulet.2012.10.037.

Kim, H.J., Kim, H.Y., Ki, C.S., and Kim, S.H. (2010). Presenilin 1 gene mutation (M139I) in a patient with an early-onset Alzheimer's disease: clinical characteristics and genetic identification. *Neurol Sci* 31(6)**,** 781-783. doi: 10.1007/s10072-010-0233-5.

Kim, J., Bagyinszky, E., Chang, Y.H., Choe, G., Choi, B.O., An, S.S., et al. (2012). A novel PSEN1 H163P mutation in a patient with early-onset Alzheimer's disease: clinical, neuroimaging, and neuropathological findings. *Neurosci Lett* 530(2)**,** 109-114. doi: 10.1016/j.neulet.2012.09.040.

Kim, Y.E., Cho, H., Kim, H.J., Na, D.L., Seo, S.W., and Ki, C.S. (2020). PSEN1 variants in Korean patients with clinically suspicious early-onset familial Alzheimer's disease. *Sci Rep* 10(1)**,** 3480. doi: 10.1038/s41598-020-59829-z.

Klünemann, H.H., Rogaeva, E., Neumann, M., Kretzschmar, H.A., Kandel, M., Toulina, A., et al. (2004). Novel PS1 mutation in a Bavarian kindred with familial Alzheimer disease. *Alzheimer Dis Assoc Disord* 18(4)**,** 256-258.

Knight, W.D., Kennedy, J., Mead, S., Rossor, M.N., Beck, J., Collinge, J., et al. (2007). A novel presenilin 1 deletion (p.L166del) associated with early onset familial Alzheimer's disease. *Eur J Neurol* 14(7)**,** 829-831. doi: 10.1111/j.1468-1331.2007.01857.x.

Koriath, C., Kenny, J., Adamson, G., Druyeh, R., Taylor, W., Beck, J., et al. (2020). Predictors for a dementia gene mutation based on gene-panel next-generation sequencing of a large dementia referral series. *Mol Psychiatry* 25(12)**,** 3399-3412. doi: 10.1038/s41380-018-0224-0.

Kowalska, A., Forsell, C., Florczak, J., Pruchnik-Wolińska, D., Modestowicz, R., Paprzycki, W., et al. (1999). A Polish pedigree with Alzheimer's disease determined by a novel mutation in exon 12 of the presenilin 1 gene: clinical and molecular characterization. *Folia Neuropathol* 37(1)**,** 57-61.

Kowalska, A., Wender, M., Florczak, J., Pruchnik-Wolinska, D., Modestowicz, R., Szczech, J., et al. (2003). Molecular genetics of Alzheimer's disease: presenilin 1 gene analysis in a cohort of patients from the Poznań region. *J Appl Genet* 44(2)**,** 231-234.

Kumar-Singh, S., De Jonghe, C., Cruts, M., Kleinert, R., Wang, R., Mercken, M., et al. (2000). Nonfibrillar diffuse amyloid deposition due to a gamma(42)-secretase site mutation points to an essential role for N-truncated A beta(42) in Alzheimer's disease. *Hum Mol Genet* 9(18)**,** 2589-2598. doi: 10.1093/hmg/9.18.2589.

Kutoku, Y., Ohsawa, Y., Kuwano, R., Ikeuchi, T., Inoue, H., Ataka, S., et al. (2015). A second pedigree with amyloid-less familial Alzheimer's disease harboring an identical mutation in the amyloid precursor protein gene (E693delta). *Intern Med* 54(2)**,** 205-208. doi: 10.2169/internalmedicine.54.3021.

Kwok, J.B., Halliday, G.M., Brooks, W.S., Dolios, G., Laudon, H., Murayama, O., et al. (2003). Presenilin-1 mutation L271V results in altered exon 8 splicing and Alzheimer's disease with non-cored plaques and no neuritic dystrophy. *J Biol Chem* 278(9)**,** 6748-6754. doi: 10.1074/jbc.M211827200.

Kwok, J.B., Li, Q.X., Hallupp, M., Whyte, S., Ames, D., Beyreuther, K., et al. (2000). Novel Leu723Pro amyloid precursor protein mutation increases amyloid beta42(43) peptide levels and induces apoptosis. *Ann Neurol* 47(2)**,** 249-253. doi: 10.1002/1531-8249(200002)47:2<249::aid-ana18>3.0.co;2-8.

Kwok, J.B., Taddei, K., Hallupp, M., Fisher, C., Brooks, W.S., Broe, G.A., et al. (1997). Two novel (M233T and R278T) presenilin-1 mutations in early-onset Alzheimer's disease pedigrees and preliminary evidence for association of presenilin-1 mutations with a novel phenotype. *Neuroreport* 8(6)**,** 1537-1542. doi: 10.1097/00001756-199704140-00043.

Lalli, M.A., Cox, H.C., Arcila, M.L., Cadavid, L., Moreno, S., Garcia, G., et al. (2014). Origin of the PSEN1 E280A mutation causing early-onset Alzheimer's disease. *Alzheimers Dement* 10(5 Suppl)**,** S277-S283.e210. doi: 10.1016/j.jalz.2013.09.005.

Lanoiselée, H.M., Nicolas, G., Wallon, D., Rovelet-Lecrux, A., Lacour, M., Rousseau, S., et al. (2017). APP, PSEN1, and PSEN2 mutations in early-onset Alzheimer disease: A genetic screening study of familial and sporadic cases. *PLoS Med* 14(3)**,** e1002270. doi: 10.1371/journal.pmed.1002270.

Lao, J.I., Beyer, K., Fernández-Novoa, L., and Cacabelos, R. (1998). A novel mutation in the predicted TM2 domain of the presenilin 2 gene in a Spanish patient with late-onset Alzheimer's disease. *Neurogenetics* 1(4)**,** 293-296. doi: 10.1007/s100480050044.

Le Guennec, K., Veugelen, S., Quenez, O., Szaruga, M., Rousseau, S., Nicolas, G., et al. (2017). Deletion of exons 9 and 10 of the Presenilin 1 gene in a patient with Early-onset Alzheimer Disease generates longer amyloid seeds. *Neurobiol Dis* 104**,** 97-103. doi: 10.1016/j.nbd.2017.04.020.

Lee, J.H., Kahn, A., Cheng, R., Reitz, C., Vardarajan, B., Lantigua, R., et al. (2014). Disease-related mutations among Caribbean Hispanics with familial dementia. *Mol Genet Genomic Med* 2(5)**,** 430-437. doi: 10.1002/mgg3.85.

Levy-Lahad, E., Wasco, W., Poorkaj, P., Romano, D.M., Oshima, J., Pettingell, W.H., et al. (1995). Candidate gene for the chromosome 1 familial Alzheimer's disease locus. *Science* 269(5226)**,** 973-977. doi: 10.1126/science.7638622.

Levy, E., Carman, M.D., Fernandez-Madrid, I.J., Power, M.D., Lieberburg, I., van Duinen, S.G., et al. (1990). Mutation of the Alzheimer's disease amyloid gene in hereditary cerebral hemorrhage, Dutch type. *Science* 248(4959)**,** 1124-1126. doi: 10.1126/science.2111584.

Lewis, P.A., Perez-Tur, J., Golde, T.E., and Hardy, J. (2000). The presenilin 1 C92S mutation increases abeta 42 production. *Biochem Biophys Res Commun* 277(1)**,** 261-263. doi: 10.1006/bbrc.2000.3646.

Li, D., Parks, S.B., Kushner, J.D., Nauman, D., Burgess, D., Ludwigsen, S., et al. (2006). Mutations of presenilin genes in dilated cardiomyopathy and heart failure. *Am J Hum Genet* 79(6)**,** 1030-1039. doi: 10.1086/509900.

Li, L., Kim, H.J., Roh, J.H., Kim, M., Koh, W., Kim, Y., et al. (2020). Pathological manifestation of the induced pluripotent stem cell-derived cortical neurons from an early-onset Alzheimer's disease patient carrying a presenilin-1 mutation (S170F). *Cell Prolif* 53(4)**,** e12798. doi: 10.1111/cpr.12798.

Li, Y.S., Yang, Z.H., Zhang, Y., Yang, J., Shang, D.D., Zhang, S.Y., et al. (2019). Two Novel Mutations and a de novo Mutation in PSEN1 in Early-onset Alzheimer's Disease. *Aging Dis* 10(4)**,** 908-914. doi: 10.14336/ad.2018.1109.

Lindquist, S.G., Hasholt, L., Bahl, J.M., Heegaard, N.H., Andersen, B.B., Nørremølle, A., et al. (2008a). A novel presenilin 2 mutation (V393M) in early-onset dementia with profound language impairment. *Eur J Neurol* 15(10)**,** 1135-1139. doi: 10.1111/j.1468-1331.2008.02256.x.

Lindquist, S.G., Nielsen, J.E., Stokholm, J., Schwartz, M., Batbayli, M., Ballegaard, M., et al. (2008b). Atypical early-onset Alzheimer's disease caused by the Iranian APP mutation. *J Neurol Sci* 268(1-2)**,** 124-130. doi: 10.1016/j.jns.2007.11.021.

Liu, C.Y., Ohki, Y., Tomita, T., Osawa, S., Reed, B.R., Jagust, W., et al. (2017). Two Novel Mutations in the First Transmembrane Domain of Presenilin1 Cause Young-Onset Alzheimer's Disease. *J Alzheimers Dis* 58(4)**,** 1035-1041. doi: 10.3233/jad-161203.

Liu, J., Wang, Q., Jing, D., Gao, R., Zhang, J., Cui, C., et al. (2019). Diagnostic Approach of Early-Onset Dementia with Negative Family History: Implications from Two Cases of Early-Onset Alzheimer's Disease with De Novo PSEN1 Mutation. *J Alzheimers Dis* 68(2)**,** 551-558. doi: 10.3233/jad-181108.

Lladó, A., Fortea, J., Ojea, T., Bosch, B., Sanz, P., Valls-Solé, J., et al. (2010). A novel PSEN1 mutation (K239N) associated with Alzheimer's disease with wide range age of onset and slow progression. *Eur J Neurol* 17(7)**,** 994-996. doi: 10.1111/j.1468-1331.2010.02949.x.

Lleó, A., Blesa, R., Gendre, J., Castellví, M., Pastor, P., Queralt, R., et al. (2001). A novel presenilin 2 gene mutation (D439A) in a patient with early-onset Alzheimer's disease. *Neurology* 57(10)**,** 1926-1928. doi: 10.1212/wnl.57.10.1926.

Lleó, A., Blesa, R., Queralt, R., Ezquerra, M., Molinuevo, J.L., Peña-Casanova, J., et al. (2002a). Frequency of mutations in the presenilin and amyloid precursor protein genes in early-onset Alzheimer disease in Spain. *Arch Neurol* 59(11)**,** 1759-1763. doi: 10.1001/archneur.59.11.1759.

Lleó, A., Castellví, M., Blesa, R., and Oliva, R. (2002b). Uncommon polymorphism in the presenilin genes in human familial Alzheimer's disease: not to be mistaken with a pathogenic mutation. *Neurosci Lett* 318(3)**,** 166-168. doi: 10.1016/s0304-3940(01)02499-5.

Lohmann, E., Guerreiro, R.J., Erginel-Unaltuna, N., Gurunlian, N., Bilgic, B., Gurvit, H., et al. (2012). Identification of PSEN1 and PSEN2 gene mutations and variants in Turkish dementia patients. *Neurobiol Aging* 33(8)**,** 1850.e1817-1827. doi: 10.1016/j.neurobiolaging.2012.02.020.

López-García, S., Jiménez-Bonilla, J., López Delgado, A., Orizaola Balaguer, P., Infante Ceberio, J., Banzo Marraco, I., et al. (2019). A Rare PSEN1 (Leu85Pro) Mutation Causing Alzheimer's Disease in a 29-Year-Old Woman Presenting as Corticobasal Syndrome. *J Alzheimers Dis* 70(3)**,** 655-658. doi: 10.3233/jad-190107.

Lou, F., Luo, X., Li, M., Ren, Y., and He, Z. (2017). Very early-onset sporadic Alzheimer's disease with a de novo mutation in the PSEN1 gene. *Neurobiol Aging* 53**,** 193.e191-193.e195. doi: 10.1016/j.neurobiolaging.2016.12.026.

Luedecke, D., Becktepe, J.S., Lehmbeck, J.T., Finckh, U., Yamamoto, R., Jahn, H., et al. (2014). A novel presenilin 1 mutation (Ala275Val) as cause of early-onset familial Alzheimer disease. *Neurosci Lett* 566**,** 115-119. doi: 10.1016/j.neulet.2014.02.034.

Maesako, M., Horlacher, J., Zoltowska, K.M., Kastanenka, K.V., Kara, E., Svirsky, S., et al. (2017). Pathogenic PS1 phosphorylation at Ser367. *Elife* 6. doi: 10.7554/eLife.19720.

Mangone, C.A., Castaño, E.M., Levy, E., Abiusi, G., Wisniewski, T., Marques, M.R., et al. (1995). Early onset Alzheimer's disease in a South American pedigree from Argentina. *Acta Neurol Scand* 91(1)**,** 6-13. doi: 10.1111/j.1600-0404.1995.tb05835.x.

Mann, D.M., Pickering-Brown, S.M., Takeuchi, A., and Iwatsubo, T. (2001). Amyloid angiopathy and variability in amyloid beta deposition is determined by mutation position in presenilin-1-linked Alzheimer's disease. *Am J Pathol* 158(6)**,** 2165-2175. doi: 10.1016/s0002-9440(10)64688-3.

Marcon, G., Di Fede, G., Giaccone, G., Rossi, G., Giovagnoli, A.R., Maccagnano, E., et al. (2009). A novel Italian presenilin 2 gene mutation with prevalent behavioral phenotype. *J Alzheimers Dis* 16(3)**,** 509-511. doi: 10.3233/jad-2009-0986.

Marín-Muñoz, J., Noguera-Perea, M.F., Gómez-Tortosa, E., López-Motos, D., Antequera-Torres, M., Martínez-Herrada, B., et al. (2016). Novel Mutation (Gly212Val) in the PS2 Gene Associated with Early-Onset Familial Alzheimer's Disease. *J Alzheimers Dis* 53(1)**,** 73-78. doi: 10.3233/jad-160050.

Marrosu, M.G., Floris, G., Costa, G., Schirru, L., Spinicci, G., Cherchi, M.V., et al. (2006). Dementia, pyramidal system involvement, and leukoencephalopathy with a presenilin 1 mutation. *Neurology* 66(1)**,** 108-111. doi: 10.1212/01.wnl.0000191360.08881.12.

Matsubara-Tsutsui, M., Yasuda, M., Yamagata, H., Nomura, T., Taguchi, K., Kohara, K., et al. (2002). Molecular evidence of presenilin 1 mutation in familial early onset dementia. *Am J Med Genet* 114(3)**,** 292-298. doi: 10.1002/ajmg.10250.

Matsushita, S., Arai, H., Okamura, N., Ohmori, T., Takasugi, K., Matsui, T., et al. (2002). Clinical and biomarker investigation of a patient with a novel presenilin-1 mutation (A431V) in the mild cognitive impairment stage of Alzheimer's disease. *Biol Psychiatry* 52(9)**,** 907-910. doi: 10.1016/s0006-3223(02)01386-0.

Mendez, M.F., and McMurtray, A. (2006). Frontotemporal dementia-like phenotypes associated with presenilin-1 mutations. *Am J Alzheimers Dis Other Demen* 21(4)**,** 281-286. doi: 10.1177/1533317506290448.

Mengel, D., Liu, L., Yamamoto, R., Zülow, S., Deuschl, C., Hermann, D.M., et al. (2020). A novel V272D presenilin mutation associated with logopenia, disorientation, and apraxia in an autosomal-dominant Alzheimer's disease family. *Neurobiol Aging* 85**,** 154.e155-154.e157. doi: 10.1016/j.neurobiolaging.2019.07.002.

Mehrabian S, Traykov L, Jordanova A, Rademakers R, Cruts M, Raycheva M, et al. (2006). Novel PSEN1 gene mutation in a large Bulgarian pedigree with Alzheimer's disease and atypical phenotype. European Journal of Neurology. 13.

Miki, T., Yokota, O., Haraguchi, T., Ikeuchi, T., Zhu, B., Takenoshita, S., et al. (2019). Young adult-onset, very slowly progressive cognitive decline with spastic paraparesis in Alzheimer's disease with cotton wool plaques due to a novel presenilin1 G417S mutation. *Acta Neuropathol Commun* 7(1)**,** 19. doi: 10.1186/s40478-019-0672-z.

Miklossy, J., Taddei, K., Suva, D., Verdile, G., Fonte, J., Fisher, C., et al. (2003). Two novel presenilin-1 mutations (Y256S and Q222H) are associated with early-onset Alzheimer's disease. *Neurobiol Aging* 24(5)**,** 655-662. doi: 10.1016/s0197-4580(02)00192-6.

Miravalle, L., Calero, M., Takao, M., Roher, A.E., Ghetti, B., and Vidal, R. (2005). Amino-terminally truncated Abeta peptide species are the main component of cotton wool plaques. *Biochemistry* 44(32)**,** 10810-10821. doi: 10.1021/bi0508237.

Moehlmann, T., Winkler, E., Xia, X., Edbauer, D., Murrell, J., Capell, A., et al. (2002). Presenilin-1 mutations of leucine 166 equally affect the generation of the Notch and APP intracellular domains independent of their effect on Abeta 42 production. *Proc Natl Acad Sci U S A* 99(12)**,** 8025-8030. doi: 10.1073/pnas.112686799.

Monacelli, F., Martella, L., Parodi, M.N., Odetti, P., Fanelli, F., and Tabaton, M. (2019). Frontal Variant of Alzheimer's Disease: A Report of a Novel PSEN1 Mutation. *J Alzheimers Dis* 70(1)**,** 11-15. doi: 10.3233/jad-190363.

Moretti, P., Lieberman, A.P., Wilde, E.A., Giordani, B.I., Kluin, K.J., Koeppe, R.A., et al. (2004). Novel insertional presenilin 1 mutation causing Alzheimer disease with spastic paraparesis. *Neurology* 62(10)**,** 1865-1868. doi: 10.1212/01.wnl.0000126447.91111.a1.

Mullan, M., Crawford, F., Axelman, K., Houlden, H., Lilius, L., Winblad, B., et al. (1992). A pathogenic mutation for probable Alzheimer's disease in the APP gene at the N-terminus of beta-amyloid. *Nat Genet* 1(5)**,** 345-347. doi: 10.1038/ng0892-345.

Müller, U., Winter, P., Bolender, C., and Nolte, D. (2014). Previously unrecognized missense mutation E126K of PSEN2 segregates with early onset Alzheimer's disease in a family. *J Alzheimers Dis* 42(1)**,** 109-113. doi: 10.3233/jad-140399.

Müller, U., Winter, P., and Graeber, M.B. (2013). A presenilin 1 mutation in the first case of Alzheimer's disease. *Lancet Neurol* 12(2)**,** 129-130. doi: 10.1016/s1474-4422(12)70307-1.

Murayama, O., Tomita, T., Nihonmatsu, N., Murayama, M., Sun, X., Honda, T., et al. (1999). Enhancement of amyloid beta 42 secretion by 28 different presenilin 1 mutations of familial Alzheimer's disease. *Neurosci Lett* 265(1)**,** 61-63. doi: 10.1016/s0304-3940(99)00187-1.

Murrell, J., Farlow, M., Ghetti, B., and Benson, M.D. (1991). A mutation in the amyloid precursor protein associated with hereditary Alzheimer's disease. *Science* 254(5028)**,** 97-99. doi: 10.1126/science.1925564.

Murrell, J.R., Hake, A.M., Quaid, K.A., Farlow, M.R., and Ghetti, B. (2000). Early-onset Alzheimer disease caused by a new mutation (V717L) in the amyloid precursor protein gene. *Arch Neurol* 57(6)**,** 885-887. doi: 10.1001/archneur.57.6.885.

Natelson Love, M., Clark, D.G., Cochran, J.N., Den Beste, K.A., Geldmacher, D.S., Benzinger, T.L., et al. (2017). Clinical, imaging, pathological, and biochemical characterization of a novel presenilin 1 mutation (N135Y) causing Alzheimer's disease. *Neurobiol Aging* 49**,** 216.e217-216.e213. doi: 10.1016/j.neurobiolaging.2016.09.020.

Nicolas, G., Wallon, D., Charbonnier, C., Quenez, O., Rousseau, S., Richard, A.C., et al. (2016a). Screening of dementia genes by whole-exome sequencing in early-onset Alzheimer disease: input and lessons. *Eur J Hum Genet* 24(5)**,** 710-716. doi: 10.1038/ejhg.2015.173.

Nicolas, G., Wallon, D., Goupil, C., Richard, A.C., Pottier, C., Dorval, V., et al. (2016b). Mutation in the 3'untranslated region of APP as a genetic determinant of cerebral amyloid angiopathy. *Eur J Hum Genet* 24(1)**,** 92-98. doi: 10.1038/ejhg.2015.61.

Nilsberth, C., Westlind-Danielsson, A., Eckman, C.B., Condron, M.M., Axelman, K., Forsell, C., et al. (2001). The 'Arctic' APP mutation (E693G) causes Alzheimer's disease by enhanced Abeta protofibril formation. *Nat Neurosci* 4(9)**,** 887-893. doi: 10.1038/nn0901-887.

Niu, F., Yu, S., Zhang, Z., Yi, X., Ye, L., Tang, W., et al. (2014). Novel mutation in the PSEN2 gene (N141Y) associated with early-onset autosomal dominant Alzheimer's disease in a Chinese Han family. *Neurobiol Aging* 35(10)**,** 2420.e2421-2425. doi: 10.1016/j.neurobiolaging.2014.04.011.

Norton, J.B., Cairns, N.J., Chakraverty, S., Wang, J., Levitch, D., Galvin, J.E., et al. (2009). Presenilin1 G217R mutation linked to Alzheimer disease with cotton wool plaques. *Neurology* 73(6)**,** 480-482. doi: 10.1212/WNL.0b013e3181b163ba.

Nygaard, H.B., Lippa, C.F., Mehdi, D., and Baehring, J.M. (2014). A Novel Presenilin 1 Mutation in Early-Onset Alzheimer's Disease With Prominent Frontal Features. *Am J Alzheimers Dis Other Demen* 29(5)**,** 433-435. doi: 10.1177/1533317513518653.

Obici, L., Demarchi, A., de Rosa, G., Bellotti, V., Marciano, S., Donadei, S., et al. (2005). A novel AbetaPP mutation exclusively associated with cerebral amyloid angiopathy. *Ann Neurol* 58(4)**,** 639-644. doi: 10.1002/ana.20571.

Palmer, M.S., Beck, J.A., Campbell, T.A., Humphries, C.B., Roques, P.K., Fox, N.C., et al. (1999). Pathogenic presenilin 1 mutations (P436S & I143F) in early-onset Alzheimer's disease in the UK. Mutations in brief no. 223. Online. *Hum Mutat* 13(3)**,** 256. doi: 10.1002/(sici)1098-1004(1999)13:3<256::aid-humu11>3.0.co;2-p.

Pantieri, R., Pardini, M., Cecconi, M., Dagna-Bricarelli, F., Vitali, A., Piccini, A., et al. (2005). A novel presenilin 1 L166H mutation in a pseudo-sporadic case of early-onset Alzheimer's disease. *Neurol Sci* 26(5)**,** 349-350. doi: 10.1007/s10072-005-0499-1.

Park, H.K., Na, D.L., Lee, J.H., Kim, J.W., and Ki, C.S. (2008). Identification of PSEN1 and APP gene mutations in Korean patients with early-onset Alzheimer's disease. *J Korean Med Sci* 23(2)**,** 213-217. doi: 10.3346/jkms.2008.23.2.213.

Park, J., An, S.S.A., Giau, V.V., Shim, K., Youn, Y.C., Bagyinszky, E., et al. (2017). Identification of a novel PSEN1 mutation (Leu232Pro) in a Korean patient with early-onset Alzheimer's disease and a family history of dementia. *Neurobiol Aging* 56**,** 212.e211-212.e217. doi: 10.1016/j.neurobiolaging.2017.04.012.

Park, J.E., Kim, H.J., Kim, Y.E., Jang, H., Cho, S.H., Kim, S.J., et al. (2020). Analysis of dementia-related gene variants in APOE ε4 noncarrying Korean patients with early-onset Alzheimer's disease. *Neurobiol Aging* 85**,** 155.e155-155.e158. doi: 10.1016/j.neurobiolaging.2019.05.009.

Pasalar, P., Najmabadi, H., Noorian, A.R., Moghimi, B., Jannati, A., Soltanzadeh, A., et al. (2002). An Iranian family with Alzheimer's disease caused by a novel APP mutation (Thr714Ala). *Neurology* 58(10)**,** 1574-1575. doi: 10.1212/wnl.58.10.1574.

Peacock, M.L., Murman, D.L., Sima, A.A., Warren, J.T., Jr., Roses, A.D., and Fink, J.K. (1994). Novel amyloid precursor protein gene mutation (codon 665Asp) in a patient with late-onset Alzheimer's disease. *Ann Neurol* 35(4)**,** 432-438. doi: 10.1002/ana.410350410.

Peacock, M.L., Warren, J.T., Jr., Roses, A.D., and Fink, J.K. (1993). Novel polymorphism in the A4 region of the amyloid precursor protein gene in a patient without Alzheimer's disease. *Neurology* 43(6)**,** 1254-1256. doi: 10.1212/wnl.43.6.1254.

Perez-Tur, J., Croxton, R., Wright, K., Phillips, H., Zehr, C., Crook, R., et al. (1996). A further presenilin 1 mutation in the exon 8 cluster in familial Alzheimer's disease. *Neurodegeneration* 5(3)**,** 207-212. doi: 10.1006/neur.1996.0028.

Perrone, F., Bjerke, M., Hens, E., Sieben, A., Timmers, M., De Roeck, A., et al. (2020). Amyloid-β(1-43) cerebrospinal fluid levels and the interpretation of APP, PSEN1 and PSEN2 mutations. *Alzheimers Res Ther* 12(1)**,** 108. doi: 10.1186/s13195-020-00676-5.

Perrone, F., Cacace, R., Van Mossevelde, S., Van den Bossche, T., De Deyn, P.P., Cras, P., et al. (2018). Genetic screening in early-onset dementia patients with unclear phenotype: relevance for clinical diagnosis. *Neurobiol Aging* 69**,** 292.e297-292.e214. doi: 10.1016/j.neurobiolaging.2018.04.015.

Piccoli, E., Rossi, G., Rossi, T., Pelliccioni, G., D'Amato, I., Tagliavini, F., et al. (2016). Novel PSEN1 mutations (H214N and R220P) associated with familial Alzheimer's disease identified by targeted exome sequencing. *Neurobiol Aging* 40**,** 192.e197-192.e111. doi: 10.1016/j.neurobiolaging.2016.01.134.

Piscopo, P., Marcon, G., Piras, M.R., Crestini, A., Campeggi, L.M., Deiana, E., et al. (2008). A novel PSEN2 mutation associated with a peculiar phenotype. *Neurology* 70(17)**,** 1549-1554. doi: 10.1212/01.wnl.0000310643.53587.87.

Piscopo, P., Talarico, G., Crestini, A., Gasparini, M., Malvezzi-Campeggi, L., Piacentini, E., et al. (2010). A novel mutation in the predicted TMIII domain of the PSEN2 gene in an Italian pedigree with atypical Alzheimer's disease. *J Alzheimers Dis* 20(1)**,** 43-47. doi: 10.3233/jad-2010-1369.

Poorkaj, P., Sharma, V., Anderson, L., Nemens, E., Alonso, M.E., Orr, H., et al. (1998). Missense mutations in the chromosome 14 familial Alzheimer's disease presenilin 1 gene. *Hum Mutat* 11(3)**,** 216-221. doi: 10.1002/(sici)1098-1004(1998)11:3<216::Aid-humu6>3.0.Co;2-f.

Portet, F., Dauvilliers, Y., Campion, D., Raux, G., Hauw, J.J., Lyon-Caen, O., et al. (2003). Very early onset AD with a de novo mutation in the presenilin 1 gene (Met 233 Leu). *Neurology* 61(8)**,** 1136-1137. doi: 10.1212/01.wnl.0000086811.39675.79.

Puschmann, A., Ross, O.A., Vilariño-Güell, C., Lincoln, S.J., Kachergus, J.M., Cobb, S.A., et al. (2009). A Swedish family with de novo alpha-synuclein A53T mutation: evidence for early cortical dysfunction. *Parkinsonism Relat Disord* 15(9)**,** 627-632. doi: 10.1016/j.parkreldis.2009.06.007.

Qiu, Q., Jia, L., Wang, Q., Zhao, L., Jin, H., Li, T., et al. (2020). Identification of a novel PSEN1 Gly111Val missense mutation in a Chinese pedigree with early-onset Alzheimer's disease. *Neurobiol Aging* 85**,** 155.e151-155.e154. doi: 10.1016/j.neurobiolaging.2019.05.018.

Qiu, Q., Shen, L., Jia, L., Wang, Q., Li, F., Li, Y., et al. (2019). A Novel PSEN1 M139L Mutation Found in a Chinese Pedigree with Early-Onset Alzheimer's Disease Increases Aβ42/Aβ40 ratio. *J Alzheimers Dis* 69(1)**,** 199-212. doi: 10.3233/jad-181291.

Queralt, R., Ezquerra, M., Lleó, A., Castellví, M., Gelpí, J., Ferrer, I., et al. (2002). A novel mutation (V89L) in the presenilin 1 gene in a family with early onset Alzheimer's disease and marked behavioural disturbances. *J Neurol Neurosurg Psychiatry* 72(2)**,** 266-269. doi: 10.1136/jnnp.72.2.266.

Raman, A., Lin, X., Suri, M., Hewitt, M., Constantinescu, C.S., and Phillips, M.F. (2007). A presenilin 1 mutation (Arg278Ser) associated with early onset Alzheimer's disease and spastic paraparesis. *J Neurol Sci* 260(1-2)**,** 78-82. doi: 10.1016/j.jns.2007.04.013.

Ramirez Aguilar, L., Acosta-Uribe, J., Giraldo, M.M., Moreno, S., Baena, A., Alzate, D., et al. (2019). Genetic origin of a large family with a novel PSEN1 mutation (Ile416Thr). *Alzheimers Dement* 15(5)**,** 709-719. doi: 10.1016/j.jalz.2018.12.010.

Ramos-Campoy, O., Antonell, A., Falgàs, N., Balasa, M., Borrego-Écija, S., Rodríguez-Santiago, B., et al. (2020). Screening of dementia genes by whole-exome sequencing in Spanish patients with early-onset dementia: likely pathogenic, uncertain significance and risk variants. *Neurobiol Aging* 93**,** e1-e9. doi: 10.1016/j.neurobiolaging.2020.02.008.

Raux, G., Gantier, R., Martin, C., Pothin, Y., Brice, A., Frebourg, T., et al. (2000a). A novel presenilin 1 missense mutation (L153V) segregating with early-onset autosomal dominant Alzheimer's disease. *Hum Mutat* 16(1)**,** 95. doi: 10.1002/1098-1004(200007)16:1<95::Aid-humu28>3.0.Co;2-h.

Raux, G., Gantier, R., Thomas-Anterion, C., Boulliat, J., Verpillat, P., Hannequin, D., et al. (2000b). Dementia with prominent frontotemporal features associated with L113P presenilin 1 mutation. *Neurology* 55(10)**,** 1577-1578. doi: 10.1212/wnl.55.10.1577.

Raux, G., Guyant-Maréchal, L., Martin, C., Bou, J., Penet, C., Brice, A., et al. (2005). Molecular diagnosis of autosomal dominant early onset Alzheimer's disease: an update. *J Med Genet* 42(10)**,** 793-795. doi: 10.1136/jmg.2005.033456.

Reznik-Wolf, H., Treves, T.A., Davidson, M., Aharon-Peretz, J., St George Hyslop, P.H., Chapman, J., et al. (1996). A novel mutation of presenilin 1 in familial Alzheimer's disease in Israel detected by denaturing gradient gel electrophoresis. *Hum Genet* 98(6)**,** 700-702. doi: 10.1007/s004390050288.

Ringman, J.M., Casado, M., Van Berlo, V., Pa, J., Joseph-Mathurin, N., Fagan, A.M., et al. (2017). A novel PSEN1 (S230N) mutation causing early-onset Alzheimer's Disease associated with prosopagnosia, hoarding, and Parkinsonism. *Neurosci Lett* 657**,** 11-15. doi: 10.1016/j.neulet.2017.07.046.

Ringman, J.M., Gylys, K.H., Medina, L.D., Fox, M., Kepe, V., Flores, D.L., et al. (2011). Biochemical, neuropathological, and neuroimaging characteristics of early-onset Alzheimer's disease due to a novel PSEN1 mutation. *Neurosci Lett* 487(3)**,** 287-292. doi: 10.1016/j.neulet.2010.10.039.

Ringman, J.M., Monsell, S., Ng, D.W., Zhou, Y., Nguyen, A., Coppola, G., et al. (2016). Neuropathology of Autosomal Dominant Alzheimer Disease in the National Alzheimer Coordinating Center Database. *J Neuropathol Exp Neurol* 75(3)**,** 284-290. doi: 10.1093/jnen/nlv028.

Robles, A., Sobrido, M.J., García-Murias, M., Prieto, J.M., Lema, M., Santos, D., et al. (2009). Clinical picture of a patient with a novel PSEN1 mutation (L424V). *Am J Alzheimers Dis Other Demen* 24(1)**,** 40-45. doi: 10.1177/1533317508324272.

Roeber, S., Müller-Sarnowski, F., Kress, J., Edbauer, D., Kuhlmann, T., Tüttelmann, F., et al. (2015). Three novel presenilin 1 mutations marking the wide spectrum of age at onset and clinical patterns in familial Alzheimer's disease. *J Neural Transm (Vienna)* 122(12)**,** 1715-1719. doi: 10.1007/s00702-015-1450-0.

Rogaev, E.I., Sherrington, R., Rogaeva, E.A., Levesque, G., Ikeda, M., Liang, Y., et al. (1995). Familial Alzheimer's disease in kindreds with missense mutations in a gene on chromosome 1 related to the Alzheimer's disease type 3 gene. *Nature* 376(6543)**,** 775-778. doi: 10.1038/376775a0.

Rogaeva, E.A., Fafel, K.C., Song, Y.Q., Medeiros, H., Sato, C., Liang, Y., et al. (2001). Screening for PS1 mutations in a referral-based series of AD cases: 21 novel mutations. *Neurology* 57(4)**,** 621-625. doi: 10.1212/wnl.57.4.621.

Romero, I., Jørgensen, P., Bolwig, G., Fraser, P.E., Rogaeva, E., Mann, D., et al. (1999). A presenilin-1 Thr116Asn substitution in a family with early-onset Alzheimer's disease. *Neuroreport* 10(11)**,** 2255-2260. doi: 10.1097/00001756-199908020-00006.

Rossor, M.N., Fox, N.C., Beck, J., Campbell, T.C., and Collinge, J. (1996). Incomplete penetrance of familial Alzheimer's disease in a pedigree with a novel presenilin-1 gene mutation. *Lancet* 347(9014)**,** 1560. doi: 10.1016/s0140-6736(96)90715-1.

Rovelet-Lecrux, A., Charbonnier, C., Wallon, D., Nicolas, G., Seaman, M.N., Pottier, C., et al. (2015). De novo deleterious genetic variations target a biological network centered on Aβ peptide in early-onset Alzheimer disease. *Mol Psychiatry* 20(9)**,** 1046-1056. doi: 10.1038/mp.2015.100.

Rudzinski, L.A., Fletcher, R.M., Dickson, D.W., Crook, R., Hutton, M.L., Adamson, J., et al. (2008). Early onset familial Alzheimer Disease with spastic paraparesis, dysarthria, and seizures and N135S mutation in PSEN1. *Alzheimer Dis Assoc Disord* 22(3)**,** 299-307. doi: 10.1097/WAD.0b013e3181732399.

Ryan, N.S., Nicholas, J.M., Weston, P.S.J., Liang, Y., Lashley, T., Guerreiro, R., et al. (2016). Clinical phenotype and genetic associations in autosomal dominant familial Alzheimer's disease: a case series. *Lancet Neurol* 15(13)**,** 1326-1335. doi: 10.1016/s1474-4422(16)30193-4.

Ryman, D.C., Acosta-Baena, N., Aisen, P.S., Bird, T., Danek, A., Fox, N.C., et al. (2014). Symptom onset in autosomal dominant Alzheimer disease: a systematic review and meta-analysis. *Neurology* 83(3)**,** 253-260. doi: 10.1212/wnl.0000000000000596.

Sala Frigerio, C., Lau, P., Troakes, C., Deramecourt, V., Gele, P., Van Loo, P., et al. (2015). On the identification of low allele frequency mosaic mutations in the brains of Alzheimer's disease patients. *Alzheimers Dement* 11(11)**,** 1265-1276. doi: 10.1016/j.jalz.2015.02.007.

Sánchez-Valle, R., Lladó, A., Ezquerra, M., Rey, M.J., Rami, L., and Molinuevo, J.L. (2007). A novel mutation in the PSEN1 gene (L286P) associated with familial early-onset dementia of Alzheimer type and lobar haematomas. *Eur J Neurol* 14(12)**,** 1409-1412. doi: 10.1111/j.1468-1331.2007.01988.x.

Sandbrink, R., Zhang, D., Schaeffer, S., Masters, C.L., Bauer, J., Förstl, H., et al. (1996). Missense mutations of the PS-1/S182 gene in German early-onset Alzheimer's disease patients. *Ann Neurol* 40(2)**,** 265-266. doi: 10.1002/ana.410400225.

Sarroca, S., Molina-Martínez, P., Aresté, C., Etzrodt, M., García de Frutos, P., Gasa, R., et al. (2016). Preservation of cell-survival mechanisms by the presenilin-1 K239N mutation may cause its milder clinical phenotype. *Neurobiol Aging* 46**,** 169-179. doi: 10.1016/j.neurobiolaging.2016.07.002.

Sassi, C., Guerreiro, R., Gibbs, R., Ding, J., Lupton, M.K., Troakes, C., et al. (2014a). Investigating the role of rare coding variability in Mendelian dementia genes (APP, PSEN1, PSEN2, GRN, MAPT, and PRNP) in late-onset Alzheimer's disease. *Neurobiol Aging* 35(12)**,** 2881.e2881-2881.e2886. doi: 10.1016/j.neurobiolaging.2014.06.002.

Sassi, C., Guerreiro, R., Gibbs, R., Ding, J., Lupton, M.K., Troakes, C., et al. (2014b). Exome sequencing identifies 2 novel presenilin 1 mutations (p.L166V and p.S230R) in British early-onset Alzheimer's disease. *Neurobiol Aging* 35(10)**,** 2422.e2413-2426. doi: 10.1016/j.neurobiolaging.2014.04.026.

Sato, S., Kamino, K., Miki, T., Doi, A., Ii, K., St George-Hyslop, P.H., et al. (1998). Splicing mutation of presenilin-1 gene for early-onset familial Alzheimer's disease. *Hum Mutat* Suppl 1**,** S91-94. doi: 10.1002/humu.1380110131.

Scacchi, R., Gambina, G., Moretto, G., and Corbo, R.M. (2007). A mutation screening by DHPLC of PSEN1 and APP genes reveals no significant variation associated with the sporadic late-onset form of Alzheimer's disease. *Neurosci Lett* 418(3)**,** 282-285. doi: 10.1016/j.neulet.2007.03.035.

Scahill, R.I., Ridgway, G.R., Bartlett, J.W., Barnes, J., Ryan, N.S., Mead, S., et al. (2013). Genetic influences on atrophy patterns in familial Alzheimer's disease: a comparison of APP and PSEN1 mutations. *J Alzheimers Dis* 35(1)**,** 199-212. doi: 10.3233/jad-121255.

Schulte, E.C., Fukumori, A., Mollenhauer, B., Hor, H., Arzberger, T., Perneczky, R., et al. (2015). Rare variants in β-Amyloid precursor protein (APP) and Parkinson's disease. *Eur J Hum Genet* 23(10)**,** 1328-1333. doi: 10.1038/ejhg.2014.300.

Seo, J., Byun, M.S., Yi, D., Lee, J.H., Jeon, S.Y., Shin, S.A., et al. (2020). Genetic associations of in vivo pathology influence Alzheimer's disease susceptibility. *Alzheimers Res Ther* 12(1)**,** 156. doi: 10.1186/s13195-020-00722-2.

Shea, Y.F., Chan, A.O., Chu, L.W., Lee, S.C., Law, C.Y., See, C.H., et al. (2017). Novel presenilin 1 mutation (p.F386I) in a Chinese family with early-onset Alzheimer's disease. *Neurobiol Aging* 50**,** 168.e169-168.e111. doi: 10.1016/j.neurobiolaging.2016.10.015.

Shen, L., Qin, W., Wu, L., Zhou, A., Tang, Y., Wang, Q., et al. (2019). Two novel presenilin-1 mutations (I249L and P433S) in early onset Chinese Alzheimer's pedigrees and their functional characterization. *Biochem Biophys Res Commun* 516(1)**,** 264-269. doi: 10.1016/j.bbrc.2019.05.185.

Sherrington, R., Rogaev, E.I., Liang, Y., Rogaeva, E.A., Levesque, G., Ikeda, M., et al. (1995). Cloning of a gene bearing missense mutations in early-onset familial Alzheimer's disease. *Nature* 375(6534)**,** 754-760. doi: 10.1038/375754a0.

Shi, Z., Wang, Y., Liu, S., Liu, M., Liu, S., Zhou, Y., et al. (2015). Clinical and neuroimaging characterization of Chinese dementia patients with PSEN1 and PSEN2 mutations. *Dement Geriatr Cogn Disord* 39(1-2)**,** 32-40. doi: 10.1159/000366272.

Shimojo, M., Sahara, N., Mizoroki, T., Funamoto, S., Morishima-Kawashima, M., Kudo, T., et al. (2008). Enzymatic characteristics of I213T mutant presenilin-1/gamma-secretase in cell models and knock-in mouse brains: familial Alzheimer disease-linked mutation impairs gamma-site cleavage of amyloid precursor protein C-terminal fragment beta. *J Biol Chem* 283(24)**,** 16488-16496. doi: 10.1074/jbc.M801279200.

Shrimpton, A.E., Schelper, R.L., Linke, R.P., Hardy, J., Crook, R., Dickson, D.W., et al. (2007). A presenilin 1 mutation (L420R) in a family with early onset Alzheimer disease, seizures and cotton wool plaques, but not spastic paraparesis. *Neuropathology* 27(3)**,** 228-232. doi: 10.1111/j.1440-1789.2007.00766.x.

Sieczkowski, E., Milenkovic, I., Venkataramani, V., Giera, R., Ströbel, T., Höftberger, R., et al. (2015). I716F AβPP mutation associates with the deposition of oligomeric pyroglutamate amyloid-β and α-synucleinopathy with Lewy bodies. *J Alzheimers Dis* 44(1)**,** 103-114. doi: 10.3233/jad-141524.

Sleegers, K., Roks, G., Theuns, J., Aulchenko, Y.S., Rademakers, R., Cruts, M., et al. (2004). Familial clustering and genetic risk for dementia in a genetically isolated Dutch population. *Brain* 127(Pt 7)**,** 1641-1649. doi: 10.1093/brain/awh179.

Smith, M.J., Gardner, R.J., Knight, M.A., Forrest, S.M., Beyreuther, K., Storey, E., et al. (1999). Early-onset Alzheimer's disease caused by a novel mutation at codon 219 of the presenilin-1 gene. *Neuroreport* 10(3)**,** 503-507. doi: 10.1097/00001756-199902250-00011.

Snider, B.J., Norton, J., Coats, M.A., Chakraverty, S., Hou, C.E., Jervis, R., et al. (2005). Novel presenilin 1 mutation (S170F) causing Alzheimer disease with Lewy bodies in the third decade of life. *Arch Neurol* 62(12)**,** 1821-1830. doi: 10.1001/archneur.62.12.1821.

Sodeyama, N., Iwata, T., Ishikawa, K., Mizusawa, H., Yamada, M., Itoh, Y., et al. (2001). Very early onset Alzheimer's disease with spastic paraparesis associated with a novel presenilin 1 mutation (Phe237Ile). *J Neurol Neurosurg Psychiatry* 71(4)**,** 556-557. doi: 10.1136/jnnp.71.4.556.

Steiner, H., Revesz, T., Neumann, M., Romig, H., Grim, M.G., Pesold, B., et al. (2001). A pathogenic presenilin-1 deletion causes abberrant Abeta 42 production in the absence of congophilic amyloid plaques. *J Biol Chem* 276(10)**,** 7233-7239. doi: 10.1074/jbc.M007183200.

Sugiyama, N., Suzuki, K., Matsumura, T., Kawanishi, C., Onishi, H., Yamada, Y., et al. (1999). A novel missense mutation (G209R) in exon 8 of the presenilin 1 gene in a Japanese family with presenile familial Alzheimer's disease. Mutation in brief no. 254. Online. *Hum Mutat* 14(1)**,** 90. doi: 10.1002/(sici)1098-1004(1999)14:1<90::Aid-humu19>3.0.Co;2-s.

Sun, L., Zhou, R., Yang, G., and Shi, Y. (2017). Analysis of 138 pathogenic mutations in presenilin-1 on the in vitro production of Aβ42 and Aβ40 peptides by γ-secretase. *Proc Natl Acad Sci U S A* 114(4)**,** E476-e485. doi: 10.1073/pnas.1618657114.

Szaruga, M., Veugelen, S., Benurwar, M., Lismont, S., Sepulveda-Falla, D., Lleo, A., et al. (2015). Qualitative changes in human γ-secretase underlie familial Alzheimer's disease. *J Exp Med* 212(12)**,** 2003-2013. doi: 10.1084/jem.20150892.

Tabira, T., Chui, D.H., Nakayama, H., Kuroda, S., and Shibuya, M. (2002). Alzheimer's disease with spastic paresis and cotton wool type plaques. *J Neurosci Res* 70(3)**,** 367-372. doi: 10.1002/jnr.10392.

Taddei, K., Kwok, J.B., Kril, J.J., Halliday, G.M., Creasey, H., Hallupp, M., et al. (1998). Two novel presenilin-1 mutations (Ser169Leu and Pro436Gln) associated with very early onset Alzheimer's disease. *Neuroreport* 9(14)**,** 3335-3339. doi: 10.1097/00001756-199810050-00034.

Takao, M., Ghetti, B., Hayakawa, I., Ikeda, E., Fukuuchi, Y., Miravalle, L., et al. (2002). A novel mutation (G217D) in the Presenilin 1 gene ( PSEN1) in a Japanese family: presenile dementia and parkinsonism are associated with cotton wool plaques in the cortex and striatum. *Acta Neuropathol* 104(2)**,** 155-170. doi: 10.1007/s00401-002-0536-6.

Tang, T.C., Hu, Y., Kienlen-Campard, P., El Haylani, L., Decock, M., Van Hees, J., et al. (2014). Conformational changes induced by the A21G Flemish mutation in the amyloid precursor protein lead to increased Aβ production. *Structure* 22(3)**,** 387-396. doi: 10.1016/j.str.2013.12.012.

Tedde, A., Bartoli, A., Piaceri, I., Ferrara, S., Bagnoli, S., Serio, A., et al. (2016). Novel presenilin 1 mutation (Ile408Thr) in an Italian family with late-onset Alzheimer's disease. *Neurosci Lett* 610**,** 150-153. doi: 10.1016/j.neulet.2015.11.004.

Tedde, A., Forleo, P., Nacmias, B., Piccini, C., Bracco, L., Piacentini, S., et al. (2000). A presenilin-1 mutation (Leu392Pro) in a familial AD kindred with psychiatric symptoms at onset. *Neurology* 55(10)**,** 1590-1591. doi: 10.1212/wnl.55.10.1590.

Tedde, A., Nacmias, B., Ciantelli, M., Forleo, P., Cellini, E., Bagnoli, S., et al. (2003). Identification of new presenilin gene mutations in early-onset familial Alzheimer disease. *Arch Neurol* 60(11)**,** 1541-1544. doi: 10.1001/archneur.60.11.1541.

Terreni L, Fogliarino S, Franceschi M, Forloni G. (2002). Novel pathogenic mutation in an Italian patient with familial Alzheimer's disease detected in APP gene. Neurobiol Aging. 23:S319.

Terreni L, Valeria C, Calella AM, Gavazzi A, Alberoni M, Grimadi LM, et al. (2000). A novel missense mutation (L219F) in exon 8 of the presenilin 1 gene in an italian family with presenile familial Alzheimer's disease. Neurobiol Aging. 21:176-7.

Testi, S., Peluso, S., Fabrizi, G.M., Antenora, A., Russo, C.V., Pappatà, S., et al. (2014). A novel PSEN1 mutation in a patient with sporadic early-onset Alzheimer's disease and prominent cerebellar ataxia. *J Alzheimers Dis* 41(3)**,** 709-714. doi: 10.3233/jad-140081.

Theuns, J., Marjaux, E., Vandenbulcke, M., Van Laere, K., Kumar-Singh, S., Bormans, G., et al. (2006). Alzheimer dementia caused by a novel mutation located in the APP C-terminal intracytosolic fragment. *Hum Mutat* 27(9)**,** 888-896. doi: 10.1002/humu.20402.

Thordardottir, S., Kinhult Ståhlbom, A., Almkvist, O., Thonberg, H., Eriksdotter, M., Zetterberg, H., et al. (2017). The effects of different familial Alzheimer's disease mutations on APP processing in vivo. *Alzheimers Res Ther* 9(1)**,** 9. doi: 10.1186/s13195-017-0234-1.

Thordardottir, S., Rodriguez-Vieitez, E., Almkvist, O., Ferreira, D., Saint-Aubert, L., Kinhult-Ståhlbom, A., et al. (2018). Reduced penetrance of the PSEN1 H163Y autosomal dominant Alzheimer mutation: a 22-year follow-up study. *Alzheimers Res Ther* 10(1)**,** 45. doi: 10.1186/s13195-018-0374-y.

Tiedt, H.O., Lueschow, A., Winter, P., and Müller, U. (2013). Previously not recognized deletion in presenilin-1 (p.Leu174del.) in a patient with early-onset familial Alzheimer's disease. *Neurosci Lett* 544**,** 115-118. doi: 10.1016/j.neulet.2013.03.056.

Ting, S.K., Benzinger, T., Kepe, V., Fagan, A., Coppola, G., Porter, V., et al. (2014). A novel PSEN1 mutation (I238M) associated with early-onset Alzheimer's disease in an African-American woman. *J Alzheimers Dis* 40(2)**,** 271-275. doi: 10.3233/jad-131844.

Tomiyama, T., Nagata, T., Shimada, H., Teraoka, R., Fukushima, A., Kanemitsu, H., et al. (2008). A new amyloid beta variant favoring oligomerization in Alzheimer's-type dementia. *Ann Neurol* 63(3)**,** 377-387. doi: 10.1002/ana.21321.

Tysoe, C., Whittaker, J., Xuereb, J., Cairns, N.J., Cruts, M., Van Broeckhoven, C., et al. (1998). A presenilin-1 truncating mutation is present in two cases with autopsy-confirmed early-onset Alzheimer disease. *Am J Hum Genet* 62(1)**,** 70-76. doi: 10.1086/301672.

Uttner, I., Kirchheiner, J., Tumani, H., Mottaghy, F.M., Lebedeva, E., Ozer, E., et al. (2010). A novel presenilin1 mutation (Q223R) associated with early onset Alzheimer's disease, dysarthria and spastic paraparesis and decreased Abeta levels in CSF. *Eur J Neurol* 17(4)**,** 631-633. doi: 10.1111/j.1468-1331.2009.02810.x.

Van Broeckhoven, C., Haan, J., Bakker, E., Hardy, J.A., Van Hul, W., Wehnert, A., et al. (1990). Amyloid beta protein precursor gene and hereditary cerebral hemorrhage with amyloidosis (Dutch). *Science* 248(4959)**,** 1120-1122. doi: 10.1126/science.1971458.

Van Giau, V., Pyun, J.M., Suh, J., Bagyinszky, E., An, S.S.A., and Kim, S.Y. (2019). A pathogenic PSEN1 Trp165Cys mutation associated with early-onset Alzheimer's disease. *BMC Neurol* 19(1)**,** 188. doi: 10.1186/s12883-019-1419-y.

Van Giau, V., Senanarong, V., Bagyinszky, E., Limwongse, C., An, S.S.A., and Kim, S. (2018). Identification of a novel mutation in APP gene in a Thai subject with early-onset Alzheimer's disease. *Neuropsychiatr Dis Treat* 14**,** 3015-3023. doi: 10.2147/ndt.S180174.

Vöglein, J., Willem, M., Trambauer, J., Schönecker, S., Dieterich, M., Biskup, S., et al. (2019). Identification of a rare presenilin 1 single amino acid deletion mutation (F175del) with unusual amyloid-β processing effects. *Neurobiol Aging* 84**,** 241.e245-241.e211. doi: 10.1016/j.neurobiolaging.2019.08.034.

Wakutani, Y., Watanabe, K., Adachi, Y., Wada-Isoe, K., Urakami, K., Ninomiya, H., et al. (2004). Novel amyloid precursor protein gene missense mutation (D678N) in probable familial Alzheimer's disease. *J Neurol Neurosurg Psychiatry* 75(7)**,** 1039-1042. doi: 10.1136/jnnp.2003.010611.

Walker, E.S., Martinez, M., Brunkan, A.L., and Goate, A. (2005). Presenilin 2 familial Alzheimer's disease mutations result in partial loss of function and dramatic changes in Abeta 42/40 ratios. *J Neurochem* 92(2)**,** 294-301. doi: 10.1111/j.1471-4159.2004.02858.x.

Wallon, D., Rousseau, S., Rovelet-Lecrux, A., Quillard-Muraine, M., Guyant-Maréchal, L., Martinaud, O., et al. (2012). The French series of autosomal dominant early onset Alzheimer's disease cases: mutation spectrum and cerebrospinal fluid biomarkers. *J Alzheimers Dis* 30(4)**,** 847-856. doi: 10.3233/jad-2012-120172.

Wang, B., Yang, W., Wen, W., Sun, J., Su, B., Liu, B., et al. (2010). Gamma-secretase gene mutations in familial acne inversa. *Science* 330(6007)**,** 1065. doi: 10.1126/science.1196284.

Wang, G., Zhang, D.F., Jiang, H.Y., Fan, Y., Ma, L., Shen, Z., et al. (2019). Mutation and association analyses of dementia-causal genes in Han Chinese patients with early-onset and familial Alzheimer's disease. *J Psychiatr Res* 113**,** 141-147. doi: 10.1016/j.jpsychires.2019.03.026.

Wang, J.C., Alinaghi, S., Tafakhori, A., Sikora, E., Azcona, L.J., Karkheiran, S., et al. (2018). Genetic screening in two Iranian families with early-onset Alzheimer's disease identified a novel PSEN1 mutation. *Neurobiol Aging* 62**,** 244.e215-244.e217. doi: 10.1016/j.neurobiolaging.2017.10.011.

Wang, Q., Jia, J., Qin, W., Wu, L., Li, D., Wang, Q., et al. (2015). A Novel AβPP M722K Mutation Affects Amyloid-β Secretion and Tau Phosphorylation and May Cause Early-Onset Familial Alzheimer's Disease in Chinese Individuals. *J Alzheimers Dis* 47(1)**,** 157-165. doi: 10.3233/jad-143231.

Wasco, W., Pettingell, W.P., Jondro, P.D., Schmidt, S.D., Gurubhagavatula, S., Rodes, L., et al. (1995). Familial Alzheimer's chromosome 14 mutations. *Nat Med* 1(9)**,** 848. doi: 10.1038/nm0995-848a.

Xia, M., Chen, S., Shi, Y., Huang, Y., Xu, J., Zhao, T., et al. (2015). Probable novel PSEN2 Pro123Leu mutation in a Chinese Han family of Alzheimer's disease. *Neurobiol Aging* 36(12)**,** 3334.e3313-3334.e3318. doi: 10.1016/j.neurobiolaging.2015.09.003.

Yagi, R., Miyamoto, R., Morino, H., Izumi, Y., Kuramochi, M., Kurashige, T., et al. (2014). Detecting gene mutations in Japanese Alzheimer's patients by semiconductor sequencing. *Neurobiol Aging* 35(7)**,** 1780.e1781-1785. doi: 10.1016/j.neurobiolaging.2014.01.023.

Yasuda, M., Maeda, K., Hashimoto, M., Yamashita, H., Ikejiri, Y., Bird, T.D., et al. (1999). A pedigree with a novel presenilin 1 mutation at a residue that is not conserved in presenilin 2. *Arch Neurol* 56(1)**,** 65-69. doi: 10.1001/archneur.56.1.65.

Yasuda, M., Maeda, K., Ikejiri, Y., Kawamata, T., Kuroda, S., and Tanaka, C. (1997). A novel missense mutation in the presenilin-1 gene in a familial Alzheimer's disease pedigree with abundant amyloid angiopathy. *Neurosci Lett* 232(1)**,** 29-32. doi: 10.1016/s0304-3940(97)00569-7.

Yasuda, M., Maeda, S., Kawamata, T., Tamaoka, A., Yamamoto, Y., Kuroda, S., et al. (2000). Novel presenilin-1 mutation with widespread cortical amyloid deposition but limited cerebral amyloid angiopathy. *J Neurol Neurosurg Psychiatry* 68(2)**,** 220-223. doi: 10.1136/jnnp.68.2.220.

Yescas, P., Huertas-Vazquez, A., Villarreal-Molina, M.T., Rasmussen, A., Tusié-Luna, M.T., López, M., et al. (2006). Founder effect for the Ala431Glu mutation of the presenilin 1 gene causing early-onset Alzheimer's disease in Mexican families. *Neurogenetics* 7(3)**,** 195-200. doi: 10.1007/s10048-006-0043-3.

Youn, Y.C., Bagyinszky, E., Kim, H., Choi, B.O., An, S.S., and Kim, S. (2014). Probable novel PSEN2 Val214Leu mutation in Alzheimer's disease supported by structural prediction. *BMC Neurol* 14**,** 105. doi: 10.1186/1471-2377-14-105.

Zarea, A., Charbonnier, C., Rovelet-Lecrux, A., Nicolas, G., Rousseau, S., Borden, A., et al. (2016). Seizures in dominantly inherited Alzheimer disease. *Neurology* 87(9)**,** 912-919. doi: 10.1212/wnl.0000000000003048.

Zekanowski, C., Styczyńska, M., Pepłońska, B., Gabryelewicz, T., Religa, D., Ilkowski, J., et al. (2003). Mutations in presenilin 1, presenilin 2 and amyloid precursor protein genes in patients with early-onset Alzheimer's disease in Poland. *Exp Neurol* 184(2)**,** 991-996. doi: 10.1016/s0014-4886(03)00384-4.

Zhan, Y., Zheng, H., Wang, C., Rong, Z., Xiao, N., Ma, Q., et al. (2017). A novel presenilin 1 mutation (F388L) identified in a Chinese family with early-onset Alzheimer's disease. *Neurobiol Aging* 50**,** 168.e161-168.e164. doi: 10.1016/j.neurobiolaging.2016.10.010.

Zhou, L., Brouwers, N., Benilova, I., Vandersteen, A., Mercken, M., Van Laere, K., et al. (2011). Amyloid precursor protein mutation E682K at the alternative β-secretase cleavage β'-site increases Aβ generation. *EMBO Mol Med* 3(5)**,** 291-302. doi: 10.1002/emmm.201100138.
